# Bottom-Up Design Approach for OBOC Peptide Libraries

**DOI:** 10.3390/molecules25153316

**Published:** 2020-07-22

**Authors:** Daniela Kalafatovic, Goran Mauša, Dina Rešetar Maslov, Ernest Giralt

**Affiliations:** 1 Department of Biotechnology, University of Rijeka, Radmile Matejčić 2, 51000 Rijeka, Croatia; dina.resetar@biotech.uniri.hr; 2 Faculty of Engineering, University of Rijeka, Vukovarska 58, 51000 Rijeka, Croatia; gmausa@riteh.hr; 3 Institute for Research in Biomedicine (IRB Barcelona), The Barcelona Institute of Science and Technology (BIST), Baldiri Reixac, 10, 08028 Barcelona, Spain; ernest.giralt@irbbarcelona.org; 4 Department of Inorganic and Organic Chemistry, University of Barcelona, Marti i Franques, 1-5, 08028 Barcelona, Spain

**Keywords:** OBOC peptide libraries, bottom-up design, UPLC-MS analysis, combinatorial

## Abstract

One-bead-one-compound peptide libraries, developed following the top-down experimental approach, have attracted great interest in the identification of potential ligands or active peptides. By exploiting a reverse experimental design approach based on the bottom-up strategy, we aimed to develop simplified, maximally diverse peptide libraries that resulted in the successful characterization of mixture components. We show that libraries of 32 and 48 components can be successfully detected in a single run using chromatography coupled to mass spectrometry (UPLC-MS). The proposed libraries were further theoretically evaluated in terms of their composition and physico-chemical properties. By combining the knowledge obtained on single libraries we can cover larger sequence spaces and provide a controlled exploration of the peptide chemical space both theoretically and experimentally. Designing libraries by using the bottom-up approach opens up the possibility of rationally fine-tuning the library complexity based on the available analytical methods.

## 1. Introduction

This paper, written for the special issue Peptides and Peptide Technologies: A Themed Issue in Honor of Professor Morten Meldal on the Occasion of His Citation Laureates, wants to pay tribute to his pioneering work on one-bead-one-compound (OBOC) peptide libraries [1,2,3,4,5,6] and development of advanced synthetic methodologies on solid supports [7,8,9,10,11].

OBOC combinatorial libraries allow the synthesis of large and diverse mixtures of peptide sequences and their concurrent screening for affinity interactions [1,2,12,13,14,15,16,17,18]. The four main steps employed in the development of OBOC peptide libraries are: (a) library synthesis, (b) screening against biological targets of interest, (c) deconvolution of the sequence, followed by (d) validation of peptide entities [19]. Several examples have been developed and characterized following this scheme [12,16,20,21,22]. In addition, a variety of optimized methods have been proposed to improve both synthesis and screening processes, such as the use of color coding [23], fluorescent dyes [17], immobilization of beads on solid supports [24] and others [3,14,18,21,25]. This typical top-down design approach to OBOC peptide libraries leads to the efficient synthesis of large libraries but often fails in the step of sequence identification after screening. The unambiguous confirmation of peptide sequences remains a major bottleneck due to mixture complexity [3,12,26]. Such complexity often hampers straightforward sequence elucidation due to the co-existence of palindromic or isobaric peptides, partial sequence overlapping and physico-chemical similarity of peptide permutations [27]. Consequently, families of peptides having identical masses (*m/z* values), rather than single sequences, are identified [26]. Moreover, during MS-based characterization, the ion suppression and the formation of peptide adducts may contribute to the complexity of the obtained mass spectra. Accordingly, efficient characterization of OBOC libraries using commonly established techniques, such as matrix-assisted laser desorption/ionization (MALDI)-based MS and ultra-high performance liquid chromatography coupled to mass spectrometry (UPLC-MS) becomes challenging by increasing the library size and sequence length of library components [4,28]. Therefore, there is a need to optimize the library analysis process to guarantee a better quality of the results.

**Scheme 1:**
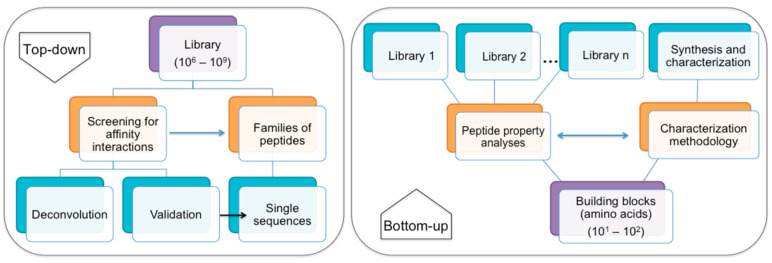
Top-down (left panel) and bottom-up (right panel) experimental design approaches to OBOC peptide libraries indicating different starting points (large libraries for top-down and small libraries for bottom-up) leading to diverse experimental validation protocols.

In this paper, we distinguish the top-down and the bottom-up approaches to OBOC peptide library design (Scheme 1). The top-down strategy of breaking down a system to gain insight into its components refers to the synthesis of large (10^6^–10^9^) random libraries with the intention of narrowing them down into families of sequences (10^1^) showing similar masses, physico-chemical properties and specific activities (e.g., binding). Such libraries cover large sequence spaces and concomitantly tend to expose targets to a large variety of possible ligands when screening for affinity interactions. Ultimately, the goal of this approach is to identify single sequences responsible for the desired activity. The identified “hits” can be further optimized to lead to efficient binders or active peptides [12,25]. In this work, rather than synthesizing large libraries, we propose the use of several smaller maximally diversified (10^1^–10^2^) ones that can be efficiently characterized by commonly established laboratory techniques (e.g., UPLC-MS).

For this purpose, we set out to determine whether a reverse experimental design approach based on the bottom-up strategy could lead to the efficient characterization of random peptide library mixtures by reducing the complexity of single libraries. Specifically, the focus was put on quality analysis of libraries designed by genetic algorithms to minimize sequence overlapping and thus facilitate their characterization by routinely available methods in research laboratories. The advantage of the proposed strategy is the obtainment of fully characterized smaller portions of the sequence space (composed of 10^1^–10^2^ components) that can consequently be combined to encompass larger portions of it. Additionally, it allows the controlled increase in library size and complexity, giving rise to rationally designed platforms able to provide more informative data on single sequence behavior.

## 2. Results and Discussion 

### 2.1. Bottom-Up Experimental Approach for the Design of OBOC Peptide Libraries

**Figure 1:**
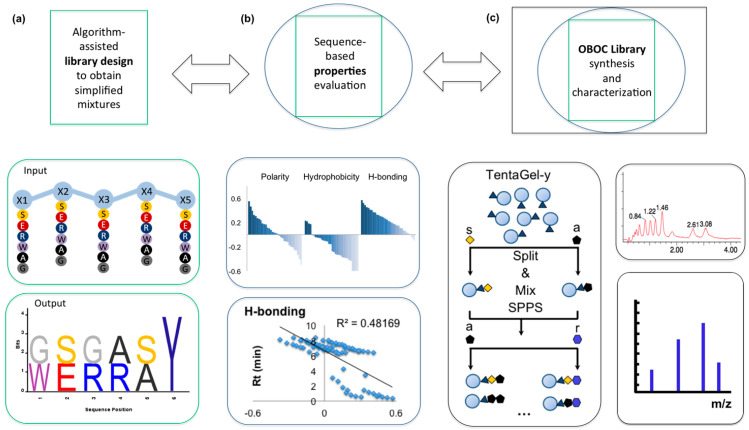
The steps of the proposed bottom-up experimental design methodology show, in (**a**) the input and output (sequence logo) of the GA-assisted design, (**b**) sequence-based properties obtained by theoretical peptide evaluation (e.g., Cruciani properties and linear regression for H-bonding for the 32-member library), followed by (**c**) the synthesis (split-and-mix approach) and LC-MS characterization.

The bottom-up experimental approach to OBOC peptide library design is based on the choice of the library complexity to match the available instrumental characteristics such as resolving power and mass accuracy. The goal is to obtain rapid design and efficient analysis of single peptide sequences from simplified mixtures that could be performed routinely after synthesis by UPLC-MS or other complementary techniques. This approach consists of three main steps schematically represented in Figure 1: (a) the choice of amino acids (input) to obtain random peptide library designs (output) [29], (b) sequence-based properties evaluation followed by (c) library synthesis and characterization. Having a smaller library does not necessarily solve the issue of overlapping peptides. Therefore, we implemented a search-based algorithm to find optimal solutions, maximal in size and minimal in overlapping.

To show the applicability of the bottom-up design approach, two libraries were designed and characterized. The first step was the choice of amino acids to be included in a specific library based on their properties that consequently dictate the properties of the library. In our previous work, we developed a genetic algorithm (GA) for OBOC peptide library design that produces random simplified libraries where it is possible to distinguish all components by their mass [29]. The parameters are the number of positions (*r*) i.e., peptide length, the list of possible residues for each position and the mass variability parameter (Δ*T*) [29]. We chose two algorithm-assisted designs for further experimental validation that consisted of five (Library 1) and six (Library 2) positions *r* where variability was introduced [29]. For Library 1, six amino acids (*s*,*e*,*r*,*w*,*a*,*G*) were proposed for each position *(x_1_–x_5_)* and one fixed residue (*y*) was used in the last position (*x_6_*), while for Library 2, seven amino acids (*s*,*e*,*r*,*w*,*a*,*G*,*i*) were put in each position and two fixed positions (*p,y*) were used (*x*_3_ and *x*_7_). This input is fed to the algorithm that produces a series of possible output options for library design [29]. The chosen output suggestions, from the 90–100% mass diversity region and for ΔT = 1 were:

(a) Library 1 (L1): {G, w} for *x*_1_, {s, e} for *x*_2_, {G, r} for *x*_3_, {a, r} for *x*_4_ and {s, a} for *x*_5_ and {y} for *x_6_* to obtain a 32-component library (sequence logo in Figure 1a), where all the sequences are unique by mass;
(b) Library 2 (L2): {a, w} for *x*_1_, {s, r} for *x*_2_, {p} for *x*_3_, {a, e} for *x*_4_, {i, e, s} for *x*_5_, {G, w} for x_6_ and {y} for *x_7_* to obtain a 48-component library (sequence logo in Figure S1b,), where 47 permutations are unique by mass and one shows mass overlapping.

Our focus was put on the maximum (above 90%) mass and sequence diversity region because we believe it would lead to a more straightforward chromatographic and mass analysis. In both examples, all-D peptide libraries were considered. Libraries were designed to contain amino acids important for protein–protein interactions with the aim to develop libraries that could be useful for screening of specific protein–protein or receptor–ligand interactions [26,30].

Following, sequence-based analysis of relevant properties such as hydrophobicity, polarity, stability, potential for interactions through H-bonding, etc., (Section 2.2) was performed [31]. Finally, split-and-mix synthesis and consecutive characterization, taking into account the type of analytical instrument or platform, its resolving power and the molecular complexity of the library, were carried out by a) UPLC-MS, b) direct-injection high-resolution MS to increase mass accuracy and high-throughput and c) nano LC-MS/MS to increase sensitivity, confirm the exact amino acid sequences and to offer the possibility to work with minimal amounts of sample (Section 2.3). Linear regression and Spearman’s correlation coefficient were used to analyze the relationship between experimentally (UPLC) determined retention times (Rt) and computed library properties.

### 2.2. Theoretical Evaluation of Library Properties

The composition and physico-chemical properties (Cruciani: polarity, hydrophobicity and H-bonding [32], hydrophobicity index, hydrophobic moment, aliphatic index, Boman index, net charge, isoelectric point and instability index) of the synthesized libraries were calculated using existing scripts from the R Peptide package [33,34]. To determine peptide composition, amino acids were classified based on their side chains into: tiny (A, C, G, S, T), small (A, C, D, G, N, P, S, T, V), aliphatic (A, I, L, V), aromatic (F, H, W, Y), non-polar (A, C, F, G, I, L, M, P, V, W, Y), polar (D, E, H, K, N, Q, R, S, T), charged (D, E, H, K, R), basic (H, K, R), acidic (D, E) [33]. We expanded the existing classification by adding two more classes: 10) sulfur (C, M) and 11) hydroxyl (S, T).

**Figure 2:**
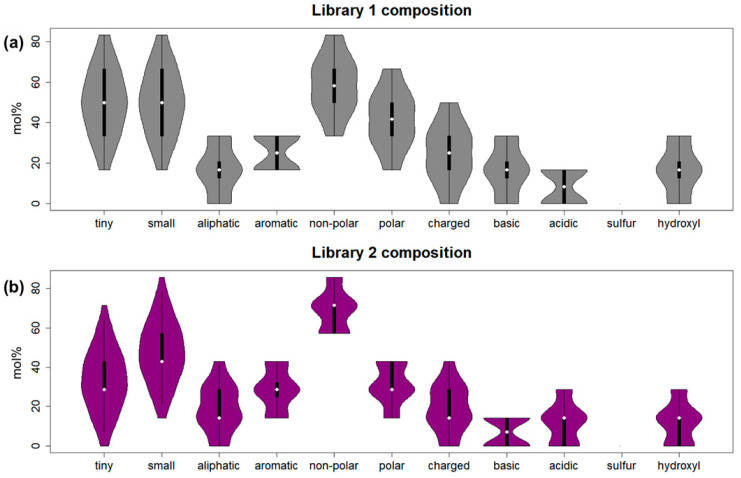
Violin plots depicting the composition of (**a**) L1 (gray) and (**b**) L2 (violet) where the relationship between classes for each library and the visual comparison of the two libraries are possible.

Composition calculations are graphically presented in Figure 2 where each property is expressed in mole% allowing for the direct comparison of classes of properties present in L1 and L2. The two libraries show similar composition with the main differences observed for the aliphatic, polar and non-polar classes. This was expected due to the fact the same amino acids (s, e, r, w, a, G, y) were used for library construction. Different combinations of them were employed to make up the sequences in each library, with L2 having additional isoleucine and proline residues responsible for contributing to differences in overall library polarity.

The following percentages of each amino acid were used to obtain the chosen libraries: 16.7% serine, 8.3% glutamic acid,16.7% arginine, 8.3% tryptophan, 16.7% alanine, 16.7% glycine and 16.7% tyrosine for L1 and 11.9% serine, 11.9% glutamic acid, 7.1% arginine, 14.3% tryptophan, 14.3% alanine, 7.1% glycine, 14.3% tyrosine, 4.8% isoleucine and 14.3% proline for L2 (Figure S1c).

**Figure 3:**
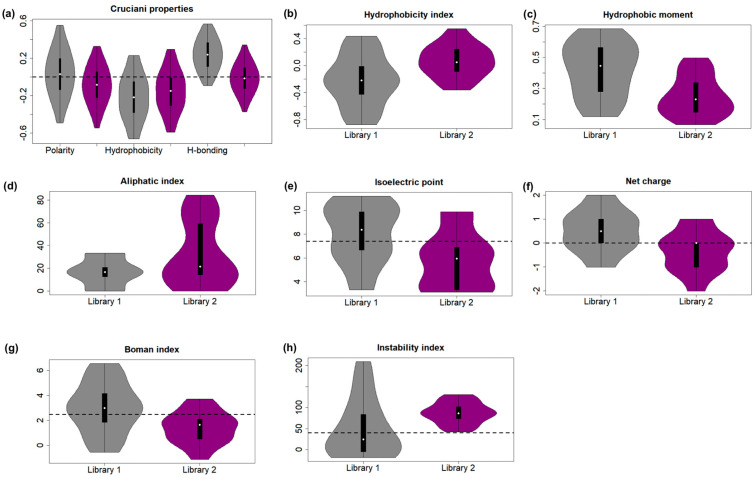
Violin plots showing the distribution of library properties: (**a**) Cruciani, (**b**) hydrophobicity index, (**c**) hydrophobic moment, (**d**) aliphatic index, (**e**) isoelectric point, (**f**) net charge, (**g**) Boman index and (**h**) instability index.

In addition, violin plots (Figure 3) were used to graphically compare the designed libraries in terms of other important physico-chemical properties. The Cruciani properties (Figure 3a) calculated for all the sequences in L1 (Figure 3a) and L2 (Figure 3b) show polarity, hydrophobicity and H-bonding distributions. Each descriptor is normalized from −1 to 1 to provide a straightforward visualization of the magnitude of each individual parameter. Sequences with positive polarity values are considered non-polar whereas negative values describe polar peptides. Sequences with hydrophobicity values above zero are considered large and hydrophobic, while the ones below zero are small and less hydrophobic. Finally, sequences showing positive values of H-bonding are considered H-acceptors and the negative ones are H-donors [32]. Therefore, we can conclude that L1 shows higher polarity and H-bonding acceptor propensity while having slightly lower hydrophobicity compared to L2.

Hydrophobicity is an important stabilization force in protein folding but also determines the chromatographic separation of peptides. Hydrophobicity index and hydrophobic moment (Figure 3b,c) show moderate values, with L2 being slightly more hydrophobic than L1. As expected, the aliphatic index (Figure 3d) is more prominent in L2 because of the presence of isoleucine and proline, absent in L1. Aliphatic amino acids contribute to the thermal stability of proteins [35].

The isoelectric point (IP) and the net charge are variables that affect the solubility of peptides under specific pH conditions. We considered these variables at the physiological pH. The designs for L1 (one E and two R residues) and L2 (two E and one R residues) suggest that libraries will show similar net charge and will be predominantly positively or negatively charged, respectively (Figure 3f). Accordingly, L1 shows the IPs in the range above the physiological pH, while L2 shows IPs below the physiological pH (Figure 3e).

The Boman index provided an estimation of the potential of a peptide to bind to other proteins [36]. The values above 2.4 indicate a high binding potential. It can be observed that L1 has an overall higher binding potential than L2 (Figure 3g).

The instability index calculations (Figure 3h) available for L-peptides, provided an estimation of the stability of the proposed library components based on the recognition of pairs of stable dipeptides [37]. According to this parameter, the all-L libraries resulted unstable by showing instability indices above the threshold value of 40 [31]. With the intention of creating more stable libraries, we opted for the synthesis of all-D peptide libraries. The OBOC method allows for easy and fast introduction of D- or other unnatural amino acids during library synthesis [1,2,13].

Properties-based chemical space evaluation is a valuable tool to gain insight into possible issues that could arise during the chromatographic separations. When using reverse-phase liquid chromatography, peptides are separated according to their hydrophobicity. A challenge lies in the fact that if peptides are too hydrophobic their detection might fail due to solubility issues, sustained non-covalent interactions with the stationary phase in the LC column or insufficient separation prior to MS. On the other hand, very hydrophilic peptides could face the problem of insufficient retention on the LC column prior to MS. A library of the same size but having a more hydrophilic composition would require LC method adaptation and a slower increase in solvent polarity gradient to allow for detection and separation of all peptide components. Therefore, the insight into hydrophobicity, polarity, net charge and other properties could anticipate the mentioned drawbacks that can be manipulated during the library design step in a bottom-up approach through the choice of amino acids.

Chromatographic separations provide useful information about analyte properties. Linear regression was used to analyze the relationship between experimentally (UPLC) determined retention times (Rt) and library composition calculated with R to understand which peptide properties influenced most the Rt. For this purpose, the calculated properties for both libraries were put together to cover a larger sequence space. Additional data on new libraries can be added to cover even larger sequence spaces with the goal to gain knowledge on growing portions of it. The obtained relationships (Figures S2, S3 and S4) indicate moderate linearity for monoisotopic mass (R^2^ = 0.48), Cruciani H-boding property (R^2^ = 0.48) and aromaticity (R^2^ = 0.44), a low level of linearity for the class of tiny (R^2^ = 0.3) and Cruciani polarity (R^2^ = 0.22) and no linearity for other properties (R^2^ < 0.15). As expected, it is not trivial to link a specific property to the Rt measurements [38], but a balance of different properties is involved.

Using a nonparametric statistical measure of Spearman’s correlation coefficient (ρ), which does not assume normality in data distribution, we analyzed the strength of the monotonic relationship between Rt and computed peptide properties (Figure S5). The Spearman’s coefficient is constrained in the range [−1, 1] and it is interpreted according to its absolute value as very strong (1.0–0.8), strong (0.8–0.6), moderate (0.6–0.4), weak (0.4–0.2) and very weak (0.2–0.0). A strong relationship was observed for H-bonding (ρ = −0.77), monoisotopic mass (ρ = 0.76), aromatic (ρ = 0.63) and tiny (ρ = −0.61), moderate relationship for basic (ρ = −0.55), Boman index (ρ = −0.55), polarity (ρ = 0.54), polar (ρ = −0.49) and non-polar (ρ = 0.49), while other properties exhibited weak and very weak relationships. Clearly, the relationship is more complex than a linear one and a plethora of peptide properties used together may better describe the libraries and could be used to assess the expected trend of Rt values.

### 2.3. Library Synthesis and Characterization by UPLC-MS, High-Resolution Mass Spectrometry and Nano LC-MS/MS

The synthesis of L1 and L2 was performed on monosized 30 µm TentaGel beads [9]. Peptide sequences were grown step-wise using the split-and-mix methodology, following the conventional Fmoc-SPPS protocol [39,40]. L1 had the first residue y (x_6_) preloaded and splitting into two equal parts was required for all the remaining positions to obtain the 32-member library with 100% variability (Figure 2). L2, had the first residue y (x_7_) preloaded and proline fixed in position x_3_, while positions x_1_, x_2_, x_4_ and x_6_ required splitting into two equal parts. Position x_5_ was split into three equal parts to obtain the 48-member library with 98% variability.

The next step was the assessment of the successful synthesis of the proposed libraries (Section 2.1). For this purpose, library components were released in solution prior to analysis using ammonia vapors to obtain amidated peptides at the C-terminus [9]. The library analysis was performed by UPLC-MS to determine whether we could detect all the 32 (L1) or 48 (L2) components in a single run based on their retention times and monoisotopic mass.

**Figure 4:**
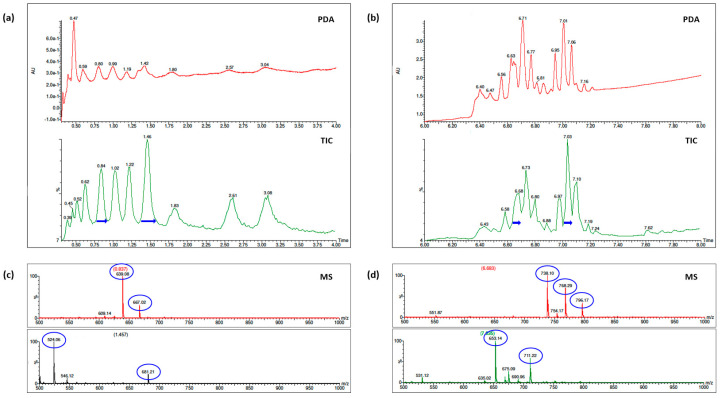
UPLC-MS data for L1, showing PDA (detection wavelength: 214 nm) and TIC chromatograms from (**a**) the zero min to four min and (**b**) the six min to eight min retention time regions. The arrows in the TIC chromatograms indicate the representative peaks for which the MS spectra are provided: Rt = 0.84 min ([M + H]^+^: 639.08 and 667.02), Rt = 1.46 min ([M + H]^+^: 524.05 and 681.21) in (**c**), Rt = 6.68 min ([M + H]^+^: 738.10, 768.29 and 796.17) and Rt = 7.03 min ([M + H]^+^: 653.14 and 711.22) in (**d**).

The UPLC-MS data for L1 (Figure 4), consisting of photodiode array (PDA) chromatograms and total ion chromatograms (TIC) were split into two regions, being zero to four minutes (Figure 4a) and six to eight minutes (Figure 4b) representing the time frames where all the peptides were detected (Figure S6a). In Figure 4c,d, MS spectra of nine representative peptides are shown. The full list of detected sequences alongside their retention times (Rt) is provided in Table S1 and Figure S7.

**Figure 5:**
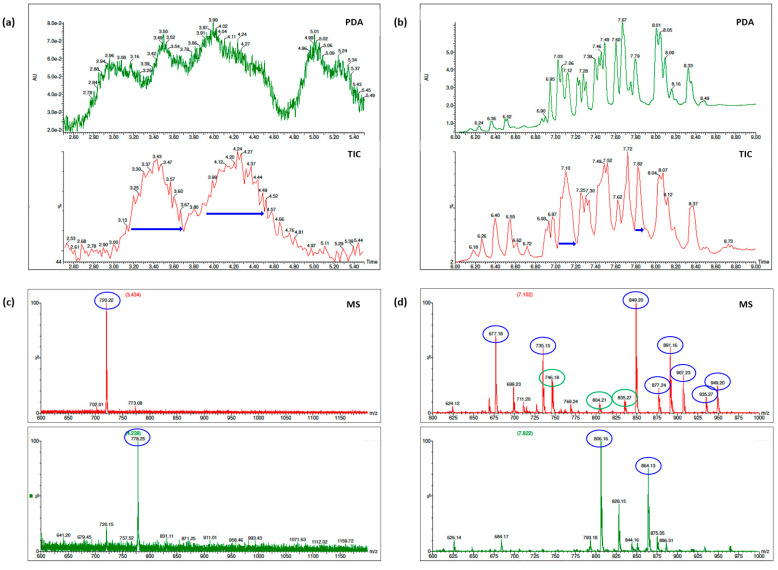
UPLC-MS data for L2, showing PDA (detection wavelength: 214 nm) and TIC chromatograms from (**a**) the 2.5 to 5.5 min and (**b**) the 6 to 9 min regions. The arrows in the TIC chromatograms indicate the representative peaks for which the MS spectra are provided: Rt = 3.43 min ([M + H]^+^: 720.22), Rt = 4.23 min ([M + H]^+^: 778.25) in (**c**); Rt = 7.10 min ([M + H]^+^: 677.18, 735.15, 849.20, 877.24, 891.16, 907.23, 935.27 and 949.20 in addition to 746.18, 804.21 and 835.27 more intense at other Rt values) and Rt = 7.82 min ([M + H]^+^: 806.16 and 864.13) in (**d**). The detection was at 214 nm.

The UPLC-MS data for L2 (Figure 5), consisting of the PDA and TIC chromatograms were split into two retention time regions, being 2 to 5.5 min (Figure 5a) and 6 to 9 min (Figure 5b) representing the time frames where all the peptides were detected (Figure S6b). The MS spectra of 15 representative peptides are shown in Figure 5c,d, while the full list of detected sequences alongside their retention times is provided in Table S2 and Figure S8.

In addition, high-resolution mass spectrometry analysis of L1 was performed by direct infusion on the LTQ-FT Ultra mass spectrometer to confirm the successful synthesis of all library components. All 32 peptides that fit with amidation on the C-terminus were detected in the sample. In Figures S9 and S10, the high-resolution MS spectra of *wsrasy* and *werasy* sequences are shown. For the full list of MS spectra of the L1 components please refer to the supplementary File 2.

Moreover, nanoscale liquid chromatography coupled to tandem mass spectrometry (nano LC-MS/MS) was used to validate selected sequences in L1. Examples are shown in Figures S11 and S12. Transitions corresponding to dissociation of the molecules at the peptide bonds b_3_ (430.2198), b_4_ (501.2569) and y_4_ (495.2674), b_5_ (588.2889) and y_5_ (582.2995) were found for *wsrasy*, while b_3_ (472.2303), b_4_ (543.2674) and y_4_ (495.2674), b_5_ (630.2995) and y_5_ (624.3100) were found for *werasy*.

Next, the mass accuracy of UPLC-MS and direct injection LTQ measurements were evaluated in terms of mean absolute error (MAE) and root mean square error (RMSE). The definitions of these two standard metrics are given in Table 1, where ${\hat{m}}_{i}$ represents the monoisotopic mass of the *i*-th peptide measured by the technique, $m_{i}$ represents the actual monoisotopic mass of the *i*-th peptide and *n* is the number of peptides within the library. The square expression used in RMSE makes this evaluation metric more sensitive to large errors, so it was used in addition to MAE to estimate the suitability of the used techniques. Having similar results in terms of both metrics, being 0.19–0.20 for UPLC-MS and 0.0007 for LTQ, we conclude that the majority of errors are similar in size. Moreover, the results presented in Table 1 clearly show that the LTQ is able to yield measurements that are three orders of magnitude more accurate than the ones obtained by the UPLC-MS.

Mass spectrometry-based analysis of peptides allows the identification of peptide molecules based on their monoisotopic masses for single or multiply charged ions, created during ionization in the ion source. The analysis of complex peptide mixtures is based on similar principles applied to single peptide analysis. The quality of the MS data depends upon the analytical mass accuracy and resolution. However, with the increase of sample complexity in terms of library size and sequence length, signals of single peptides might overlap and hamper the unambiguous assignments [41].

In the top-down strategy, several approaches have been used for the identification of peptide sequences from complex OBOC libraries with MALDI-based MS being the method of choice [16,42,43,44,45]. Frequently, the identified peptides were further analyzed with a variety of other, non-MS based methods, that included isothermal titration calorimetry (ITC), nuclear magnetic resonance (NMR), enzyme-linked immunosorbent based assays (ELISA) and surface plasmon resonance (SPR), with the intention to prove efficient interactions of identified peptides with their ligands. Nevertheless, most of the identified hits failed to provide conclusive data on ligand–target or peptide–protein interactions or showed a tendency to give false positives. This problem emerged during the ligand validation process, due to the inability to unambiguously identify sequences during the screening step [27]. This could arise from the inadequacy of the MS-based instrumental method to detect the exact permutation involved in the interaction rather than the most efficiently detectable sequences from the mixture, i.e., the sequences that are preferentially ionized using electrospray (ESI), MALDI or other ionization techniques.

Mass spectrometry is a powerful tool for the analysis of complex peptide libraries [9]. The high-resolution power of available instruments allows for the detection and successful resolving of thousands of peptides in a single spectrum [41]. It is a considerably faster method compared to LC-MS. However, the instruments are expansive and require highly skilled personnel to conduct such an analysis. Often, laboratories do not have such equipment. The bottom-up design approach proposed here would allow one to routinely characterize simplified libraries using UPLC-Q-MS. Therefore, the proposed reduction in sample complexity will lead to a higher probability of each individual peptide to be selected for fragmentation, i.e., detected.

## 3. Conclusions

In this paper, we proposed a novel bottom-up experimental design approach for OBOC peptide libraries based on the choice of the appropriate library complexity to match the instrument characteristics (i.e., resolution power, accuracy) and availability. Unlike previous attempts to synthesize large and complex libraries, we wanted to exploit the available instrumental techniques coupled to existing tools for peptide properties estimation to obtain precise and efficiently detectable peptide libraries.

The value of this strategy is the combination of the experimental data and the possibility to computationally help the design and evaluation of mixture properties. After evaluating the possible library designs and choosing the appropriate library complexity, sequence-related properties such as hydrophobicity, charge, isoelectric point and others could be assessed. Such a rational analysis of peptide libraries before synthesis allows us to anticipate possible challenges that could be encountered during synthesis or chromatographic and mass characterizations.

We showed that UPLC-MS, high-resolution MS and nano LC-MS/MS were suitable instruments for the analysis of the mixture of 32 peptides (L1). In addition, we characterized all 48 peptides (L2), including the two peptides with overlapping masses, in a single run using UPLC-MS. The obtained results confirmed the suitability of the proposed methodology for sequence elucidation and provided insight into allowed library complexity for the proposed methodology. Accordingly, when the available instrumentation and methods are suitable, the library complexity can be fine-tuned to match the analytical instrument characteristics. Hence, the complexity of the library will be increased through the algorithm-assisted library design only if the analytical resolving power allows for it. With this approach, we guarantee the possibility of efficiently detecting all the library components prior to more complex screenings. We envision that precise and conclusive data can be obtained that will lead to the successful identification of hits.

The goal was to create a platform for OBOC library design and characterization based on the bottom-up strategy starting with simplified peptide mixtures designed using genetic algorithms to obtain fully characterized portions of the explored sequence space. By combining the data obtained for several new libraries, we could expand our knowledge of the available sequence space. Thus, frustration linked to big screenings and inadequate analysis with inconclusive validation results could be avoided. However, the choice of the suitable design model, top-down or bottom-up, will ultimately depend on the nature of the specific scientific question and the laboratory resources available. We envision that the strategy proposed here will be of interest to a wider peptide chemistry community.

## 4. Materials and Methods

### 4.1. General

Fmoc-(D)-amino acids were purchased from Iris Biotech GmbH (Marktredwitz, Germany), while other reagents: 2-(1H-Benzotriazole-1-yl)-1,1,3,3-tetramethylaminium tetrafluoroborate (TBTU), *N*,*N′*-Diisopropylcarbodiimide (DIC), 4-(Dimethylamino)pyridine (DMAP), *N*,*N*-Diisopropylethylamine (DIPEA), triisopropylsilane (TIS) and Trifluoroacetic acid (TFA) were from Novabiochem (Merck, Darmstadt, Germany) and Sigma Aldrich (Merck, Darmstadt, Germany).

The resin (TentaGel^®^ M NH_2_ Monosized Amino TentaGel Microspheres, M30352) was obtained from Rapp Polymere GmbH (Tübingen, Germany). Other peptide synthesis reagents (piperidine) and solvents were reagent grade from Scharlau (Barcelona, Spain): methanol, dichloromethane (DCM), and Carlo Erba (Milan, Italy) reagents: *N*,*N*-Dimethylformamide (DMF). Solvents for chromatography (acetonitrile) were analytical grade obtained from Scharlau (Barcelona, Spain).

### 4.2. GA-Assisted Library Design

The libraries were designed using a Non-dominated Solutions Genetic Algorithm (NSGA-II), as reported previously [46]. Briefly, the following parameters of the GA model have been used: individual = single library, population size: 500, representation: bitstring (length depending on the input), generations: 100 * (multiplied by) bitstring length or 50 generations without an average relative change in the best fitness function value, crossover rate: 80%, crossover function: scattered, mutation rate: 1%, mutation function: bitflip, selection function: stochastic uniform, elitism: 5%, Pareto fraction: 20%, distance measure function: distance crowding, fitness functions: library size and percentage of peptides without mass overlap.

### 4.3. Solid-Phase Peptide Synthesis (SPPS) Procedure

The synthesis of peptide libraries was performed using the “split and mix method” [29]. All solid-phase manipulations were done manually in polypropylene syringes, each fitted with a polyethylene porous disk. All peptide sequences where grown step-wise using the standard Fmoc SPPS on TentaGel^®^ (particle size: 30 µm monosized; capacity: 0.24 mmol/g resin preloaded with the 4-hydroxymethylbenzoic acid (HMBA) linker followed by the first amino acid (Fmoc-Tyr(D)-OH) introduction through the ester bond formation. HMBA was coupled to the resin in DMF through a peptide bond, the same way as described below for the peptide chain growth, using three-fold excess of HMBA over the resin, TBTU and DIPEA. Following this step, Fmoc-Tyr(D)-OH was coupled in DCM using a 4-fold excess of amino acid, 4 equivalents of DIC and 0.4 equivalents of DMAP. The reaction was performed for 30 min and repeated three times.

The chain growth was performed with a three-fold excess of amino acid over the resin in DMF, using TBTU and DIPEA as activating and coupling reagents in 1:2 ratio respectively relative to the amino acid. Fmoc removal was carried out with 20% (*v/v*) piperidine in DMF.

The cleavage of the side chains was achieved using a cleavage cocktail: 95% TFA, 2.5 % TIS, and 2.5 % water. The library was extensively washed with water followed by washes in DMF, DCM and diethyl ether. The Kaiser colorimetric test assay was used for the detection of primary amines [39].

The cleavage from the resin of library peptides was achieved using the ammonia vapors to obtain peptides mixtures having NH_2_ at the C-terminal [40]. The peptide mixtures were then dissolved in 20% (*v/v*) acetonitrile/water mixtures, the beads were removed following centrifugation at 13,000 rpm for 30 min, the peptides in solution filtered and characterized by UPLC-MS, high-resolution mass spectrometry and nano LC-MS/MS to confirm the detection of all the library components.

### 4.4. Quantification of Resin Loading Capacity

In order to measure the loading capacity of the resin, piperidine washes were collected and measured by UV spectroscopy after coupling the first amino acid. After the Fmoc-Tyr(D)-OH peptide coupling to the HMBA-resin, Fmoc was removed in 5 mL 20% (*v/v*) piperidine/DMF, the supernatant collected and the Fmoc absorbance registered at 301 nm using the UV spectroscopy. The loading was calculated using the following equation: X = A · V · ε · m · b, where *X* is the loading of the resin, *A* is Fmoc absorbance at 301 nm, *V* is the volume of solvent, *ε* is the molar extinction coefficient of Fmoc at 301 nm (7800 M^−1^ cm^−1^), *m* is the mass of the resin in g and *b* the loading in mmol/g. The new loading was calculated to be 0.17 mmol/g.

### 4.5. The Spilt and Mix Method

L1 had the first residue y (x_6_) preloaded and splitting into two equal parts was required for all the remaining positions to obtain the 32-member library. L2 had the first residue y (x_7_) preloaded, proline fixed in position x_3_, while positions x_1_, x_2_, x_4_ and x_6_ required splitting into two equal parts. Position x_5_ was split into three equal parts to obtain the 48-member library. The splitting in two equal parts was achieved by adding 4 ml of DMF to the reaction vial (v1) containing the resin beads. Next, one 1 ml of bead suspension in DMF (v1) was added to each new vial i.e., 2 ml of bead suspension was distributed (1 ml per each vial) into two new reaction vials (v2 and v3). The following step consisted of the addition of 2 ml of DMF to the starting reaction vial (v1), taking out 2 ml (distributed 1 ml per each new vial) and repeating this process 4 more times until all the beads were transferred from v1 to v2 and v3. The splitting in three equal parts was performed by adding 6 ml of DMF to the reaction vial (v1), followed by distributing 3 ml of bead suspension i.e., 1 ml per each new vial (v2, v3, v4). The rest of the process was similar to the previously described one with the difference of the addition of 3 ml of DMF to the starting reaction vial (v1), taking 3 ml out (distributed 1 ml per each new vial) and repeating this process until all the beads were transferred from v1 to v2, v3 and v4. The chain growth was performed as described above (Section 4.3). After the addition of each new amino acid, the contents of the reaction vessels were mixed and Fmoc removal carried out. After the necessary washes, the splitting was repeated until the last amino acids were coupled. Following the completion of the synthesis, the beads were mixed together and cleaved from the resin.

### 4.6. PackageR Peptide Property Calculations

The *aaComp* function was implemented that classifies amino acids based on their size, side chains, hydrophobicity, charge and their response to pH 7 into 9 classes (tiny, small, aliphatic, aromatic, non-polar, polar, charged, basic, acidic). The script was modified to include two additional amino acid classes: sulfur and hydroxyl. In addition, library properties were assessed through Cruciani properties (polarity, hydrophobicity, H-bonds) [32], hydrophobicity index (Eisenberg scale), hydrophobic moment, aliphatic index, Boman index, net charge (Lehninger scale), isoelectric point (Lehninger scale) and instability index of the synthesized libraries were calculated using existing scripts from the Peptides package (version 2.4.1) written in R (link: https://github.com/dosorio/Peptides/) [33,34].

### 4.7. Ultra-High-Performance Liquid Chromatography-Quadrupole-Mass Spectrometry (UPLC-MS)

All peptides in the library fit with amidation at the C-terminus. The cleaved peptides were characterized by UPLC-MS (Waters high class) equipped with a photodiode array (PDA) detector, sample manager FNT, quaternary solvent manager and coupled to an electrospray ion source ESI-MS Micro mass ZQ, separated on a BEH C_18_-column (internal diameter, 2.1 mm; length, 50 mm; particle size, 1.7 μm) and analyzed using Masslynx 4.1 software. The flow rate was 0.6 mL/min using an isocratic flow at 0% MeCN, containing 0.07% formic acid (FA) as the ion-pair reagent, in the first 5 minutes followed by a linear gradient of 100% H_2_O, containing 0.1% FA, to 100% MeCN in the following 10 minutes. Detection was at 214 nm. The *m/z* range was set to 500 to 1000 or 600 to 1200 for L1 and L2, respectively. ESI positive ionization mode was used and the MS scan rate was set to 1 sec.

### 4.8. High-Resolution Mass Spectrometry

Sample preparation. The sample was 1/2 diluted with ACN + 1% formic acid.

MS Conditions. Mass Spectrometer: LTQ-FT Ultra (Thermo Fisher Scientific, Waltham, MA, USA). Sample introduction: Direct infusion (Automated Nanoelectrospray). The NanoMate (Advion BioSciences, Ithaca, NY, USA) aspirated the samples from a 384-well plate (protein Lobind) with disposable, conductive pipette tips, and infused the samples through the nanoESI Chip (which consists of 400 nozzles in a 20 × 20 array) into the mass spectrometer. Spray voltage was 1.70 kV and delivery pressure was 0.50 psi. Other conditions were as follows: nanoESI, positive ionization was used, *m/z* range: 100–2000 amu, capillary temperature: 200 °C, capillary voltage: 35 V, tube lens: 100 V.

Data Processing. Data was acquired with Xcalibur software, version 2.0 SP2 (Thermo Fisher Scientific, Waltham, MA, USA). Elemental compositions from experimental exact mass monoisotopic values were obtained with a dedicated algorithm integrated into Xcalibur software (2.0 SP2, Thermo Fisher Scientific, Waltham, MA, USA).

### 4.9. NanoLC-MS/MS Analysis

Chromatographs were acquired using nanoAcquity UPLC (Waters) after 1 μL injections. Nano LC Conditions were: column: trap loading, separation on column; trap column: 180 μm Å~ 2 cm C18 Symmetry (Waters); analytical column: BEH130™ C18 75 mm Å~ 25 cm, 1.7 μm (Waters), eluents: A. H2O 0.1% formic acid; B. CH_3_CN 0.1% formic acid; gradient: 1% to 35% of B in 60 min + 35% to 50% in 5 min + 50% to 85% in 2 min; flow rate: 250 nL/min.

LC-MS coupling. LC-MS coupling was performed with the Advion Triversa Nanomate (Advion BioSciences, Ithaca, NY, USA) as the nanoESI source performing nanoelectrospray through chip technology. The Nanomate was attached to an LTQ-FT Ultra mass spectrometer and operated at a spray voltage of 1.7 kV and a delivery pressure of 0.5 psi in positive mode.

MS conditions. LTQ-FT Ultra (Thermo Scientific) mass spectrometer was used with spray voltage set to 1.7 kV, capillary voltage: 40 V, capillary temperature: 200 °C, tube lens: 120 V, *m/z* range: 350–2000 amu and operating mode: data-dependent acquisition 1 MS1 + 6 MS2.

Data processing. LTQFT Ultra: Xcalibur software vs 2.0SR2 (Thermo Scientific).

## Figures and Tables

**Table 1:** Measurement accuracy shown through mean absolute and root mean square errors.

Evaluation Measure		Measurement Technique	
Abbreviation	Formula	UPLC-MS	**LTQ**
MAE	$\frac{1}{n}\overset{n}{\underset{i=1}{\sum }}\left|{\hat{m}}_{i}-m_{i}\right|$	0.1917	0.0007
RMSE	$\sqrt{\frac{1}{n}\overset{n}{\underset{i=1}{\sum }}{\left({\hat{m}}_{i}-m_{i}\right)}^{2}}$	0.2054	0.0007

## Supplementary Materials

The following are available online. Supplementary materials (Figures S1–12 and Tables S1 and S2) and Supplementary materials 2.

## References

[1] Meldal M. (2002). The one-bead two-compound assay for solid phase screening of combinatorial libraries. Biopolym.-Pept. Sci. Sect..

[2] Christensen C., Groth T., Schiødt C.B., Foged N.T., Meldal M. (2003). Automated Sorting of Beads from a “One-Bead-Two-Compounds” Combinatorial Library of Metalloproteinase Inhibitors. QSAR Comb. Sci..

[3] Madsen D., Jørgensen F.P., Palmer D., Roux M., Olsen J.V., Bols M., Schoffelen S., Diness F., Meldal M. (2020). Design and Combinatorial Development of Shield-1 Peptide Mimetics Binding to Destabilized FKBP12. ACS Comb. Sci..

[4] Hu H., Nikitin S., Berthelsen A.B., Diness F., Schoffelen S., Meldal M. (2018). Sustainable Flow Synthesis of Encoded Beads for Combinatorial Chemistry and Chemical Biology. ACS Comb. Sci..

[5] Meldal M. (2004). “One bead two compound libraries” for detecting chemical and biochemical conversions. Curr. Opin. Chem. Biol..

[6] Li M., Hoeck C., Schoffelen S., Gotfredsen C.H., Meldal M. (2016). Specific Electrostatic Molecular Recognition in Water. Chem.-A Eur. J..

[7] Meldal M. (1992). Pega: A flow stable polyethylene glycol dimethyl acrylamide copolymer for solid phase synthesis. Tetrahedron Lett..

[8] Meldal M. (2008). Polymer “clicking” by CuAAC reactions. Macromol. Rapid Commun..

[9] Hansen J., Diness F., Meldal M. (2016). C-Terminally modified peptides via cleavage of the HMBA linker by O-, N- or S-nucleophiles. Org. Biomol. Chem..

[10] Tornøe C.W., Christensen C., Meldal M. (2002). Peptidotriazoles on solid phase: [1,2,3]-Triazoles by regiospecific copper(I)-catalyzed 1,3-dipolar cycloadditions of terminal alkynes to azides. J. Org. Chem..

[11] Meldal M., Christensen S.F. (2010). Microparticle matrix encoding of beads. Angew. Chemie-Int. Ed..

[12] Liu R., Li X., Xiao W., Lam K.S. (2017). Tumor-targeting peptides from combinatorial libraries. Adv. Drug Deliv. Rev..

[13] Lam K.S., Salmon S.E., Hersh E.M., Hruby V.J., Kazmierski W.M., Knapp R.J. (1991). A new type of synthetic peptide library for identifying ligand-binding activity. Nature.

[14] Martínez-Ceron M.C., Giudicessi S.L., Saavedra S.L., Gurevich-Messina J.M., Erra-Balsells R., Albericio F., Cascone O., Camperi S.A. (2016). Latest Advances in OBOC Peptide Libraries. Improvements in Screening Strategies and Enlarging the Family From Linear to Cyclic Libraries. Curr. Pharm. Biotechnol..

[15] Gurevich-Messina J.M., Giudicessi S.L., Martínez-Ceron M.C., Acosta G., Erra-Balsells R., Cascone O., Albericio F., Camperi S.A. (2015). A simple protocol for combinatorial cyclic depsipeptide libraries sequencing by matrix-assisted laser desorption/ionisation mass spectrometry. J. Pept. Sci..

[16] Martínez-Ceron M.C., Giudicessi S.L., Marani M.M., Albericio F., Cascone O., Erra-Balsells R., Camperi S.A. (2010). Sample preparation for sequencing hits from one-bead-one-peptide combinatorial libraries by matrix-assisted laser desorption/ionization time-of-flight mass spectrometry. Anal. Biochem..

[17] Marani M.M., Ceron M.C.M., Giudicessi S.L., De Oliveira E., Côté S., Erra-Balsells R., Albericio F., Cascone O., Camperi S.A. (2009). Screening of one-bead-one-peptide combinatorial library using red fluorescent dyes. Presence of positive and false positive beads. J. Comb. Chem..

[18] Giudicessi S.L., Gurevich-Messina J.M., Martínez-Ceron M.C., Erra-Balsells R., Albericio F., Cascone O., Camperi S.A. (2013). Friendly strategy to prepare encoded one bead-one compound cyclic peptide library. ACS Comb. Sci..

[19] Bononi F.C., Luyt L.G., Derda R. (2015). Synthesis and Cell-Based Screening of One-Bead-One- Compound Peptide Libraries. Peptide Libraries: Methods and Protocols, Methods in Molecular Biology.

[20] Liu R., Marik J., Lam K.S. (2002). A novel peptide-based encoding system for “one-bead one-compound” peptidomimetic and small molecule combinatorial libraries. J. Am. Chem. Soc..

[21] Cho C.-F., Behnam Azad B., Luyt L.G., Lewis J.D. (2013). High-throughput screening of one-bead-one-compound peptide libraries using intact cells. ACS Comb. Sci..

[22] Marani M.M., Oliveira E., Côte S., Camperi S.A., Albericio F., Cascone O. (2007). Identification of protein-binding peptides by direct matrix-assisted laser desorption ionization time-of-flight mass spectrometry analysis of peptide beads selected from the screening of one bead-one peptide combinatorial libraries. Anal. Biochem..

[23] Luo J., Zhang H., Xiao W., Kumaresan P.R., Shi C., Pan C.X., Aina O.H., Lam K.S. (2008). Rainbow beads: A color coding method to facilitate high-throughput screening and optimization of one-bead one-compound combinatorial libraries. J. Comb. Chem..

[24] Xiao W., Bononi F.C., Townsend J., Li Y., Liu R., Lam K.S. (2013). Immobilized OBOC combinatorial bead array to facilitate multiplicative screening. Comb. Chem. High Throughput Screen.

[25] Cho C.F., Amadei G.A., Breadner D., Luyt L.G., Lewis J.D. (2012). Discovery of novel integrin ligands from combinatorial libraries using a multiplex “beads on a bead” approach. Nano Lett..

[26] Guixer B., Arroyo X., Belda I., Sabidó E., Teixidó M., Giralt E. (2016). Chemically synthesized peptide libraries as a new source of BBB shuttles. Use of mass spectrometry for peptide identification. J. Pept. Sci..

[27] Wright T., Gillet V.J., Green D.V.S., Pickett S.D. (2003). Optimizing the size and configuration of combinatorial libraries. J. Chem. Inf. Comput. Sci..

[28] Zhao P.L., Nachbar R.B., Bolognese J.A., Chapman K. (1996). Two new criteria for choosing sample size in combinatorial chemistry. J. Med. Chem..

[29] Kalafatovic D., Mauša G., Todorovski T., Giralt E. (2019). Algorithm-supported, mass and sequence diversity-oriented random peptide library design. J. Cheminform..

[30] Bogan A.A., Thorn K.S. (1998). Anatomy of hot spots in protein interfaces. J. Mol. Biol..

[31] Torres R., Osorio M.D. Package ‘Peptides’ 2018. https://github.com/dosorio/Peptides/.

[32] Cruciani G., Baroni M., Carosati E., Clementi M., Valigi R., Clementi S. (2004). Peptide studies by means of principal properties of amino acids derived from MIF descriptors. J. Chemom..

[33] Osorio D., Rondón-Villarreal P., Torres R. (2015). Peptides: A package for data mining of antimicrobial peptides. R J..

[34] Rice P., Longden L., Bleasby A. (2000). EMBOSS: The European Molecular Biology Open Software Suite. Trends Genet..

[35] Akai A. (1980). Thermostability and Aliphatic Index of Globular Proteins. J. Biochem..

[36] Boman H.G. (2003). Antibacterial peptides: Basic facts and emerging concepts. J. Intern. Med..

[37] Guruprasad K., Reddy B.V.B., Pandit M.W. (1990). Correlation between stability of a protein and its dipeptide composition: A novel approach for predicting in vivo stability of a protein from its primary sequence. Protein Eng. Des. Sel..

[38] Yoshida M., Hinkley T., Tsuda S., Abul-Haija Y.M., McBurney R.T., Kulikov V., Mathieson J.S., Galiñanes Reyes S., Castro M.D., Cronin L. (2018). Using Evolutionary Algorithms and Machine Learning to Explore Sequence Space for the Discovery of Antimicrobial Peptides. Chem.

[39] Furka Á., Sebestyén F., Asgedom M., Dibó G. (1991). General method for rapid synthesis of multicomponent peptide mixtures. Int. J. Pept. Prot. Res..

[40] Kaiser E., Colescott R.L., Bossinger C.D., Cook P.I. (1970). Color Test for Detection of Free Terminal Amino Groups in the Solid-Phase Synthesis of Peptides. Anal. Biochem..

[41] Palmblad M., Drijfhout J.W., Deelder A.M. (2010). High resolution mass spectrometry for rapid characterization of combinatorial peptide libraries. J. Comb. Chem..

[42] Scott Youngquist R., Fuentes G.R., Lacey M.P., Keough T., Baillie T.A. (1994). Matrix-assisted laser desorption ionization for rapid determination of the sequences of biologically active peptides isolated from support-bound combinatorial peptide libraries. Rapid Commun. Mass Spectrom..

[43] Semmler A., Weber R., Przybylsk M., Wittmann V. (2010). De Novo Sequencing of Peptides on SingleResin Beads by MALDI-FTICR Tandem MassSpectrometry. J. Am. Soc. Mass Spectrom..

[44] Joo S.H., Xiao Q., Ling Y., Gopishetty B., Pei D. (2006). High-throughput sequence determination of cyclic peptide library members by partial Edman degradation/mass spectrometry. J. Am. Chem. Soc..

[45] Paulick M.G., Hart K.M., Brinner K.M., Tjandra M., Charych D.H., Zuckermann R.N. (2006). Cleavable hydrophilic linker for one-bead-one-compound sequencing of oligomer libraries by tandem mass spectrometry. J. Comb. Chem..

[46] Tholey A., Becker A. (2017). Top-down proteomics for the analysis of proteolytic events-Methods, applications and perspectives. Biochim. Biophys. Acta-Mol. Cell Res..

