# Supplementary material for: Bottom-Up Design Approach for OBOC Peptide Libraries
Source: Molecules. 2020 Jul 22;25(15):3316. doi: 10.3390/molecules25153316 (PMC7435479; doi:10.3390/molecules25153316)

**GsGaay**

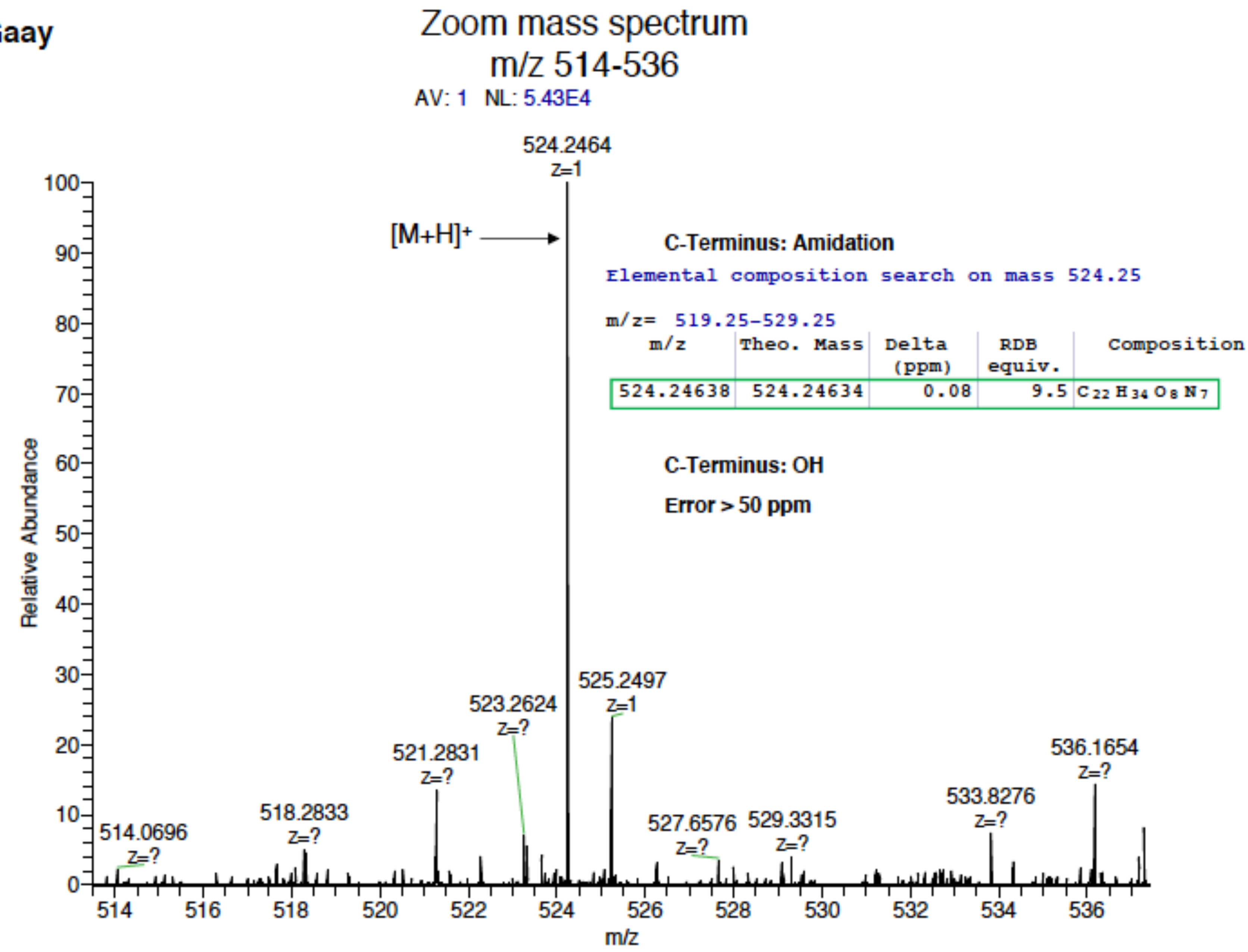

Zoom mass spectrum
m/z 514-536
AV: 1 NL: 5.43E4
524.2464
z=1
[M+H]+
C-Terminus: Amidation
Elemental composition search on mass 524.25
m/z= 519.25-529.25
m/z
Theo. Mass
Delta (ppm)
RDB equiv.
Composition
524.24638
524.24634
0.08
9.5
C22 H34 O8 N7
C-Terminus: OH
Error > 50 ppm
Relative Abundance
m/z
514.0696
z=?
518.2833
z=?
521.2831
z=?
523.2624
z=?
525.2497
z=1
527.6576
z=?
529.3315
z=?
533.8276
z=?
536.1654
z=?

**GsGasy**

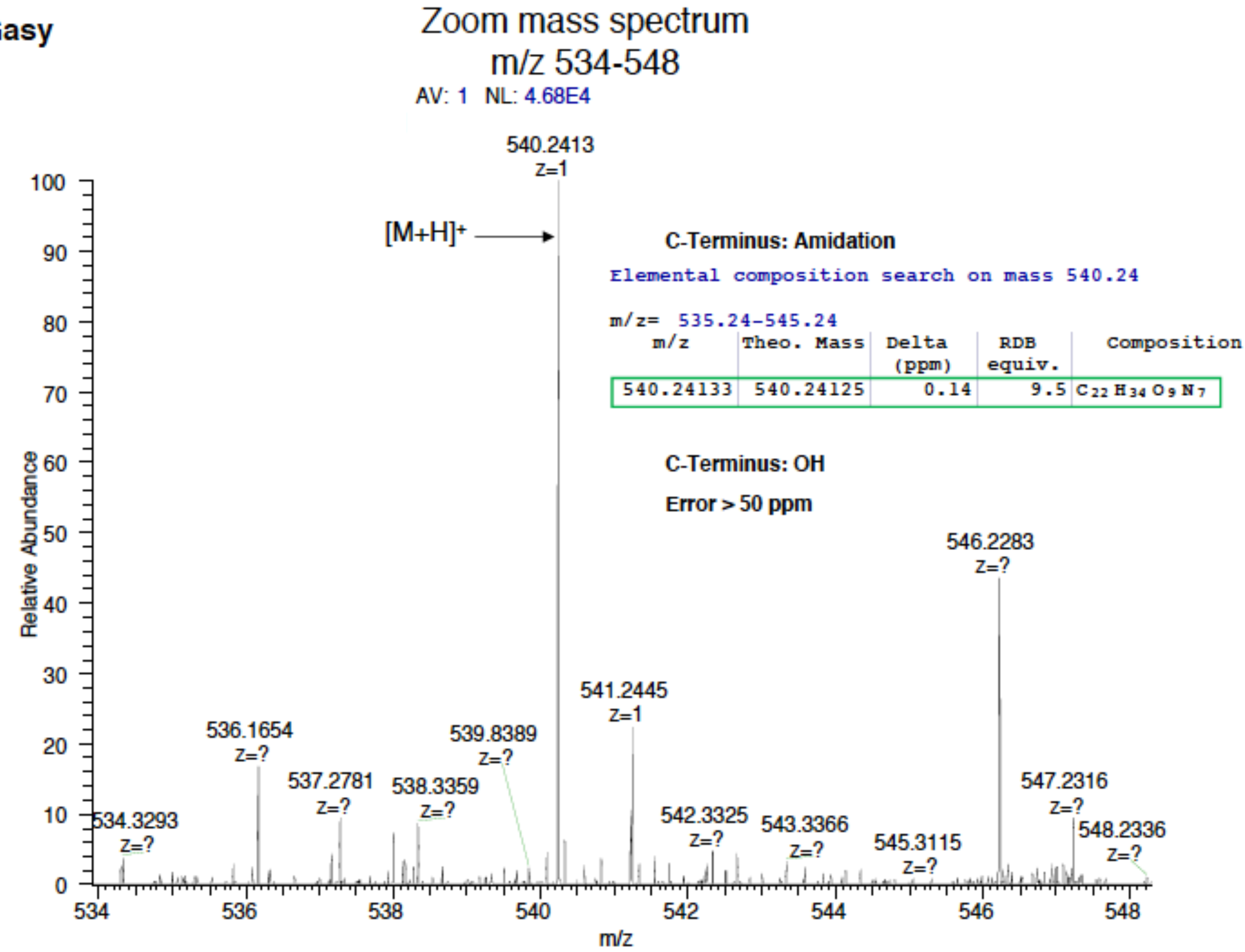
Zoom mass spectrum
m/z 534-548
AV: 1 NL: 4.68E4
540.2413
z=1
[M+H]+
C-Terminus: Amidation
Elemental composition search on mass 540.24
m/z= 535.24-545.24
m/z | Theo. Mass | Delta (ppm) | RDB equiv. | Composition
540.24133 | 540.24125 | 0.14 | 9.5 | C22 H34 O9 N7
C-Terminus: OH
Error > 50 ppm
546.2283
z=?
541.2445
z=1
536.1654
z=?
539.8389
z=?
537.2781
z=?
538.3359
z=?
547.2316
z=?
534.3293
z=?
542.3325
z=?
543.3366
z=?
548.2336
z=?
545.3115
z=?
Relative Abundance
m/z

**GeGaay**

## Zoom mass spectrum
## m/z 556-580

AV: 1 NL: 6.19E4

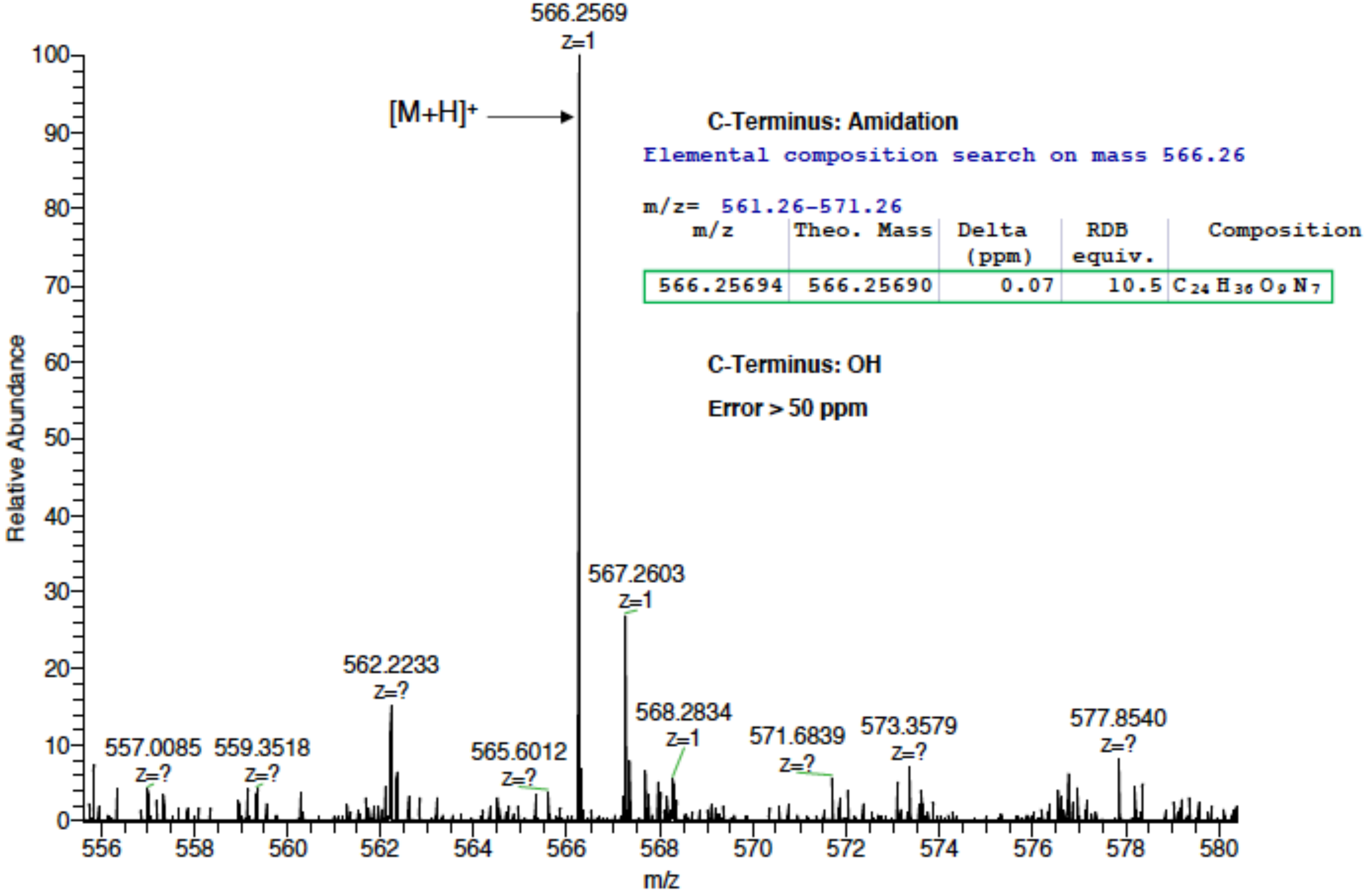


**C-Terminus: Amidation**

Elemental composition search on mass 566.26

m/z= 561.26-571.26

| m/z | Theo. Mass | Delta (ppm) | RDB equiv. | Composition |
|---|---|---|---|---|
| 566.25694 | 566.25690 | 0.07 | 10.5 | $C_{24}H_{36}O_9N_7$ |

**C-Terminus: OH**

**Error > 50 ppm**

**GeGasy**

## Zoom mass spectrum
## m/z 575-592

AV: 1 NL: 5.15E4

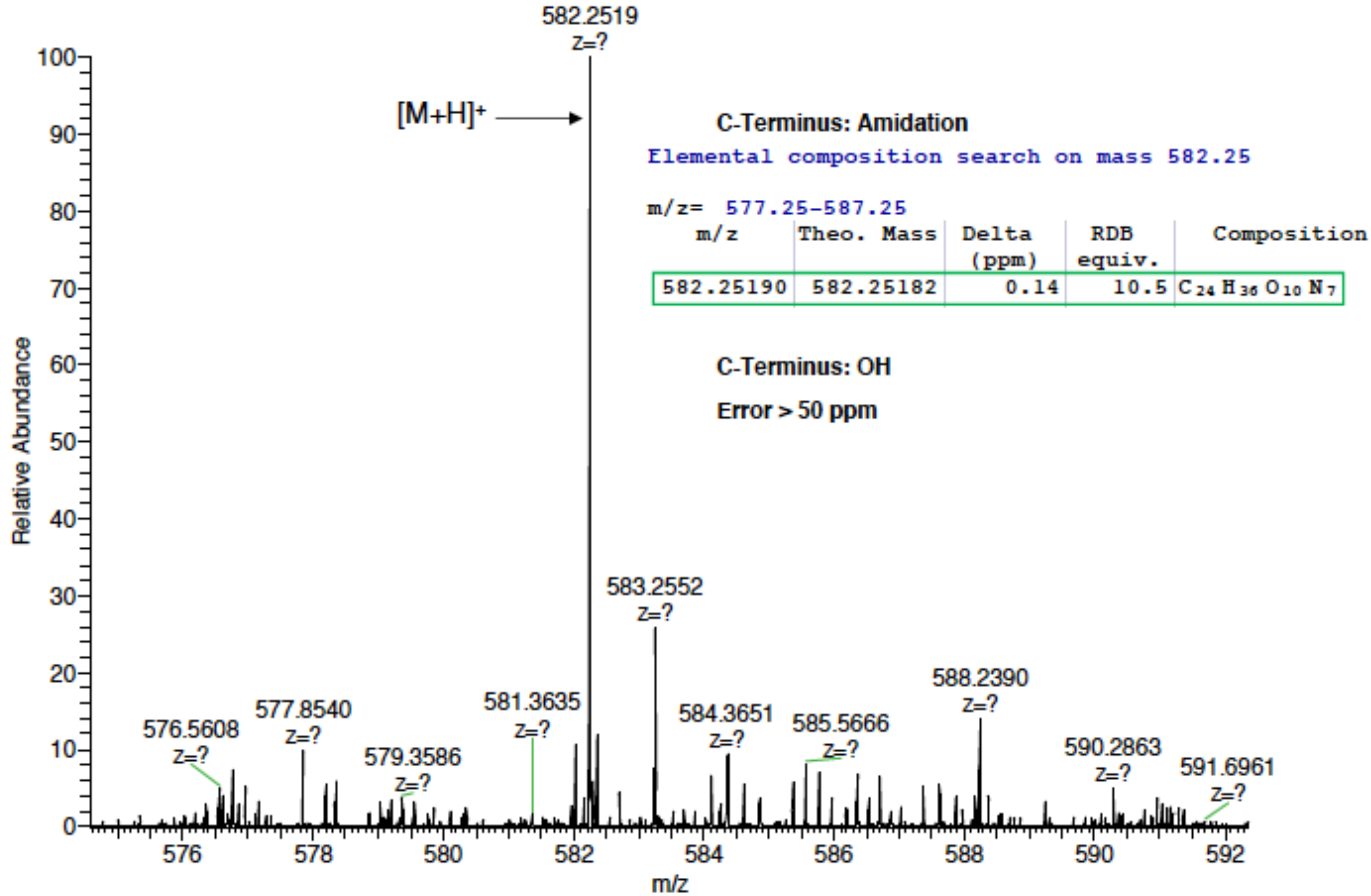


**C-Terminus: Amidation**

Elemental composition search on mass 582.25

m/z= 577.25-587.25

| m/z | Theo. Mass | Delta (ppm) | RDB equiv. | Composition |
|---|---|---|---|---|
| 582.25190 | 582.25182 | 0.14 | 10.5 | $C_{24}H_{36}O_{10}N_7$ |

**C-Terminus: OH**

**Error > 50 ppm**

**GsGray**

# Zoom mass spectrum
# m/z 600-621

AV: 1 NL: 6.31E4

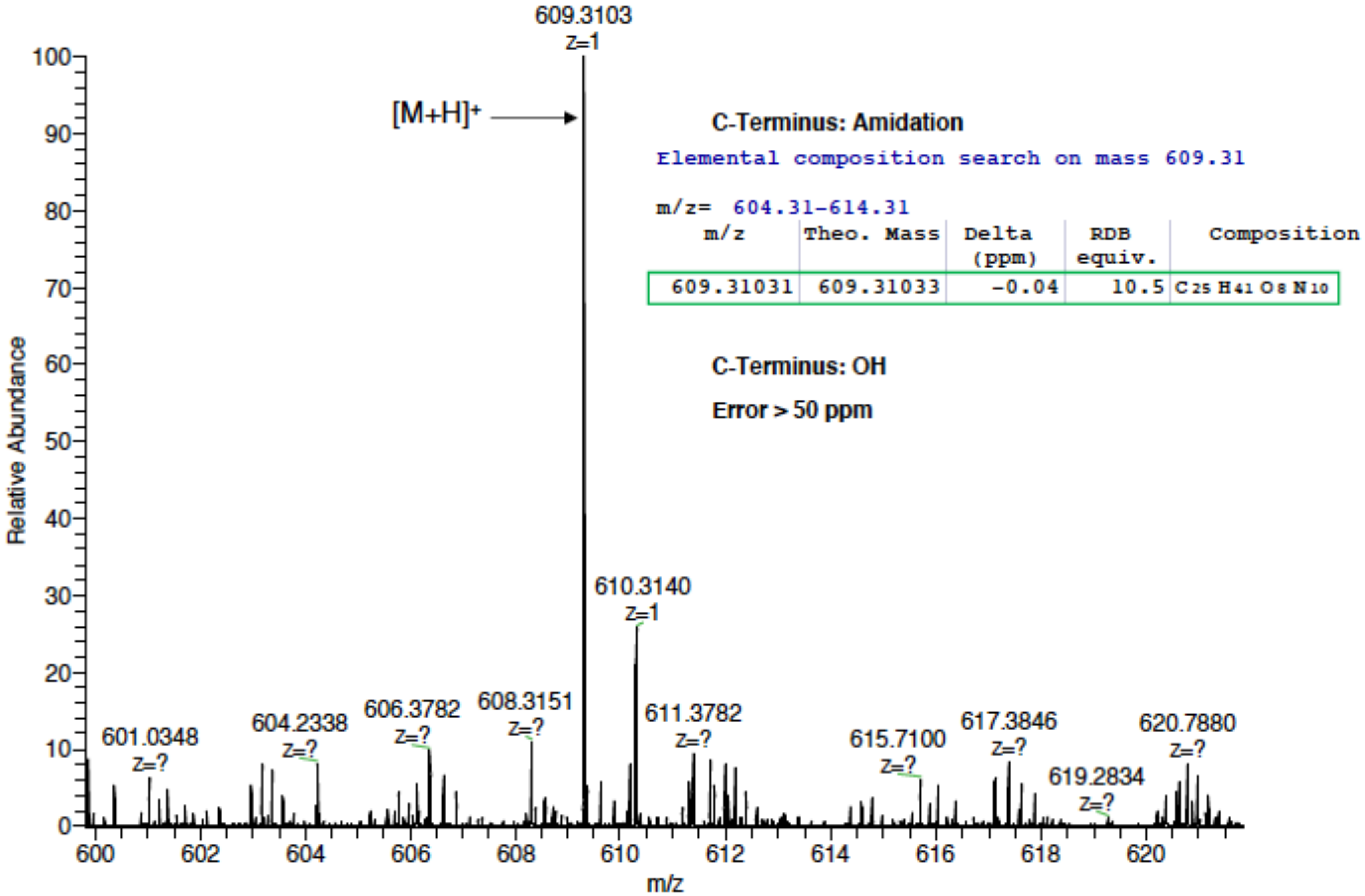

**Gsraay**

Zoom mass spectrum
m/z 610-633
AV: 1 NL: 1.10E5

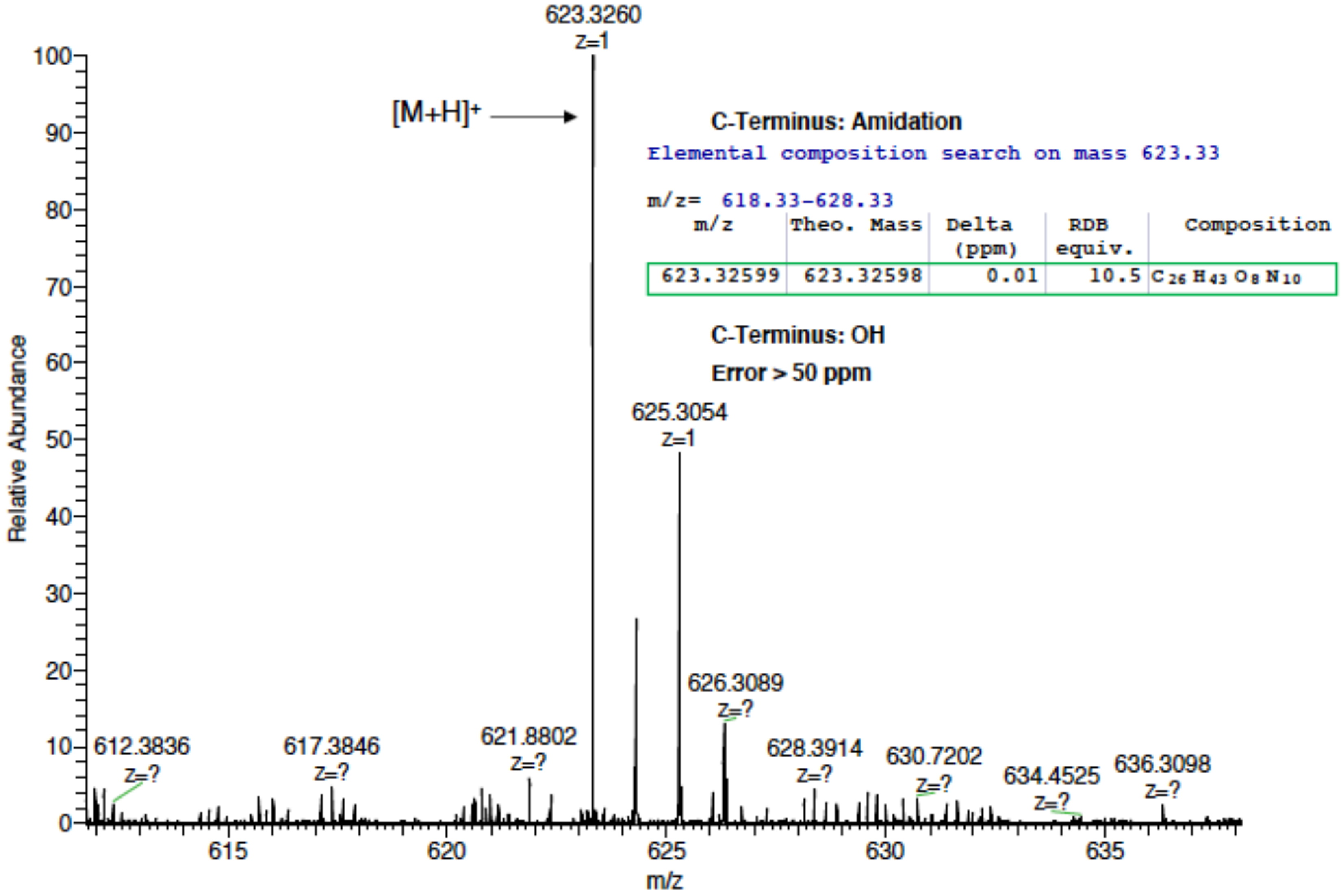


**C-Terminus: Amidation**

Elemental composition search on mass 623.33

m/z= 618.33-628.33

| m/z | Theo. Mass | Delta (ppm) | RDB equiv. | Composition |
|---|---|---|---|---|
| 623.32599 | 623.32598 | 0.01 | 10.5 | $C_{26}H_{43}O_8N_{10}$ |

**C-Terminus: OH**

**Error > 50 ppm**

**GsGrsy**

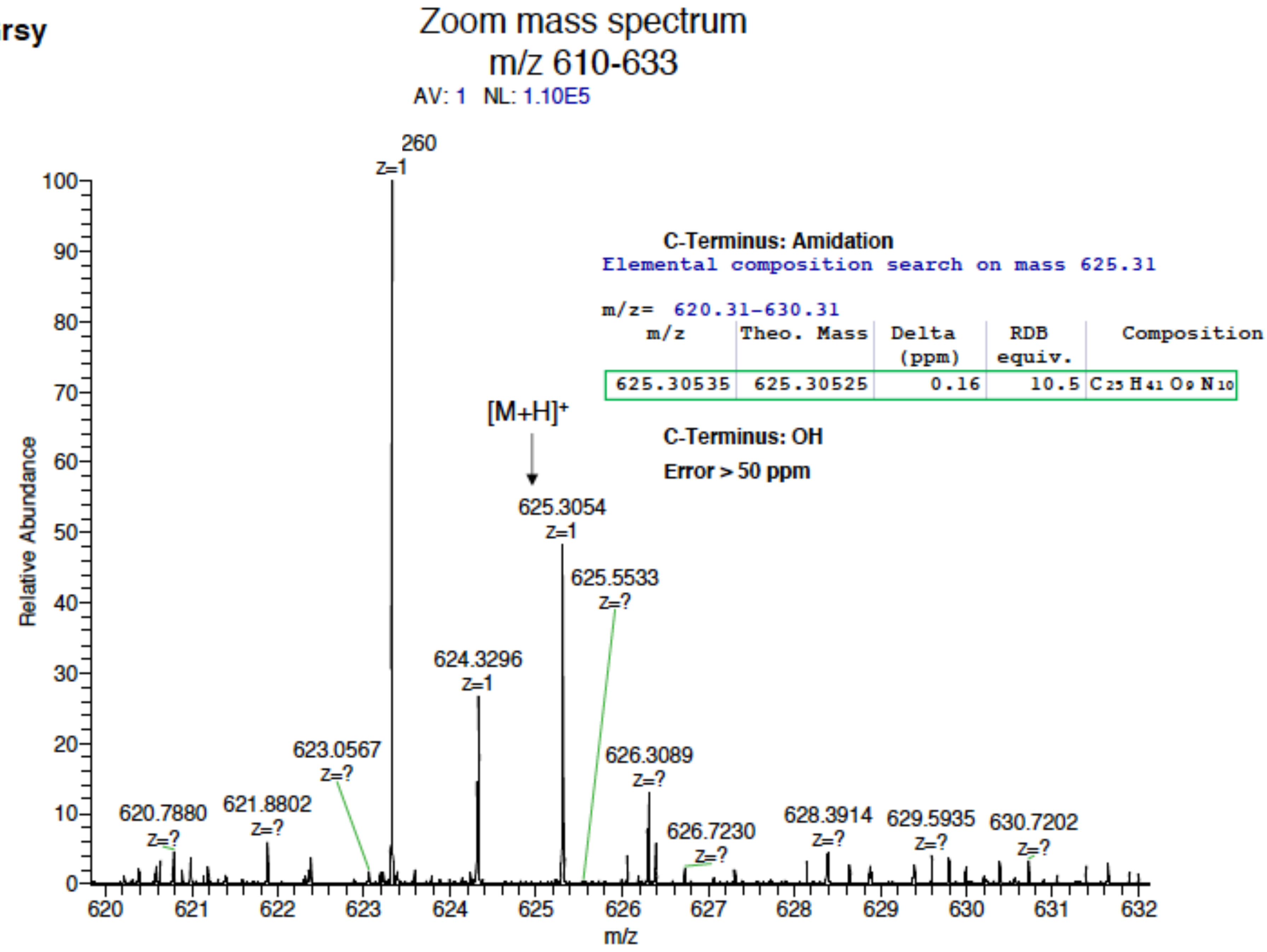


| m/z | Theo. Mass | Delta (ppm) | RDB equiv. | Composition |
|---|---|---|---|---|
| 625.30535 | 625.30525 | 0.16 | 10.5 | $C_{25}H_{41}O_9N_{10}$ |

**Gsrasy**

## Zoom mass spectrum
## m/z 630-650

AV: 1 NL: 8.93E4

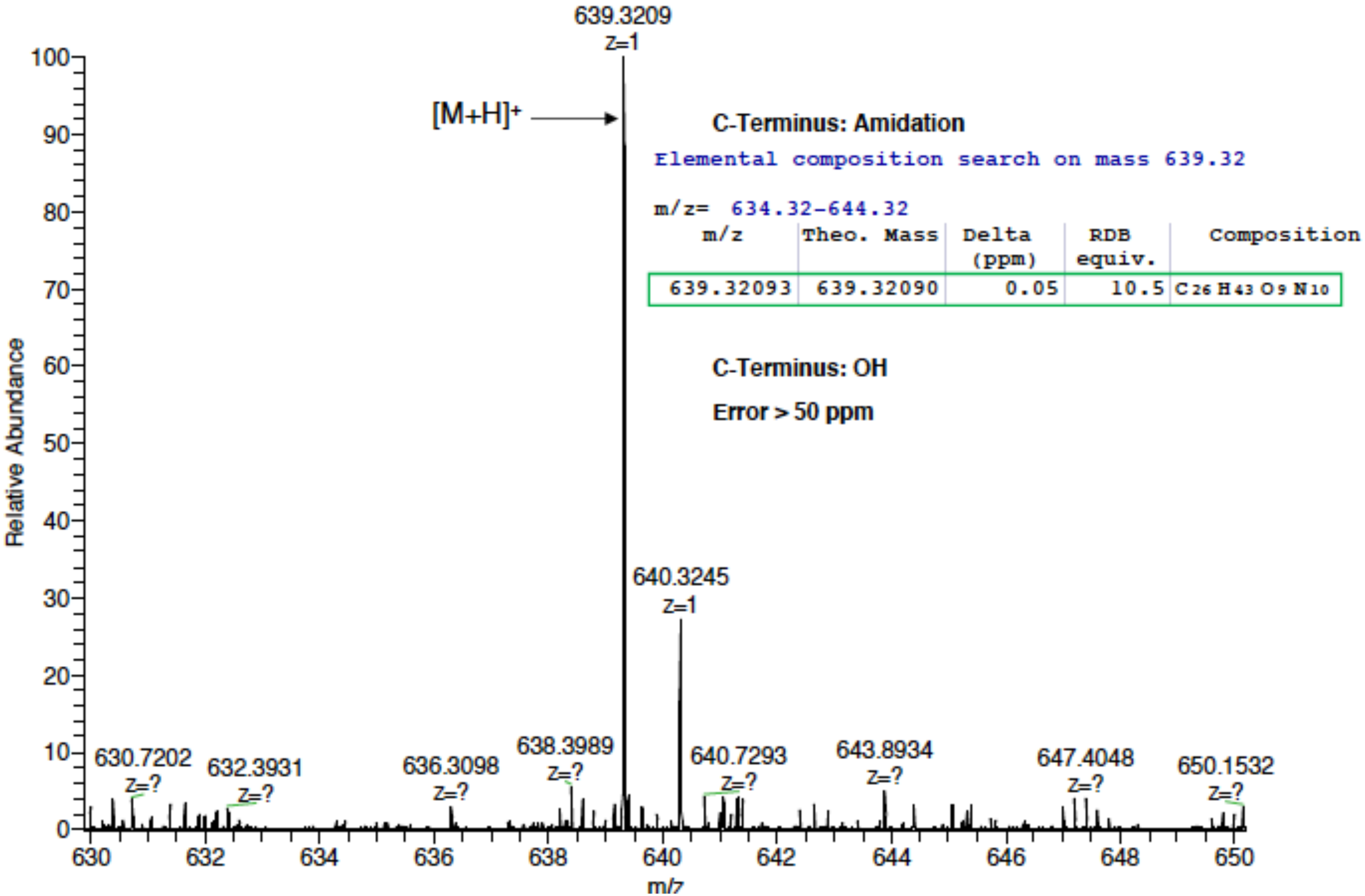


| m/z | Theo. Mass | Delta (ppm) | RDB equiv. | Composition |
|---|---|---|---|---|
| 639.32093 | 639.32090 | 0.05 | 10.5 | $C_{26}H_{43}O_9N_{10}$ |

**GeGray**

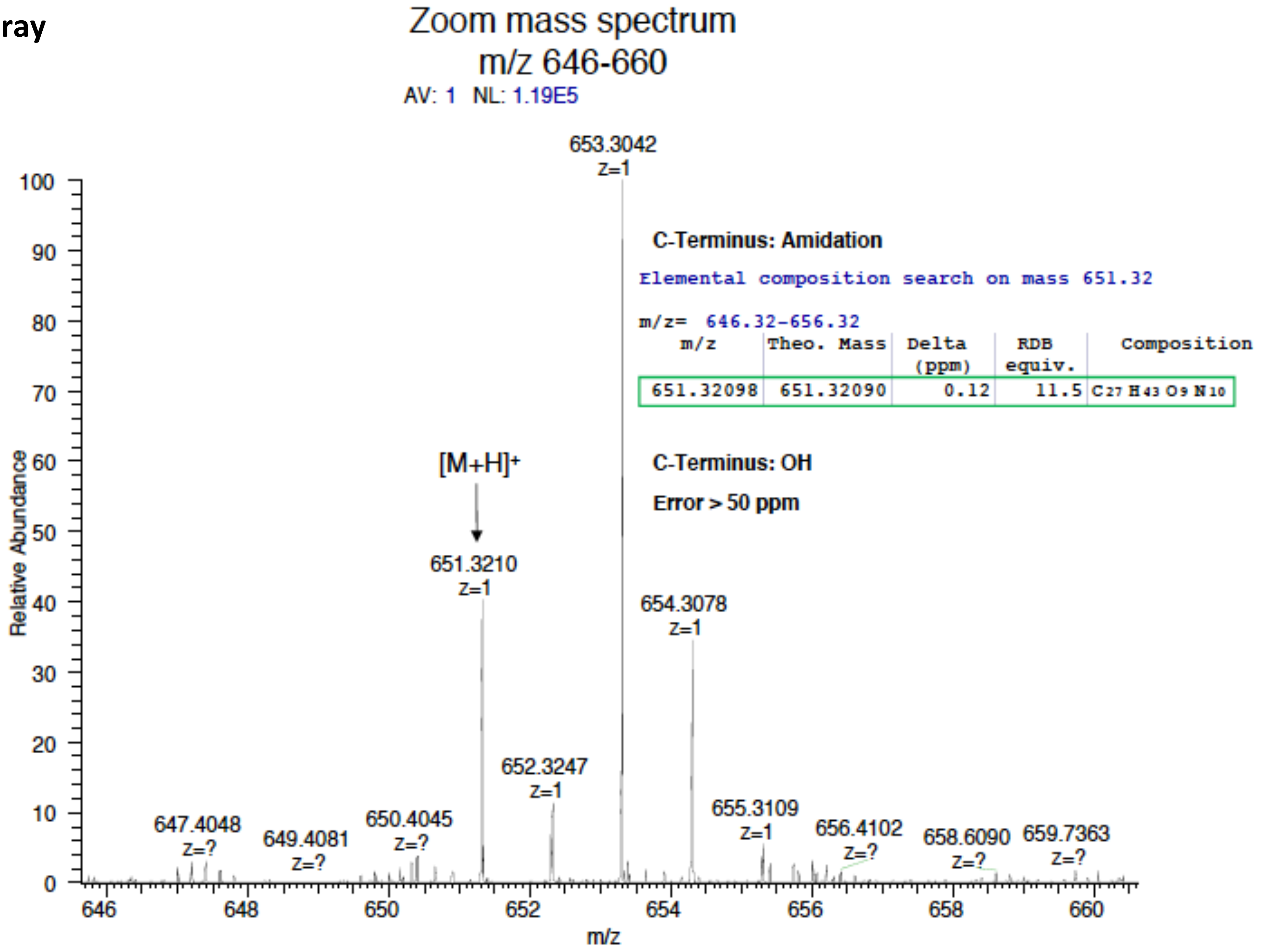

Zoom mass spectrum
m/z 646-660
AV: 1 NL: 1.19E5
653.3042
Z=1
C-Terminus: Amidation
Elemental composition search on mass 651.32
m/z= 646.32-656.32
m/z | Theo. Mass | Delta (ppm) | RDB equiv. | Composition
651.32098 | 651.32090 | 0.12 | 11.5 | C27 H43 O9 N10
C-Terminus: OH
Error > 50 ppm
[M+H]+
651.3210
Z=1
654.3078
Z=1
652.3247
Z=1
655.3109
Z=1
647.4048
Z=?
649.4081
Z=?
650.4045
Z=?
656.4102
Z=?
658.6090
Z=?
659.7363
Z=?
Relative Abundance
m/z

**wsGaay**

Zoom mass spectrum
m/z 646-660
AV: 1 NL: 1.19E5

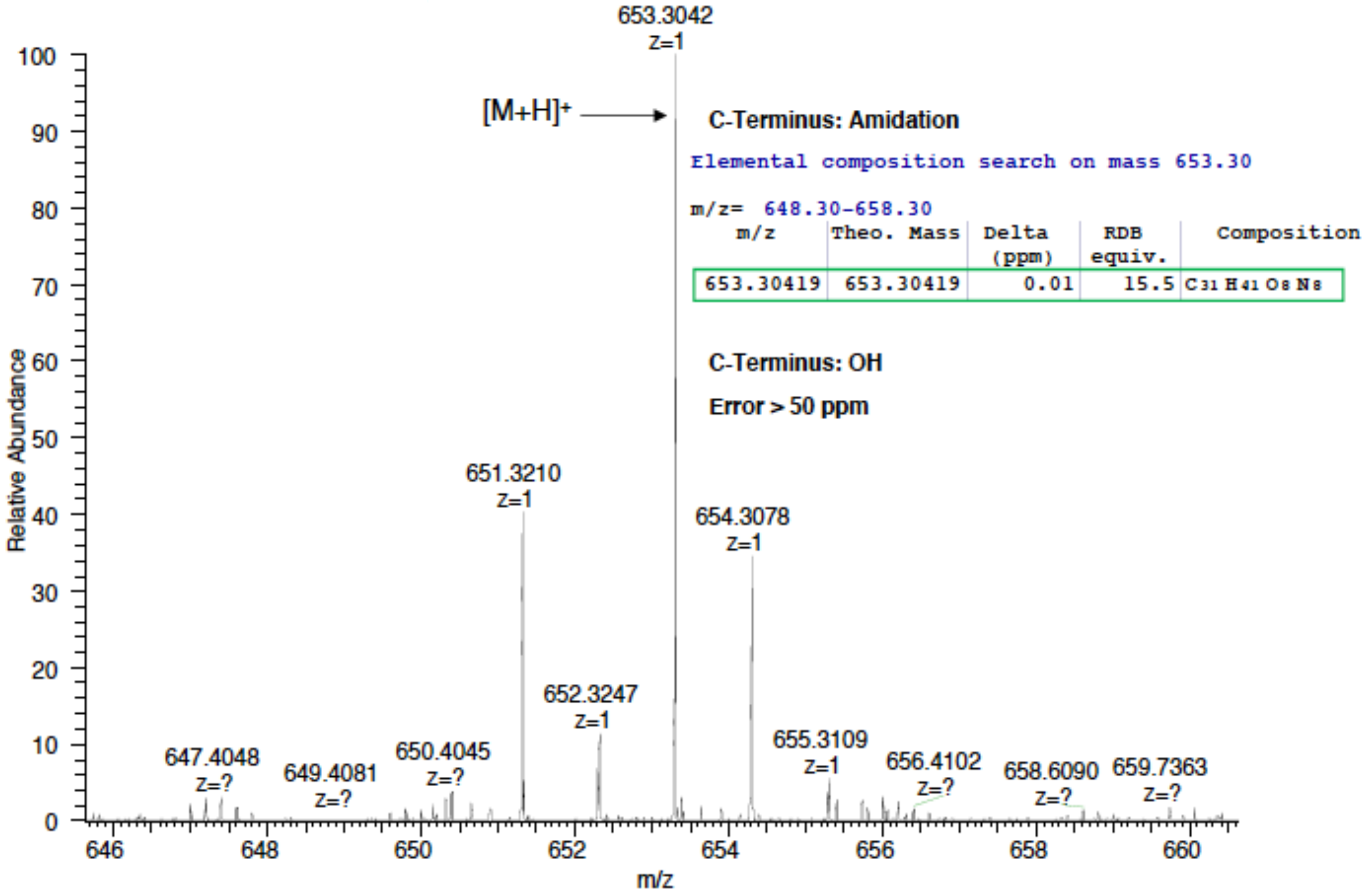

**Geraay**

**GeGrsy**

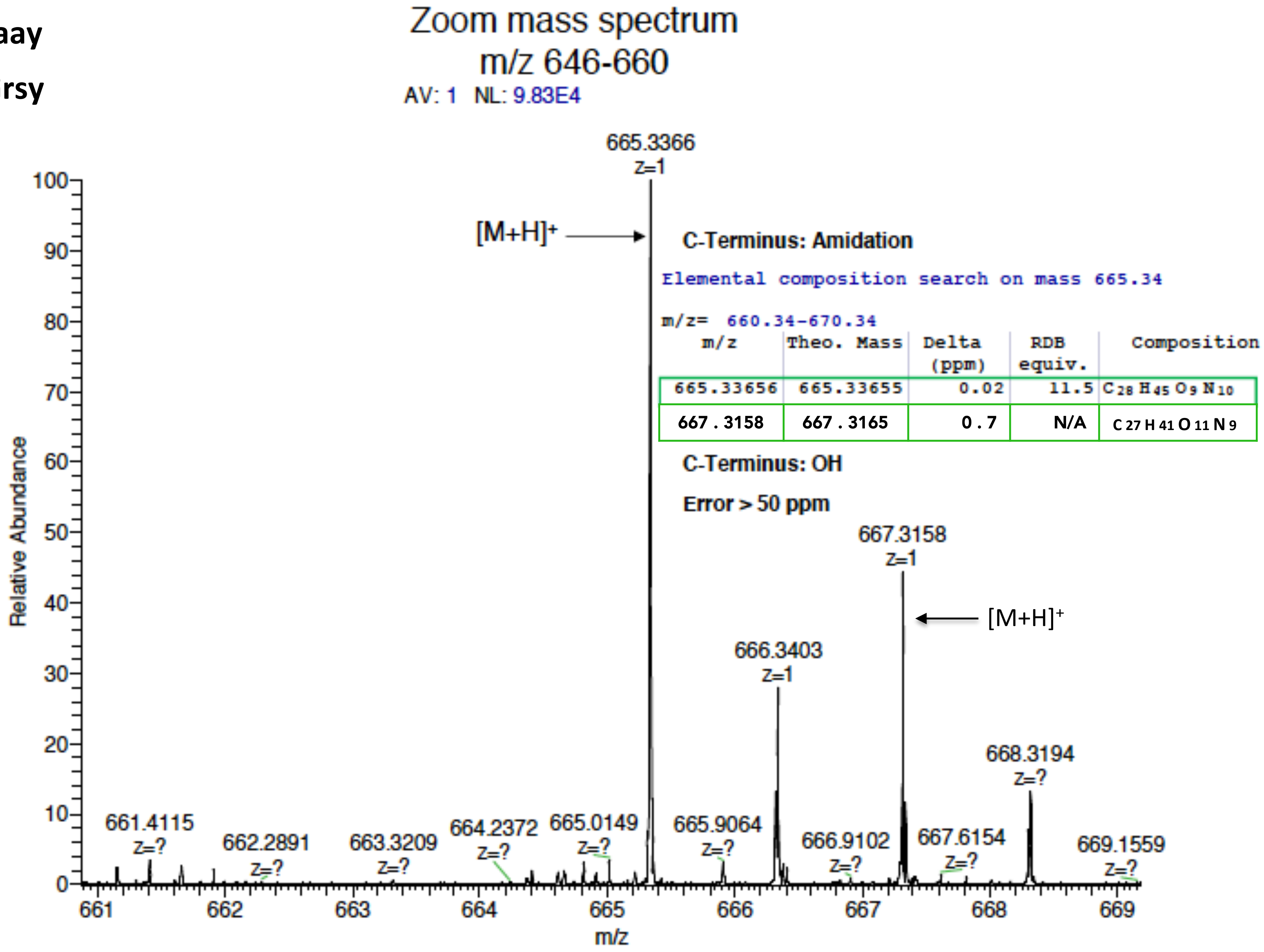


| m/z | Theo. Mass | Delta (ppm) | RDB equiv. | Composition |
|---|---|---|---|---|
| 665.33656 | 665.33655 | 0.02 | 11.5 | $C_{28}H_{45}O_9N_{10}$ |
| 667.3158 | 667.3165 | 0.7 | N/A | $C_{27}H_{41}O_{11}N_9$ |

**wsGasy**

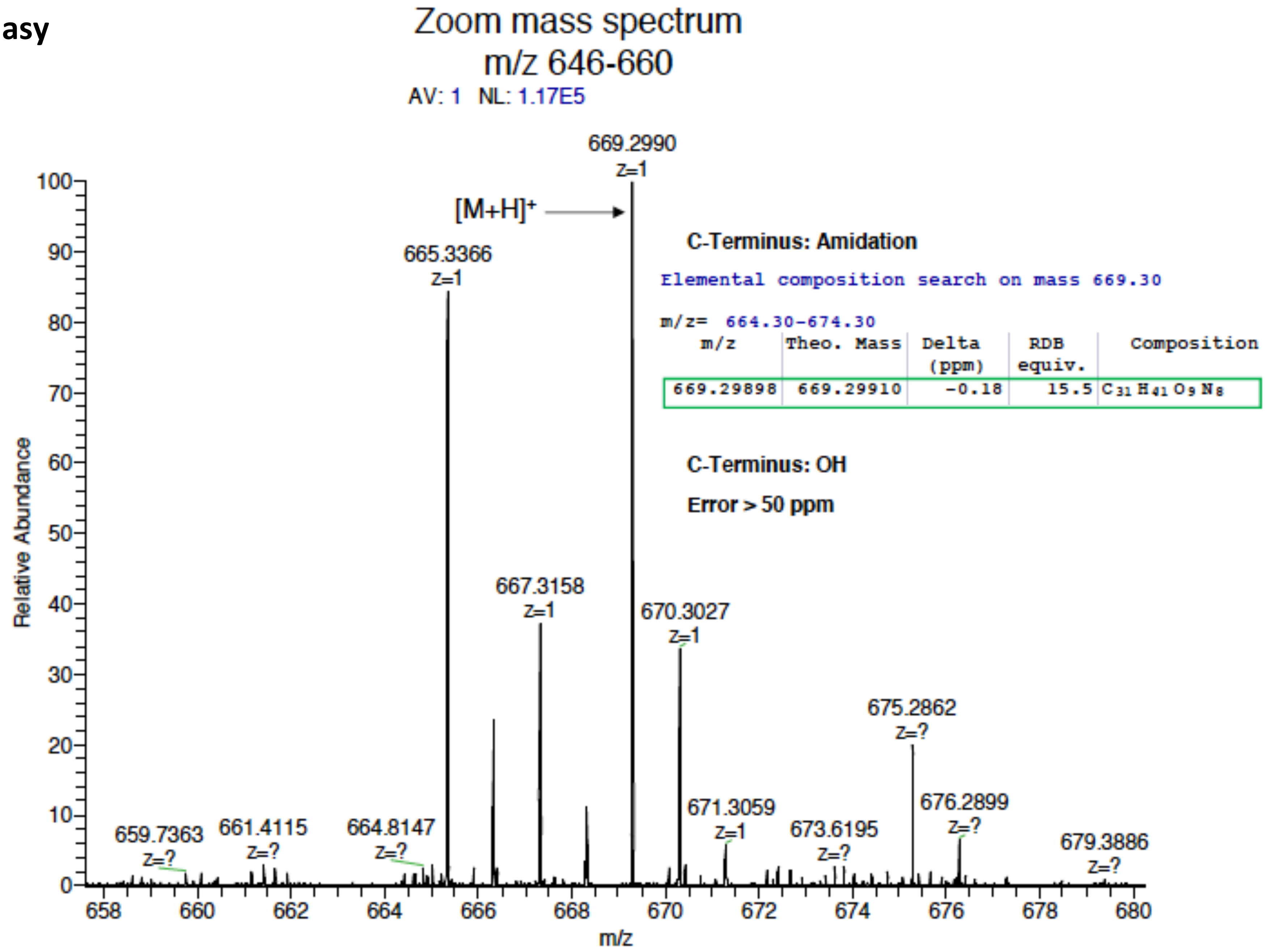

Zoom mass spectrum
m/z 646-660
AV: 1 NL: 1.17E5
[M+H]+
669.2990 z=1
665.3366 z=1
667.3158 z=1
670.3027 z=1
671.3059 z=1
675.2862 z=?
676.2899 z=?
659.7363 z=?
661.4115 z=?
664.8147 z=?
673.6195 z=?
679.3886 z=?
C-Terminus: Amidation
Elemental composition search on mass 669.30
m/z= 664.30-674.30
m/z | Theo. Mass | Delta (ppm) | RDB equiv. | Composition
669.29898 | 669.29910 | -0.18 | 15.5 | C31 H41 O9 N8
C-Terminus: OH
Error > 50 ppm
Relative Abundance
m/z

**Gerasy**

# Zoom mass spectrum
# m/z 646-660

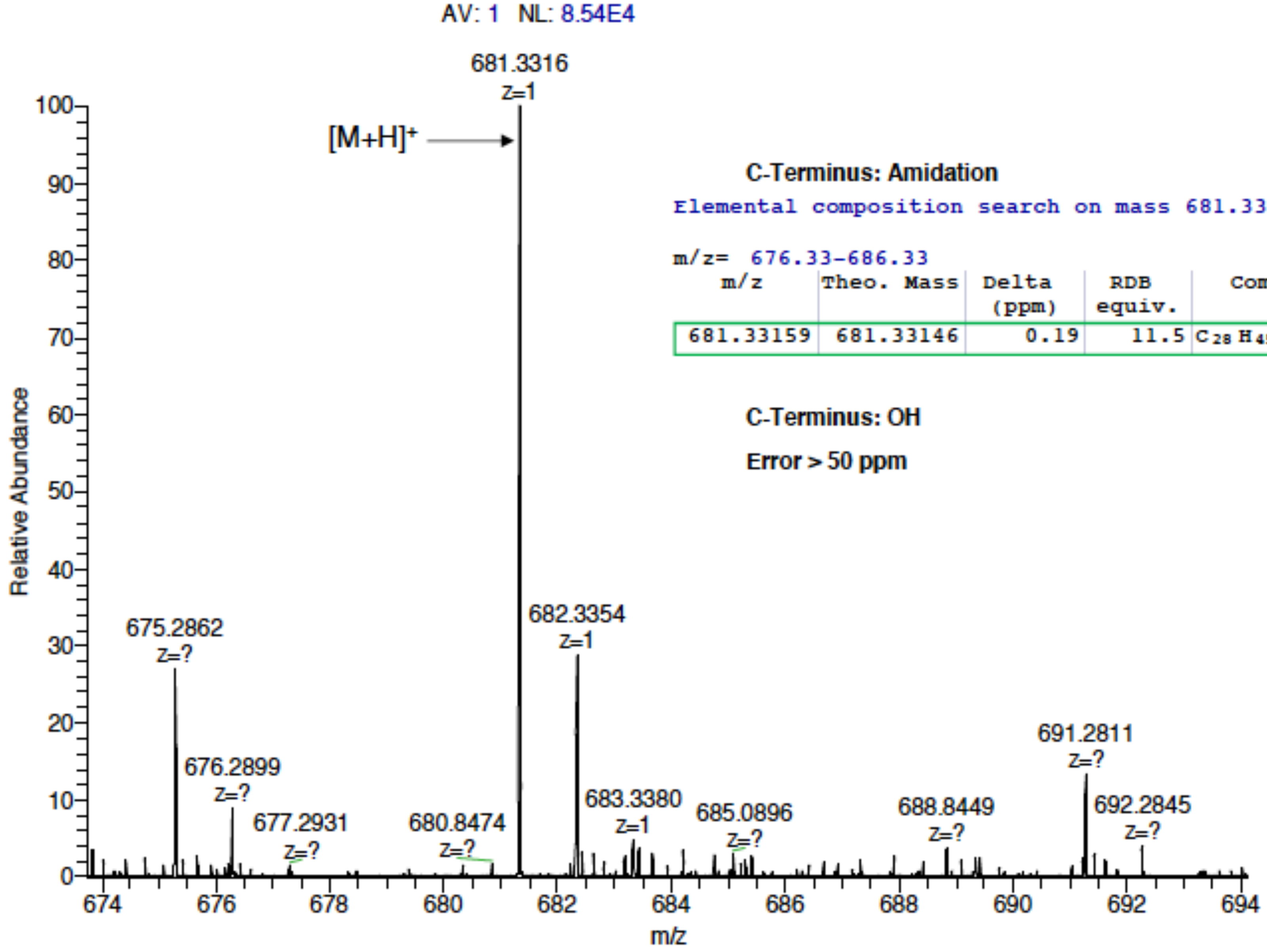


**C-Terminus: Amidation**

Elemental composition search on mass 681.33

m/z= 676.33-686.33

| m/z | Theo. Mass | Delta (ppm) | RDB equiv. | Composition |
|---|---|---|---|---|
| 681.33159 | 681.33146 | 0.19 | 11.5 | $C_{28} H_{45} O_{10} N_{10}$ |

**C-Terminus: OH**

**Error > 50 ppm**

**weGaay**

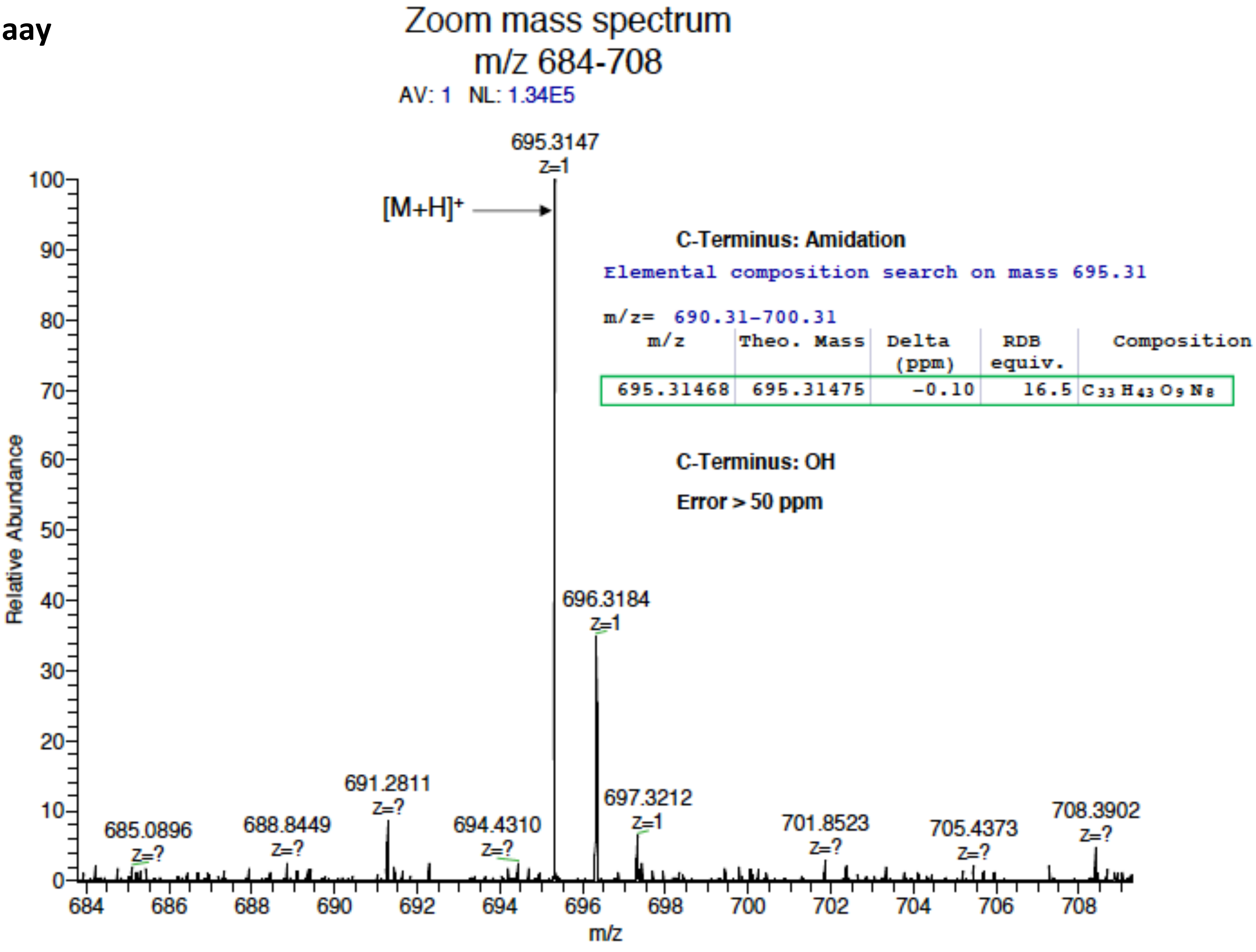

Zoom mass spectrum
m/z 684-708
AV: 1 NL: 1.34E5
695.3147
z=1
[M+H]+
C-Terminus: Amidation
Elemental composition search on mass 695.31
m/z= 690.31-700.31
m/z | Theo. Mass | Delta (ppm) | RDB equiv. | Composition
695.31468 | 695.31475 | -0.10 | 16.5 | C33 H43 O9 N8
C-Terminus: OH
Error > 50 ppm
696.3184
z=1
697.3212
z=1
685.0896
z=?
688.8449
z=?
691.2811
z=?
694.4310
z=?
701.8523
z=?
705.4373
z=?
708.3902
z=?
Relative Abundance
m/z

**Gsrray**

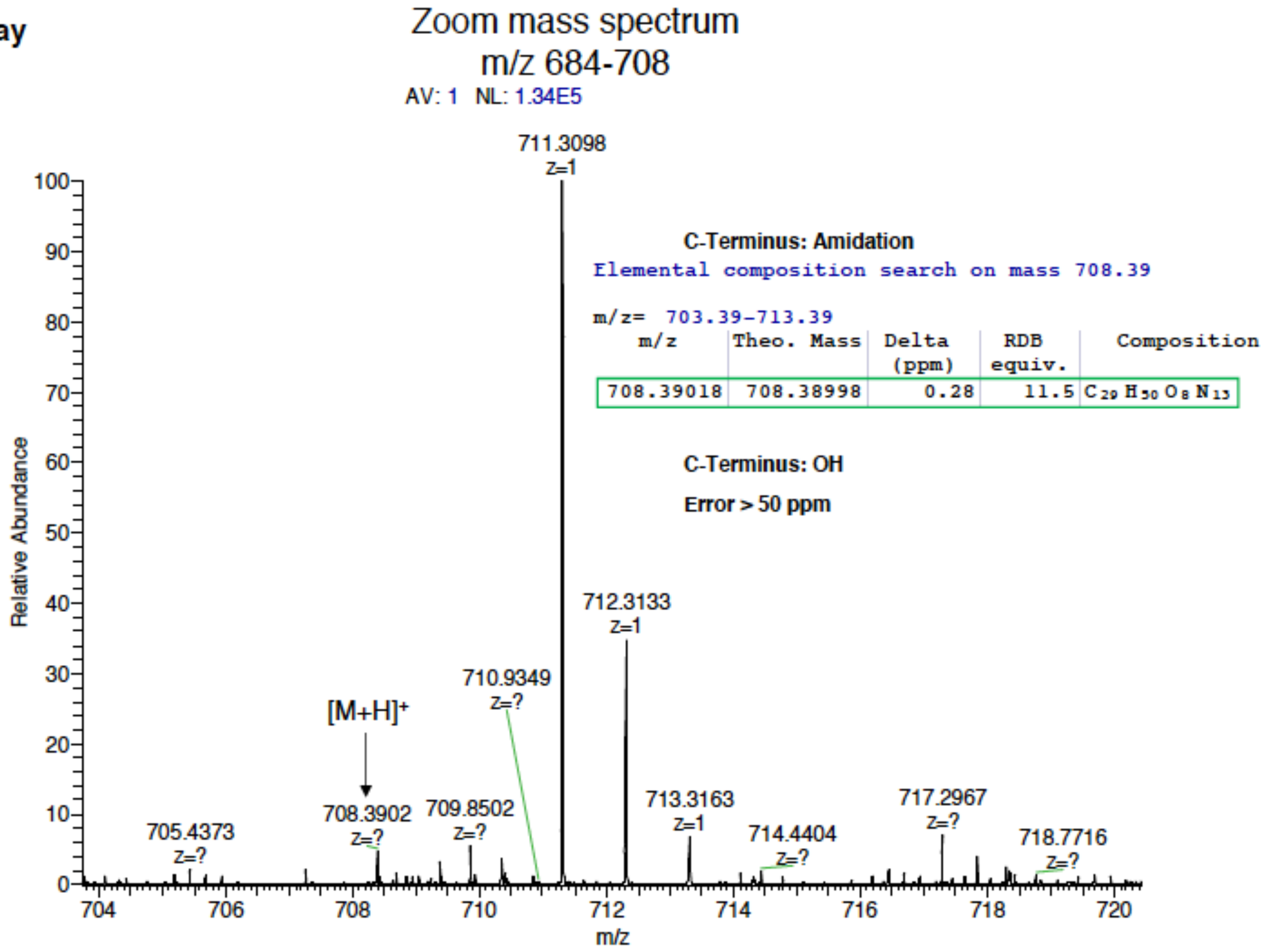
Zoom mass spectrum
m/z 684-708
AV: 1 NL: 1.34E5
711.3098
z=1
C-Terminus: Amidation
Elemental composition search on mass 708.39
m/z= 703.39-713.39
m/z | Theo. Mass | Delta (ppm) | RDB equiv. | Composition
708.39018 | 708.38998 | 0.28 | 11.5 | C29 H50 O8 N13
C-Terminus: OH
Error > 50 ppm
712.3133
z=1
710.9349
z=?
[M+H]+
708.3902
z=?
709.8502
z=?
705.4373
z=?
713.3163
z=1
714.4404
z=?
717.2967
z=?
718.7716
z=?
Relative Abundance
m/z

**weGasy**

# Zoom mass spectrum
## m/z 698-735

AV: 1 NL: 1.34E5

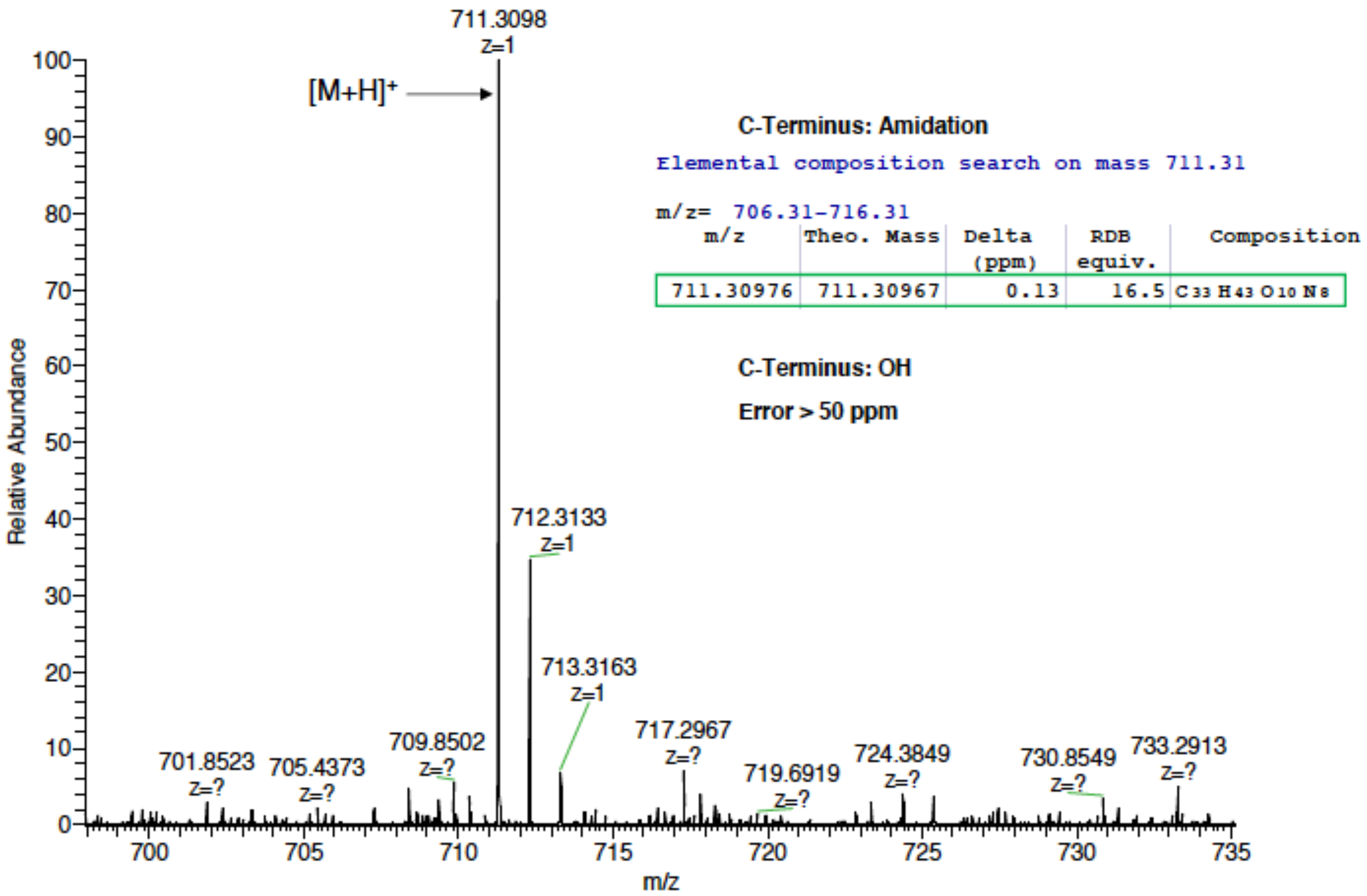


| m/z | Theo. Mass | Delta (ppm) | RDB equiv. | Composition |
|---|---|---|---|---|
| 711.30976 | 711.30967 | 0.13 | 16.5 | $C_{33}H_{43}O_{10}N_8$ |

**Gsrrsy**

Zoom mass spectrum
m/z 719-745

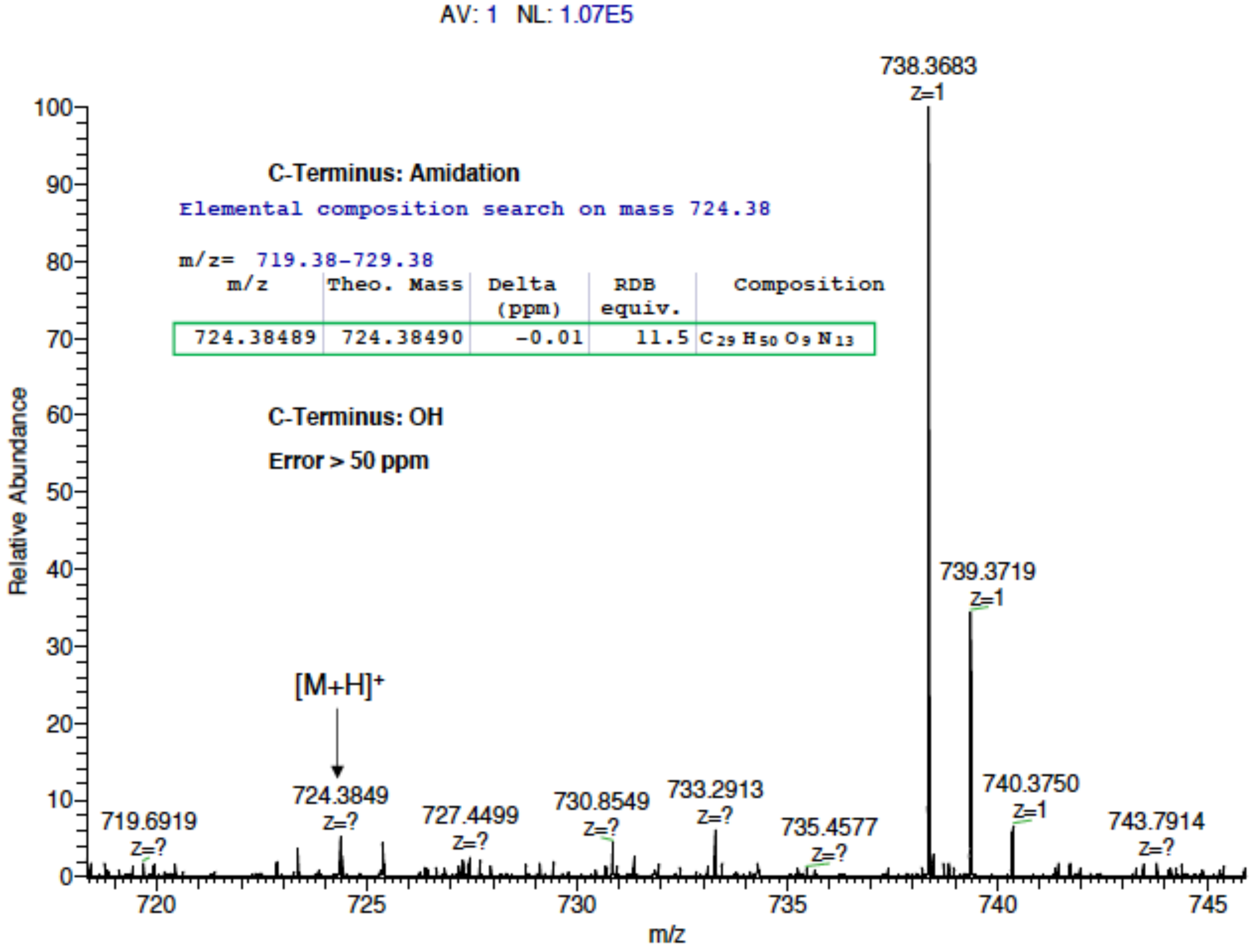

**wsGray**

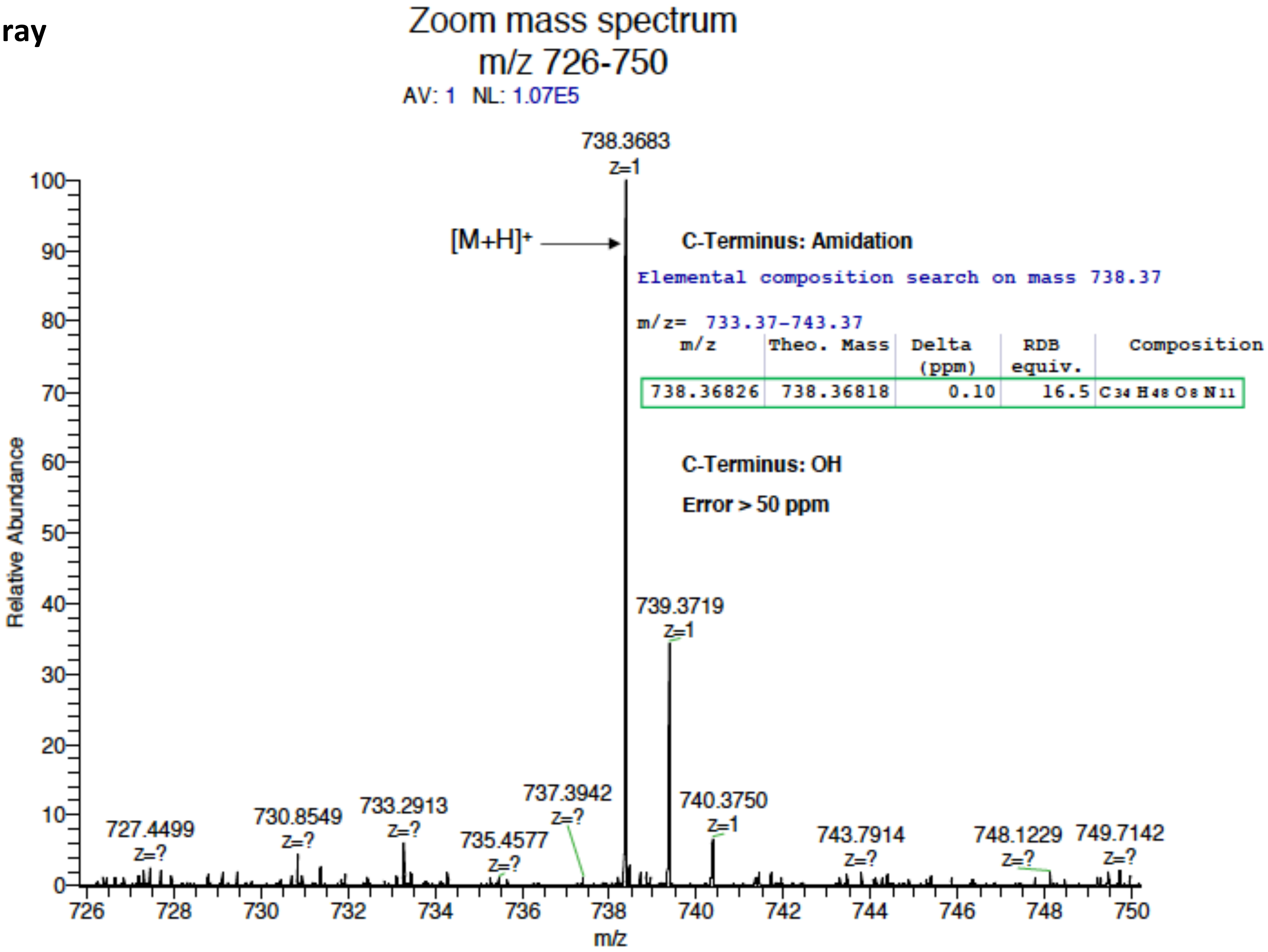


| m/z | Theo. Mass | Delta (ppm) | RDB equiv. | Composition |
|---|---|---|---|---|
| 738.36826 | 738.36818 | 0.10 | 16.5 | $C_{34} H_{48} O_8 N_{11}$ |

**Gerray**

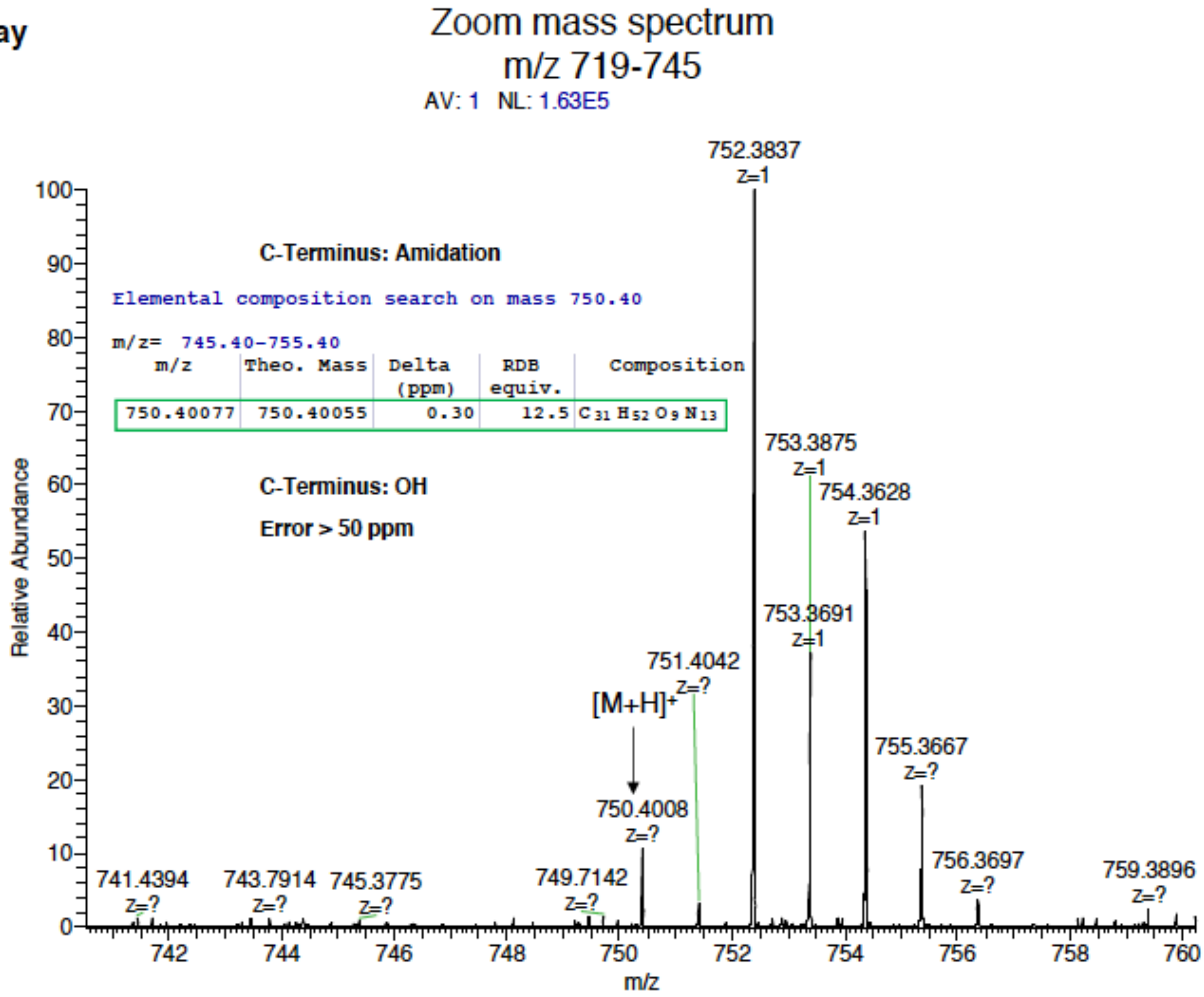
Zoom mass spectrum
m/z 719-745
AV: 1 NL: 1.63E5
C-Terminus: Amidation
Elemental composition search on mass 750.40
m/z= 745.40-755.40
m/z | Theo. Mass | Delta (ppm) | RDB equiv. | Composition
750.40077 | 750.40055 | 0.30 | 12.5 | C31 H52 O9 N13
C-Terminus: OH
Error > 50 ppm
[M+H]+
752.3837 z=1
753.3875 z=1
754.3628 z=1
753.3691 z=1
751.4042 z=?
755.3667 z=?
750.4008 z=?
756.3697 z=?
759.3896 z=?
741.4394 z=?
743.7914 z=?
745.3775 z=?
749.7142 z=?
Relative Abundance
m/z

**wsraay**

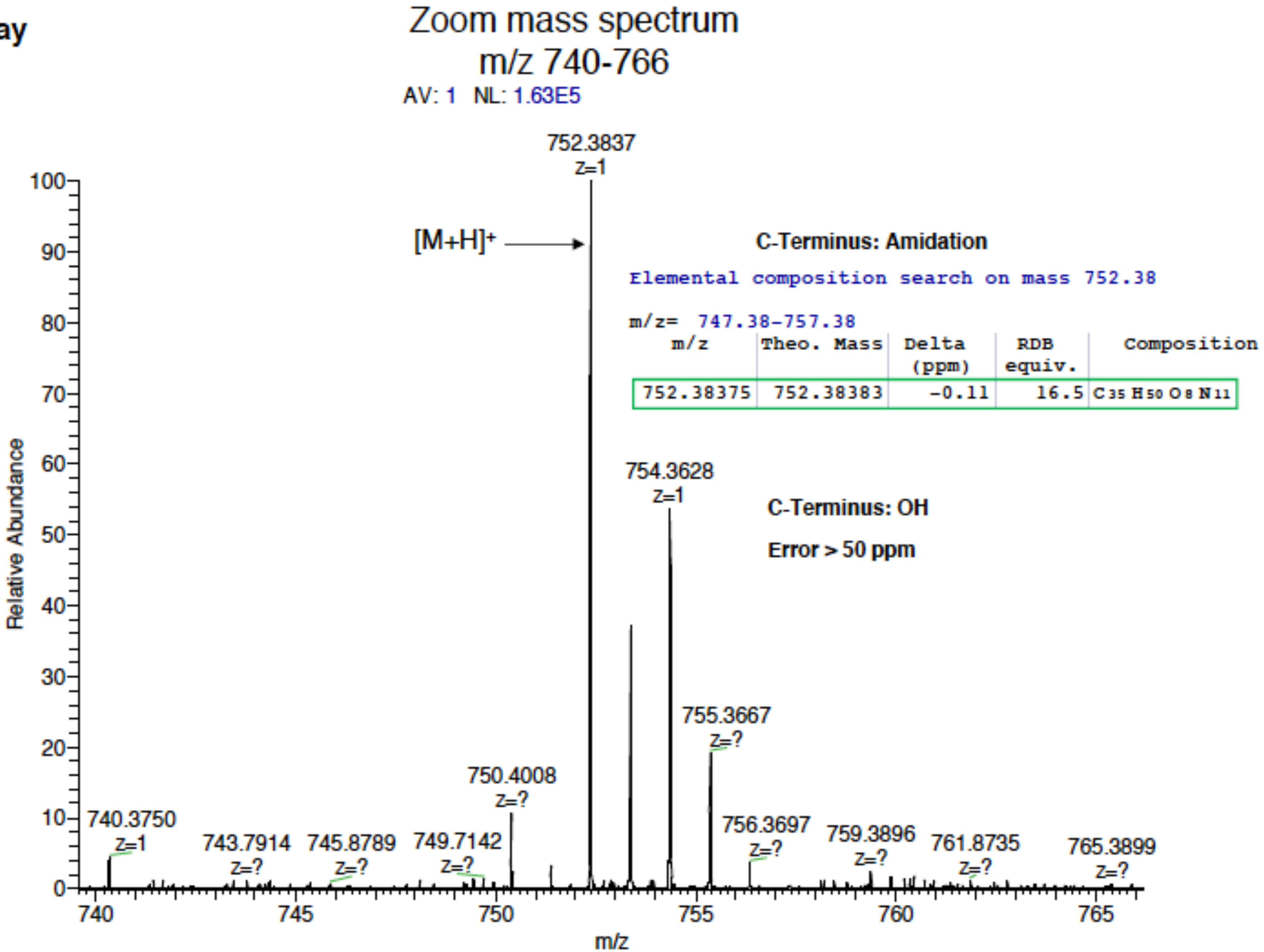
Zoom mass spectrum
m/z 740-766
AV: 1 NL: 1.63E5
752.3837
z=1
[M+H]+
C-Terminus: Amidation
Elemental composition search on mass 752.38
m/z= 747.38-757.38
m/z
Theo. Mass
Delta (ppm)
RDB equiv.
Composition
752.38375
752.38383
-0.11
16.5
C35 H50 O8 N11
754.3628
z=1
C-Terminus: OH
Error > 50 ppm
755.3667
z=?
750.4008
z=?
740.3750
z=1
743.7914
z=?
745.8789
z=?
749.7142
z=?
756.3697
z=?
759.3896
z=?
761.8735
z=?
765.3899
z=?
Relative Abundance
m/z
100
90
80
70
60
50
40
30
20
10
0
740
745
750
755
760
765

**wsGrsy**

# Zoom mass spectrum
# m/z 748-766

AV: 1 NL: 1.63E5

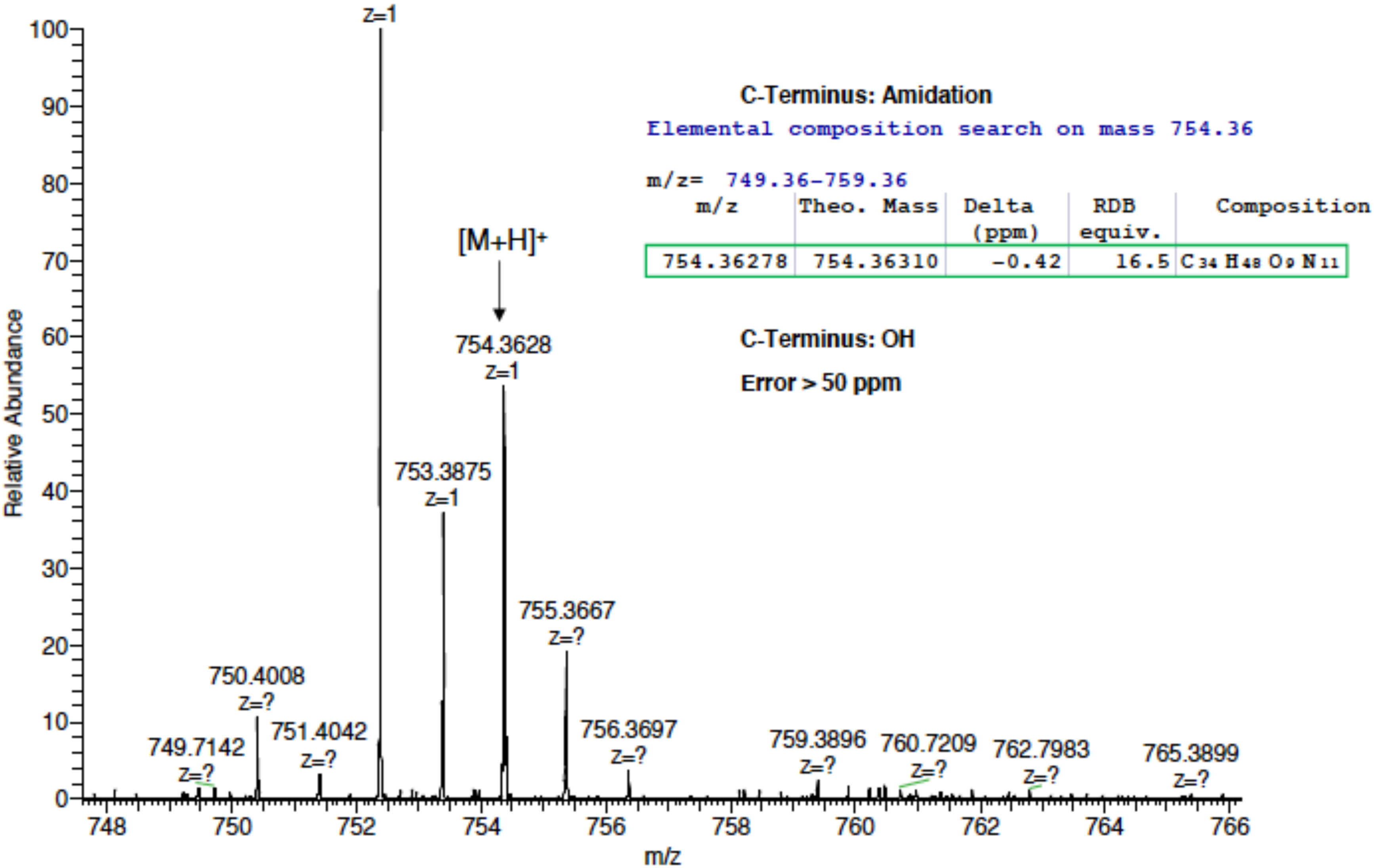


**C-Terminus: Amidation**

Elemental composition search on mass 754.36

m/z= 749.36-759.36

| m/z | Theo. Mass | Delta (ppm) | RDB equiv. | Composition |
|---|---|---|---|---|
| 754.36278 | 754.36310 | -0.42 | 16.5 | $C_{34}H_{48}O_{9}N_{11}$ |

**C-Terminus: OH**

**Error > 50 ppm**

**Gerrsy**

# Zoom mass spectrum
# m/z 760-779

AV: 1 NL: 1.27E5

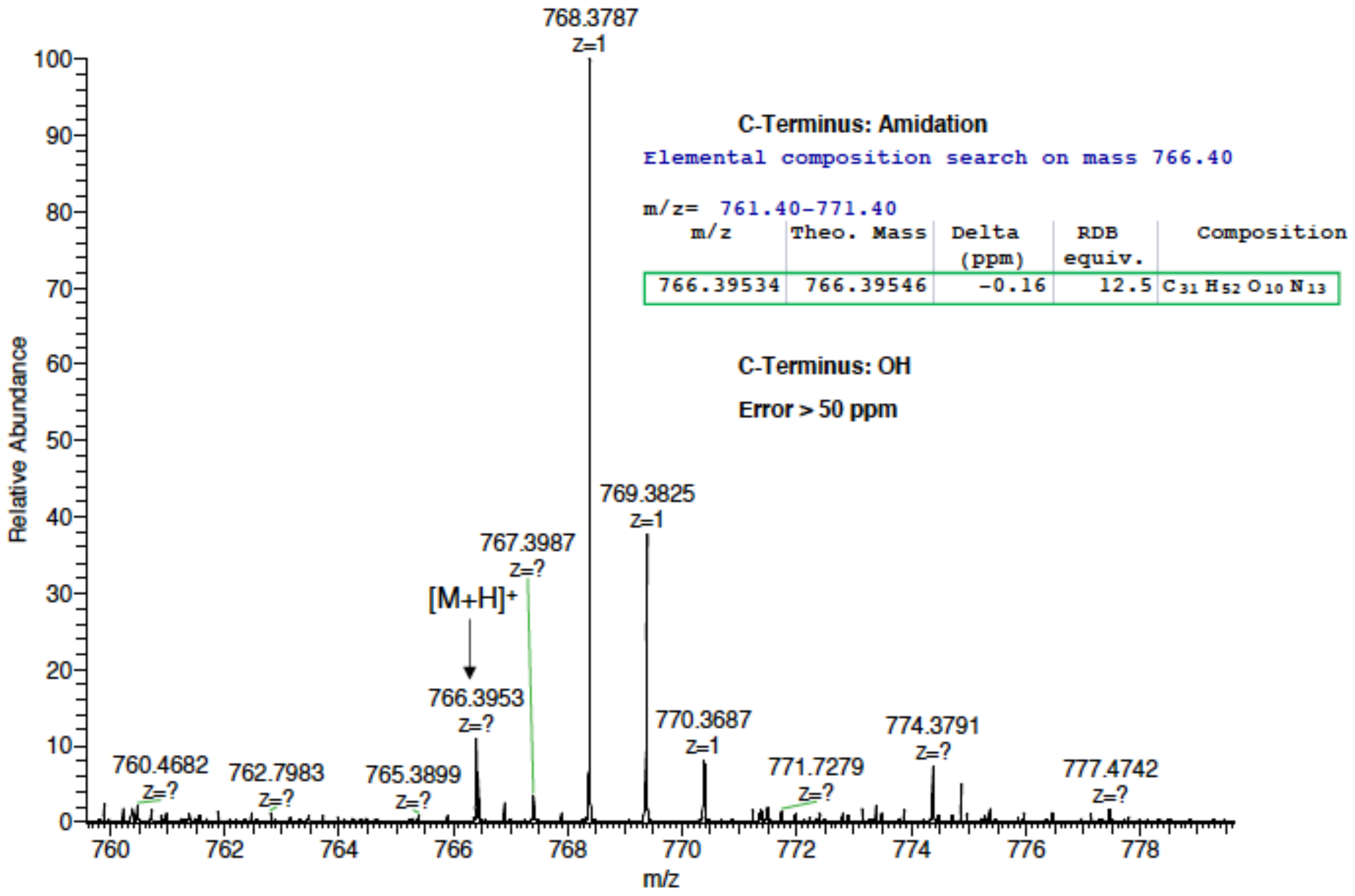

**wsrasy**

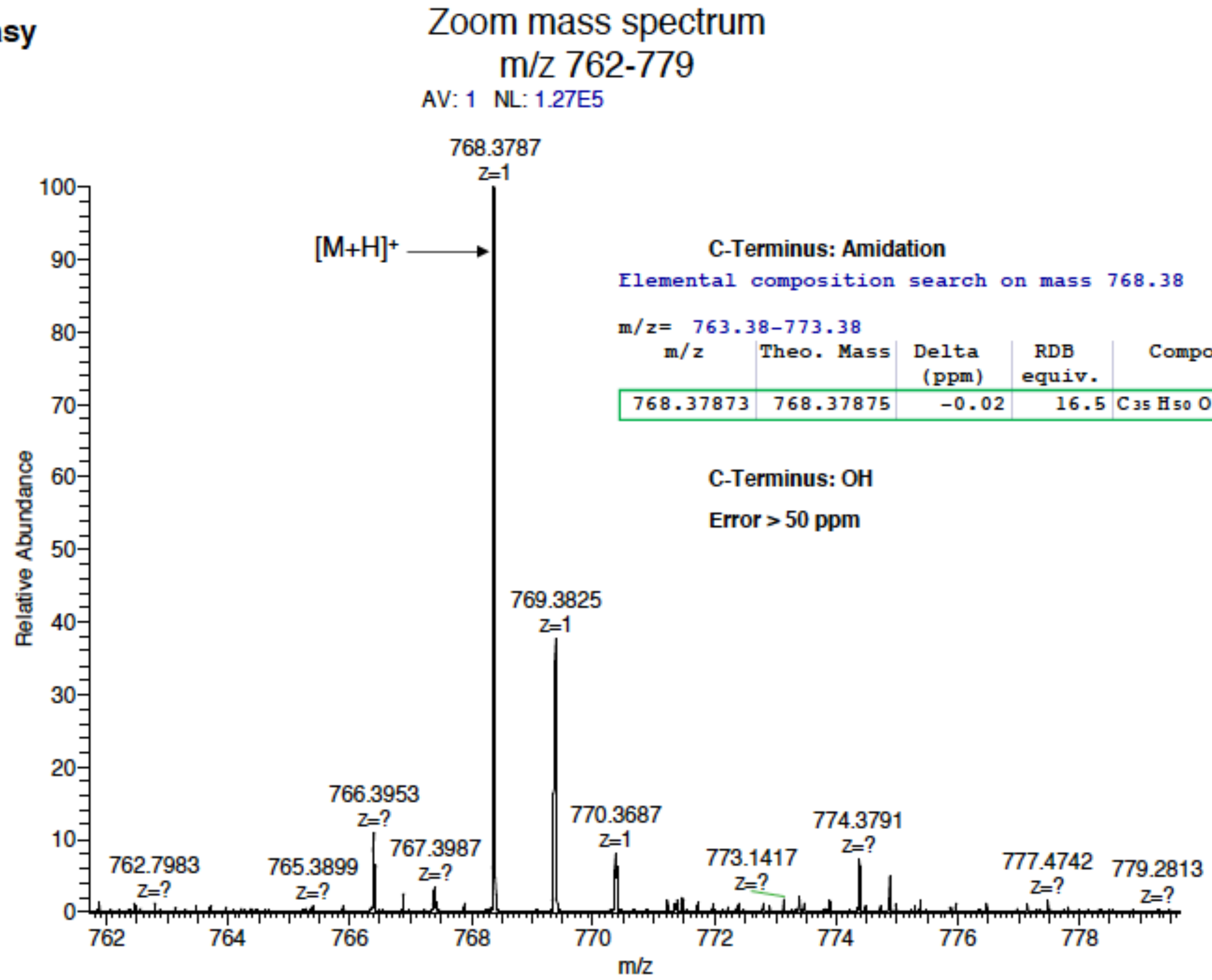


**C-Terminus: Amidation**

Elemental composition search on mass 768.38

m/z= 763.38-773.38

| m/z | Theo. Mass | Delta (ppm) | RDB equiv. | Composition |
|---|---|---|---|---|
| 768.37873 | 768.37875 | -0.02 | 16.5 | $C_{35} H_{50} O_9 N_{11}$ |

**C-Terminus: OH**

**Error > 50 ppm**

**weGray**

## Zoom mass spectrum
## m/z 771-792

AV: 1 NL: 9.71E4

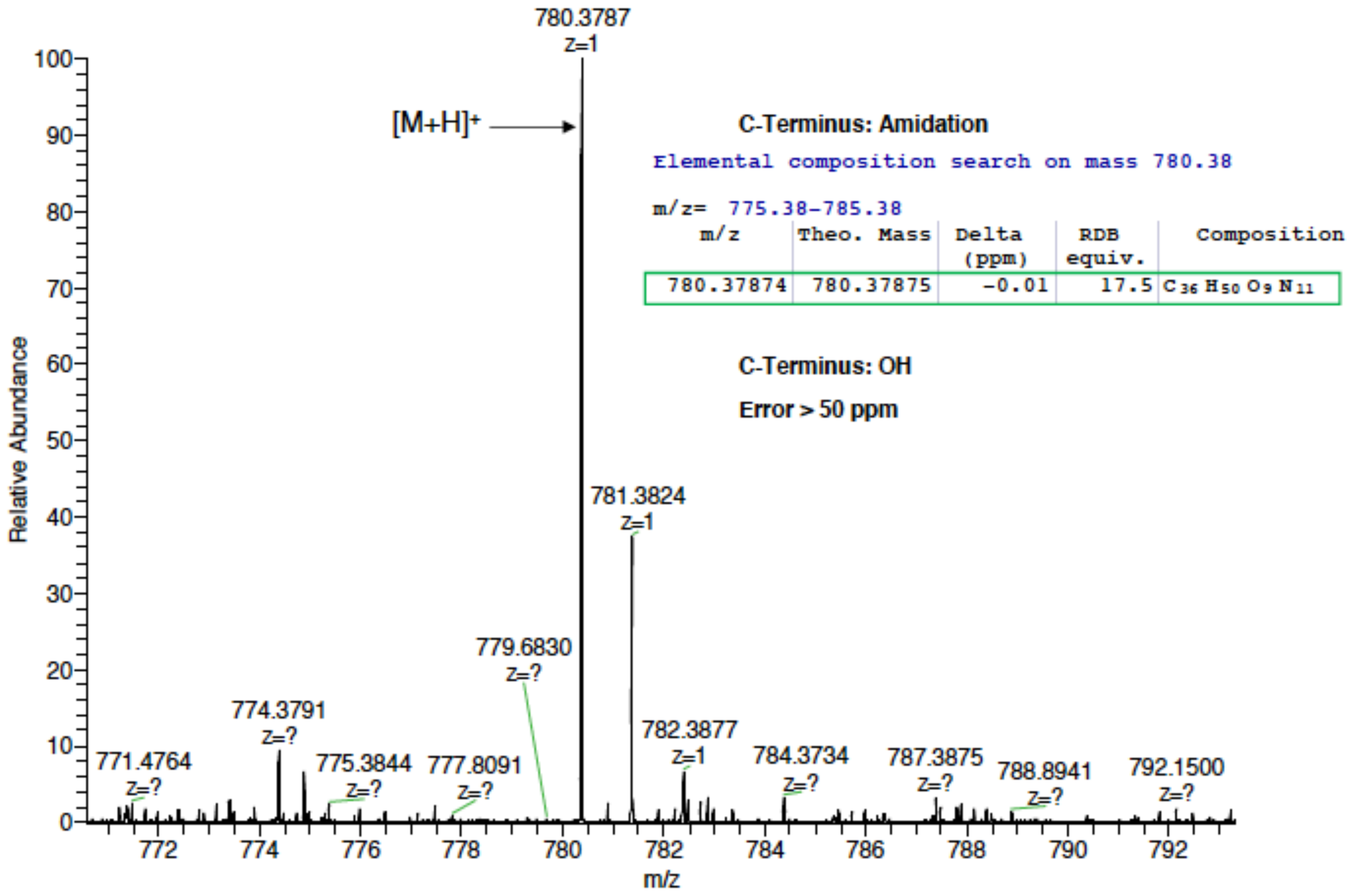


| m/z | Theo. Mass | Delta (ppm) | RDB equiv. | Composition |
|---|---|---|---|---|
| 780.37874 | 780.37875 | -0.01 | 17.5 | $C_{36}H_{50}O_{9}N_{11}$ |

**weraay**

## Zoom mass spectrum
## m/z 784-806

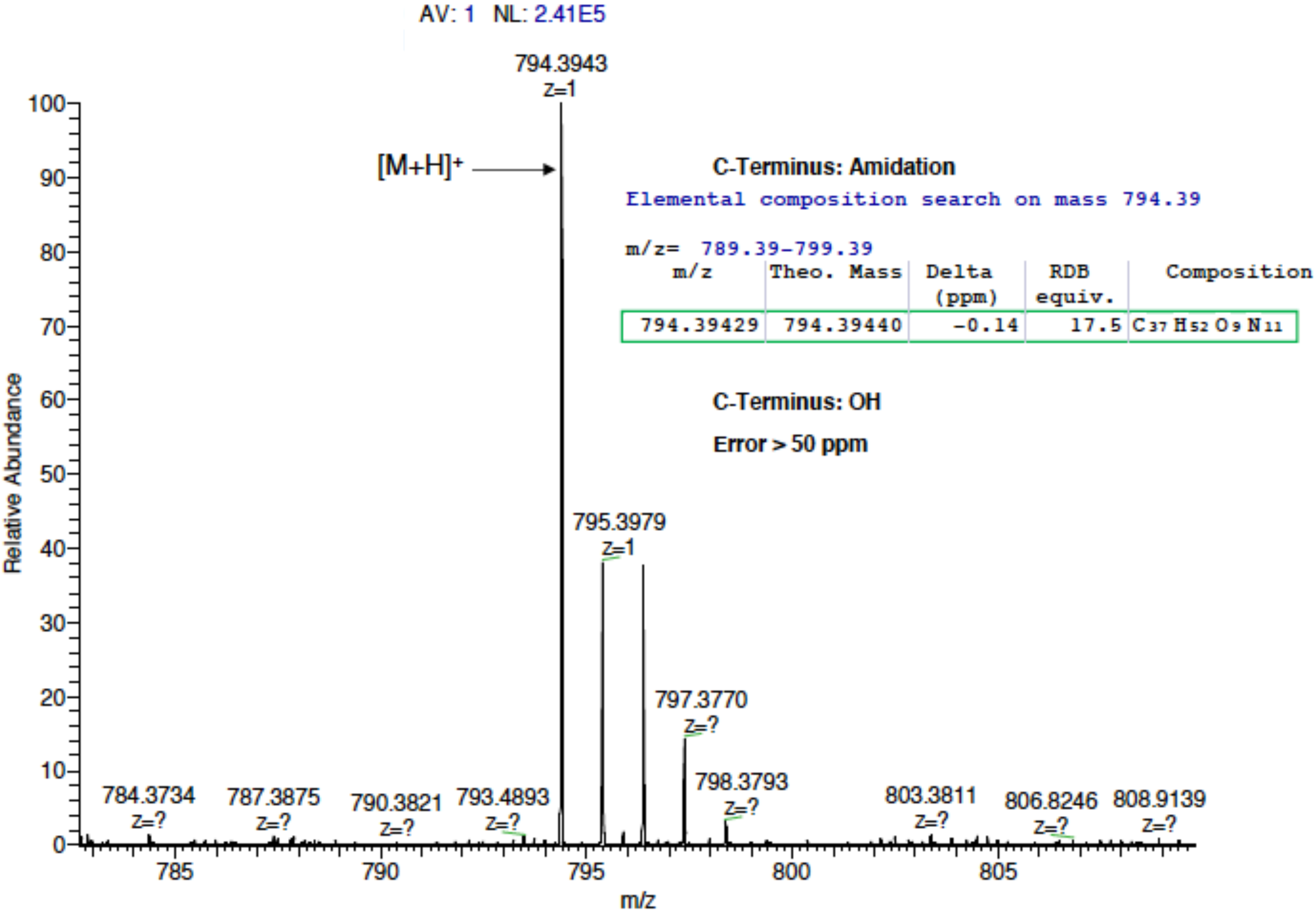


| m/z | Theo. Mass | Delta (ppm) | RDB equiv. | Composition |
|---|---|---|---|---|
| 794.39429 | 794.39440 | -0.14 | 17.5 | $C_{37}H_{52}O_9N_{11}$ |

**weGrsy**

Zoom mass spectrum
m/z 792-806
AV: 1 NL: 2.41E5

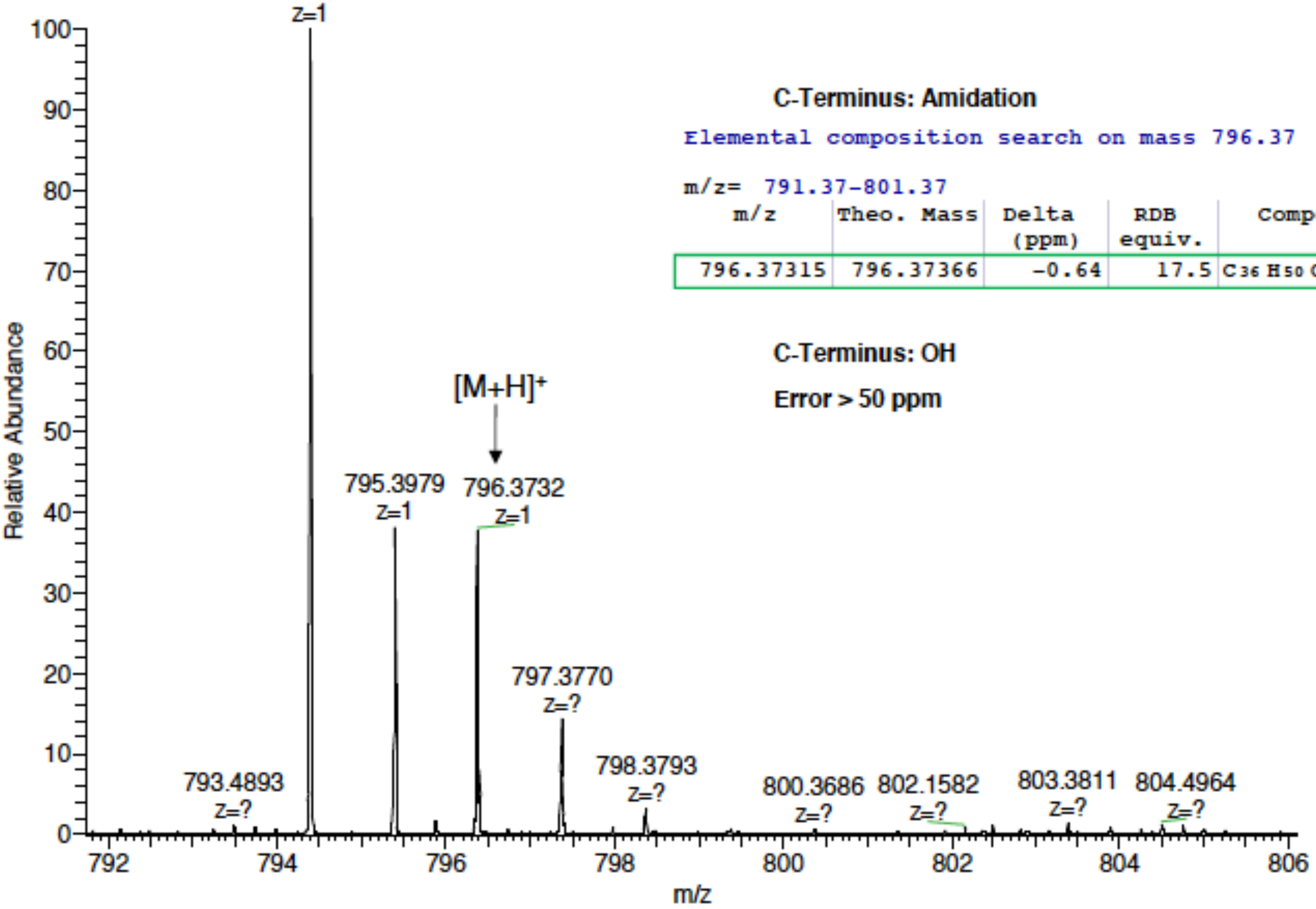


**C-Terminus: Amidation**

Elemental composition search on mass 796.37

m/z= 791.37-801.37

| m/z | Theo. Mass | Delta (ppm) | RDB equiv. | Composition |
|---|---|---|---|---|
| 796.37315 | 796.37366 | -0.64 | 17.5 | $C_{36}H_{50}O_{10}N_{11}$ |

**C-Terminus: OH**

**Error > 50 ppm**

**werasy**

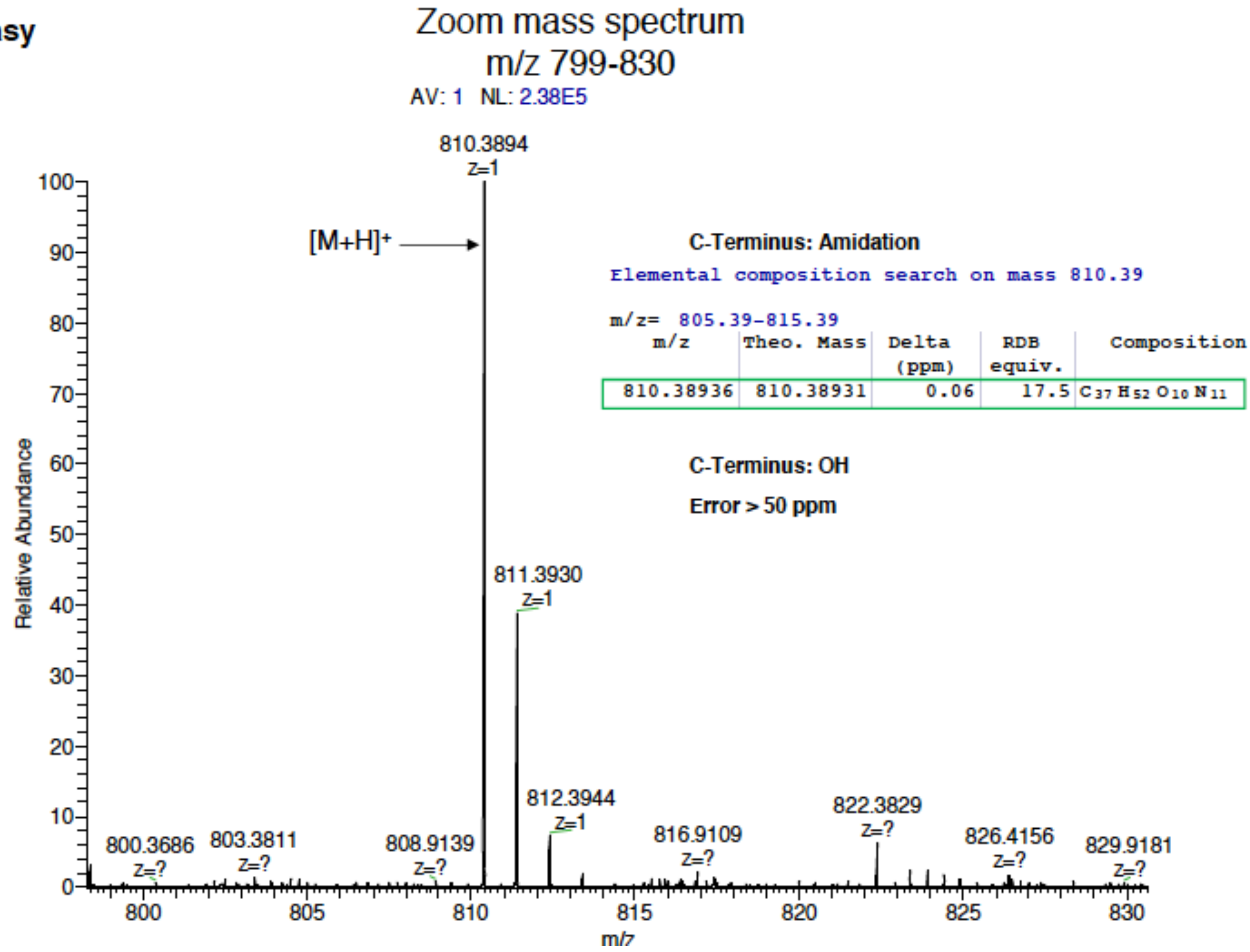

Zoom mass spectrum
m/z 799-830
AV: 1 NL: 2.38E5
810.3894
z=1
[M+H]+
C-Terminus: Amidation
Elemental composition search on mass 810.39
m/z= 805.39-815.39
m/z | Theo. Mass | Delta (ppm) | RDB equiv. | Composition
810.38936 | 810.38931 | 0.06 | 17.5 | C37 H52 O10 N11
C-Terminus: OH
Error > 50 ppm
811.3930
z=1
812.3944
z=1
800.3686
z=?
803.3811
z=?
808.9139
z=?
816.9109
z=?
822.3829
z=?
826.4156
z=?
829.9181
z=?
Relative Abundance
m/z

**wsrray**

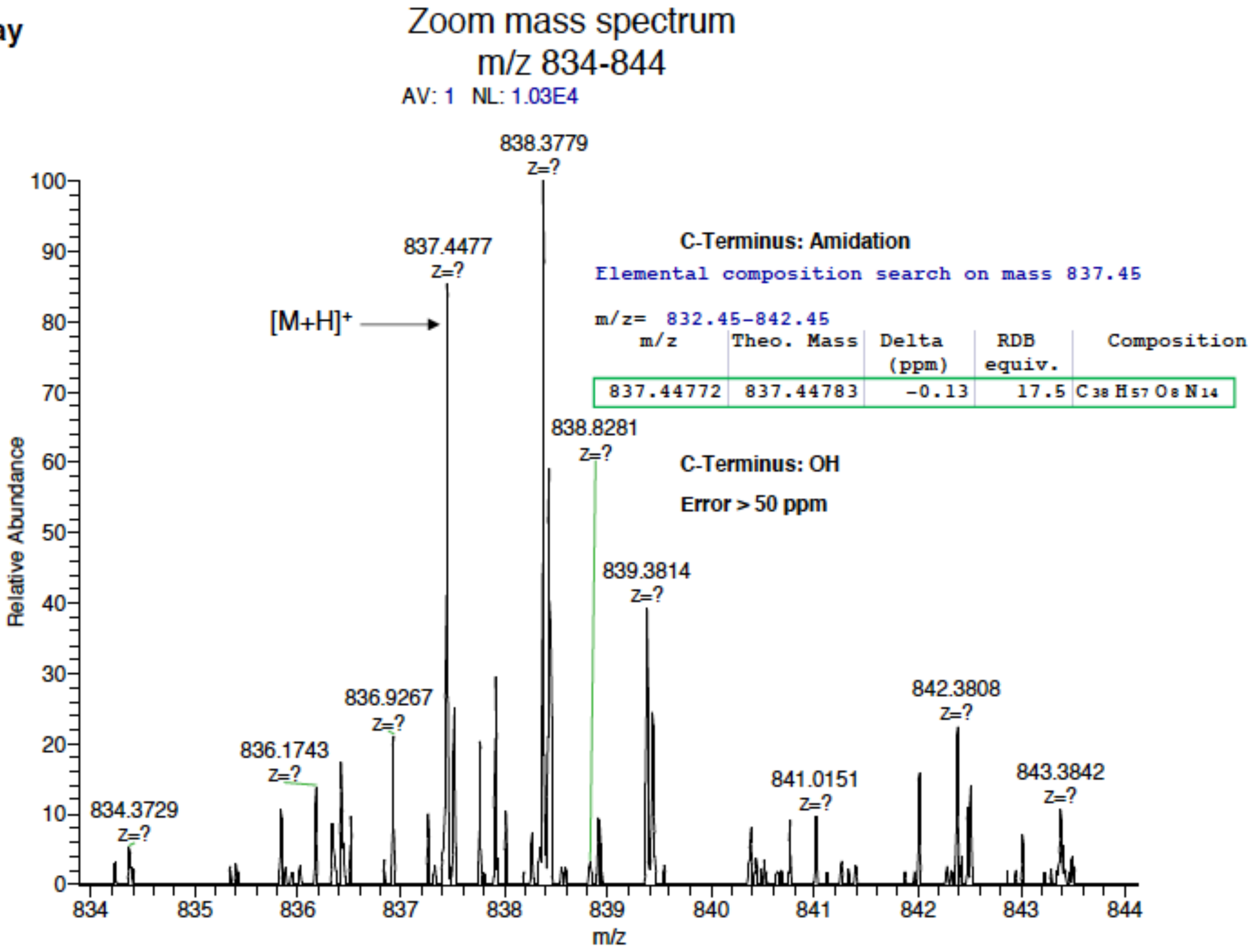
Zoom mass spectrum
m/z 834-844
AV: 1 NL: 1.03E4
838.3779 z=?
837.4477 z=?
[M+H]+
838.8281 z=?
839.3814 z=?
836.9267 z=?
836.1743 z=?
834.3729 z=?
841.0151 z=?
842.3808 z=?
843.3842 z=?
C-Terminus: Amidation
Elemental composition search on mass 837.45
m/z= 832.45-842.45
m/z | Theo. Mass | Delta (ppm) | RDB equiv. | Composition
837.44772 | 837.44783 | -0.13 | 17.5 | C38 H57 O8 N14
C-Terminus: OH
Error > 50 ppm
Relative Abundance
m/z

**wsrrsy**

Zoom mass spectrum
m/z 849-861

AV: 1 NL: 7.32E3

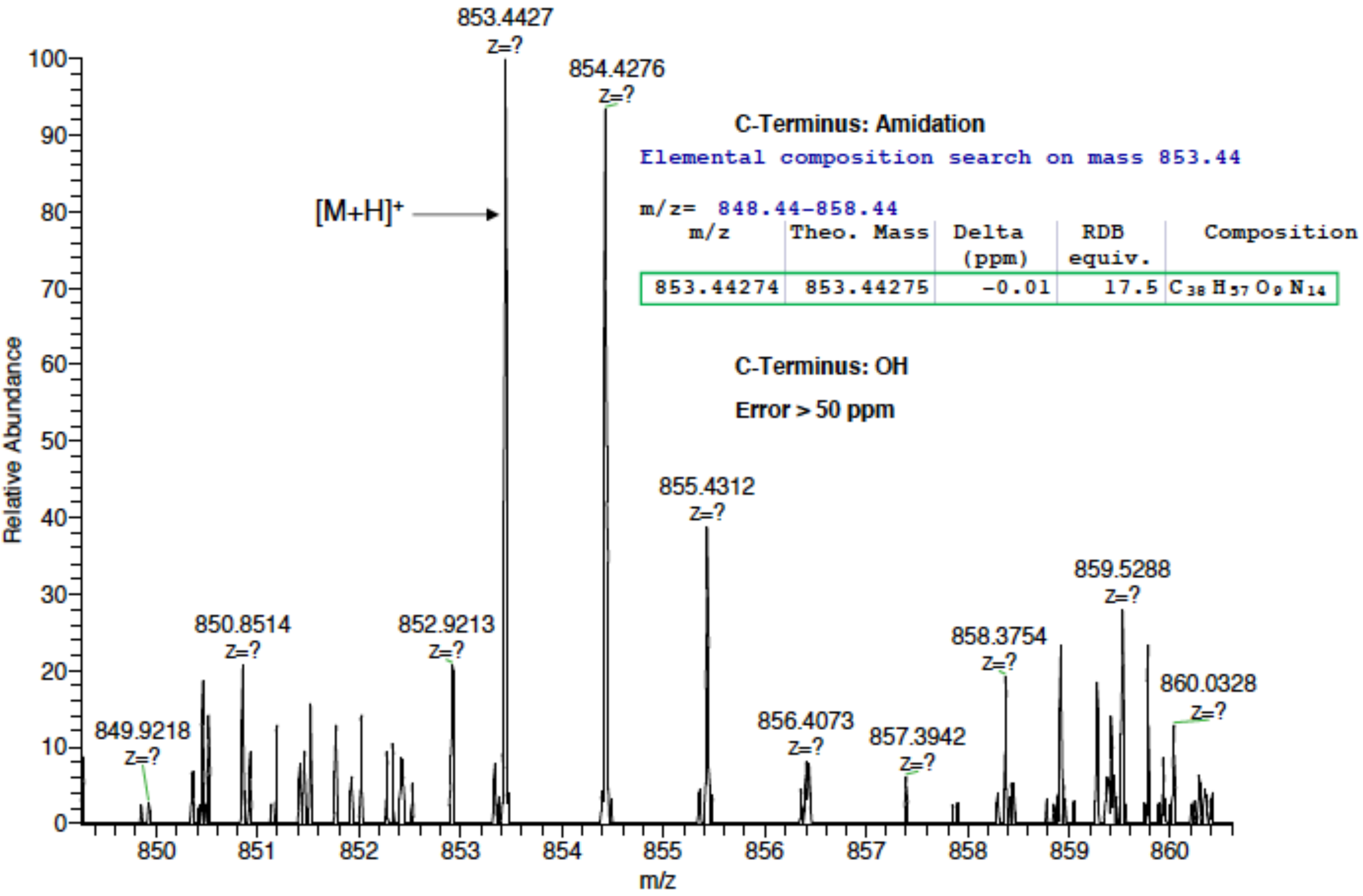

**werray**

## Zoom mass spectrum
## m/z 866-895

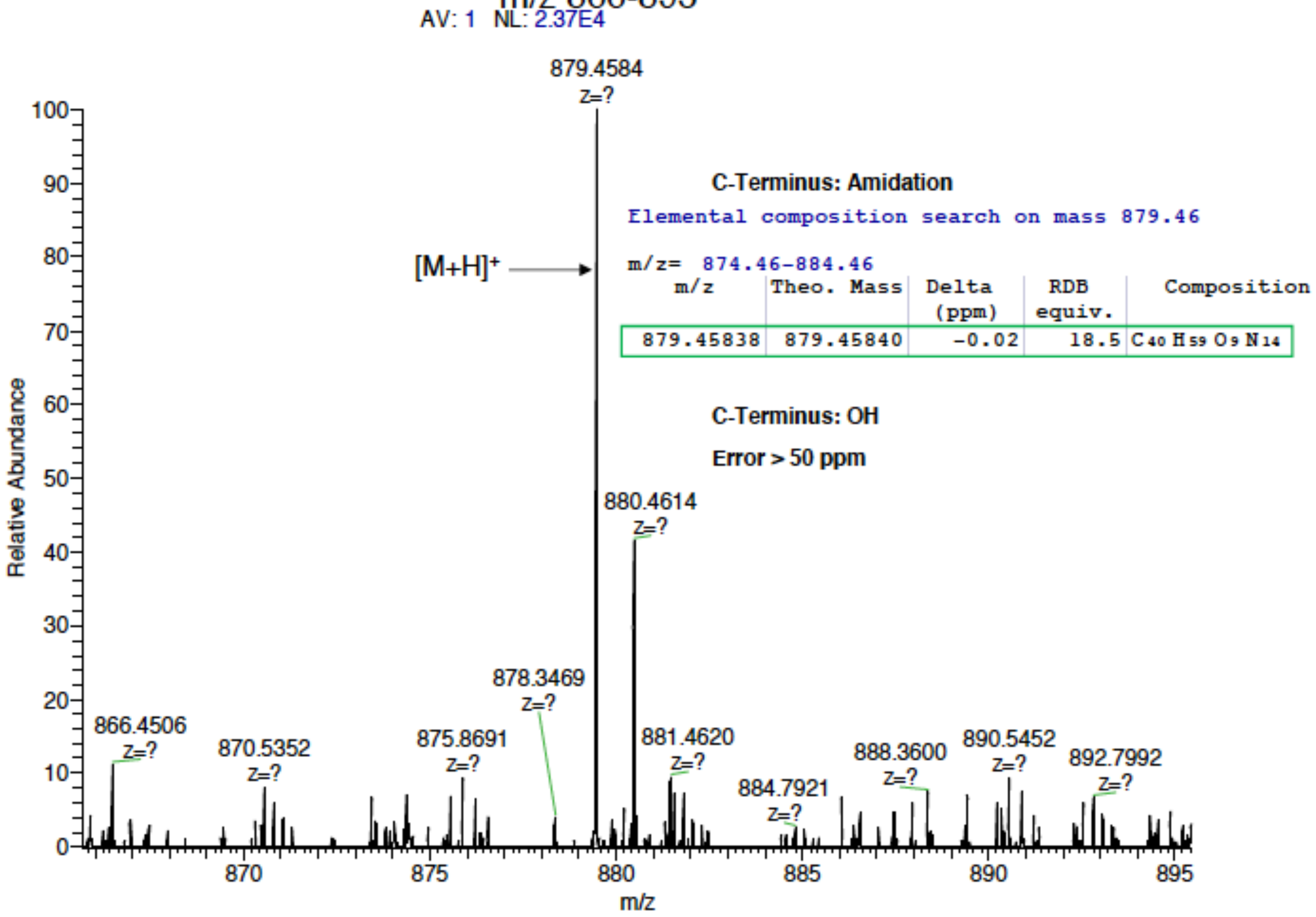

**werrsy**

## Zoom mass spectrum
## m/z 866-895

AV: 1 NL: 1.75E4

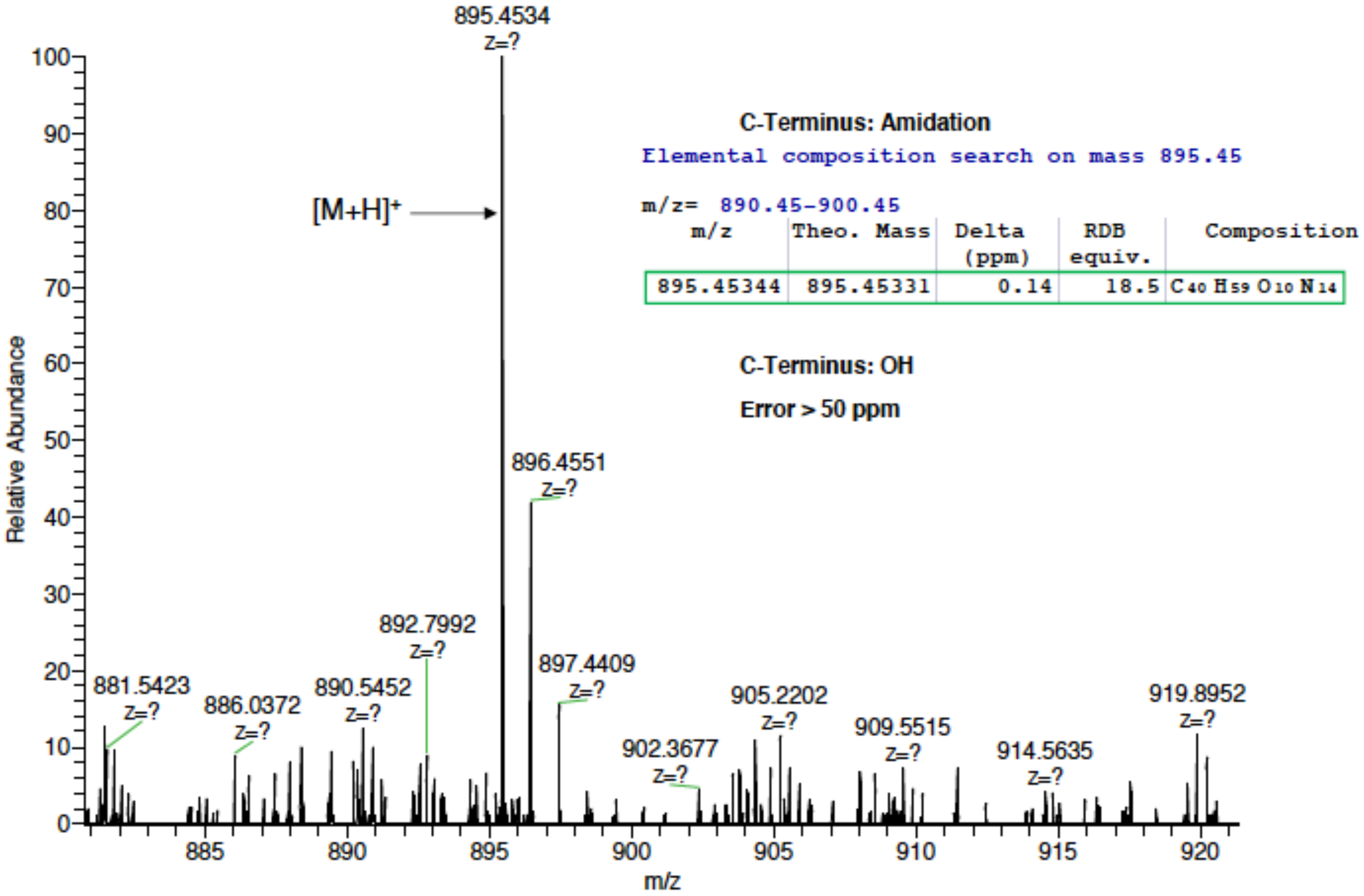

Supplement: Supplementary file 1 [file molecules-25-03316-s001.zip › SM 2_Kalafatovic.pdf]
