# Supplementary material for: Bottom-Up Design Approach for OBOC Peptide Libraries
Source: Molecules. 2020 Jul 22;25(15):3316. doi: 10.3390/molecules25153316 (PMC7435479; doi:10.3390/molecules25153316)
Supplement: Supplementary file 1 [file molecules-25-03316-s001.zip › SM_Kalafatovic.docx]

Supplementary Materials

**Bottom-up design approach for OBOC peptide libraries**

Daniela Kalafatovic^1,*^, Goran Mauša^2^, Dina Rešetar Maslov^1^ and Ernest Giralt^3, 4^

^1^ Department of Biotechnology, University of Rijeka, Radmile Matejcic 2, 51000 Rijeka, Croatia; [daniela.kalafatovic@uniri.hr](mailto:daniela.kalafatovic@uniri.hr), dina.resetar@biotech.uniri.hr

^2^ Faculty of Engineering, University of Rijeka, Vukovarska 58, 51000 Rijeka, Croatia; [gmausa@riteh.hr](mailto:gmausa@riteh.hr)

^3^ Institute for Research in Biomedicine (IRB Barcelona), The Barcelona Institute of Science and Technology (BIST), Baldiri Reixac, 10, 08028 Barcelona, Spain; [ernest.giralt@irbbarcelona.org](mailto:ernest.giralt@irbbarcelona.org)

^4^ Department of Inorganic and Organic Chemistry, University of Barcelona, Marti i Franques, 1- 5, 08028 Barcelona, Spain

* Correspondence: daniela.kalafatovic@uniri.hr; Tel.: +385 51 584 588


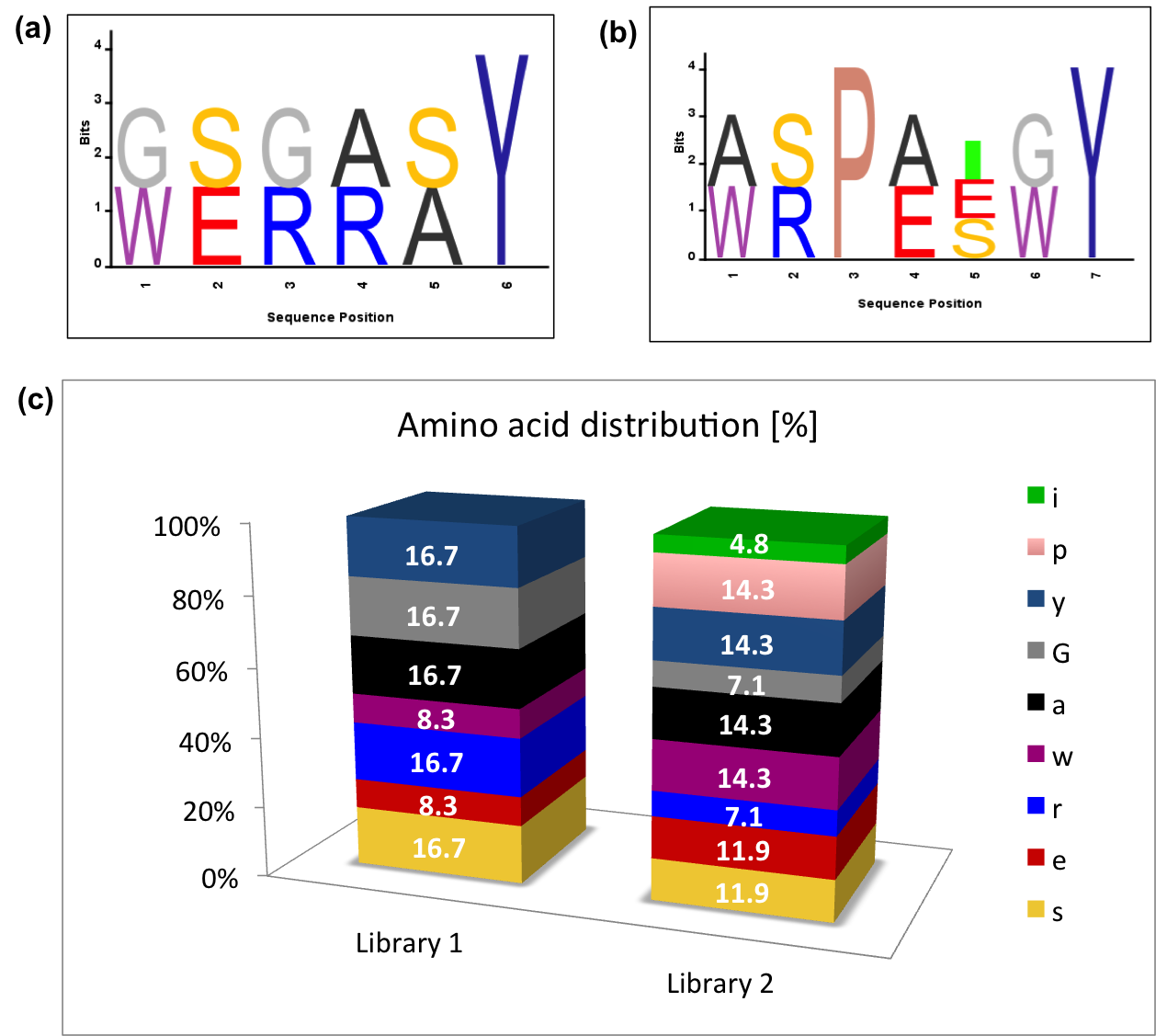


Figure S1. Sequence logos representing the amino acid composition of **(a)** Library 1 and **(b)** Library 2 alongside the **(c)** calculated percentages of each amino acid used to obtain the mentioned libraries.


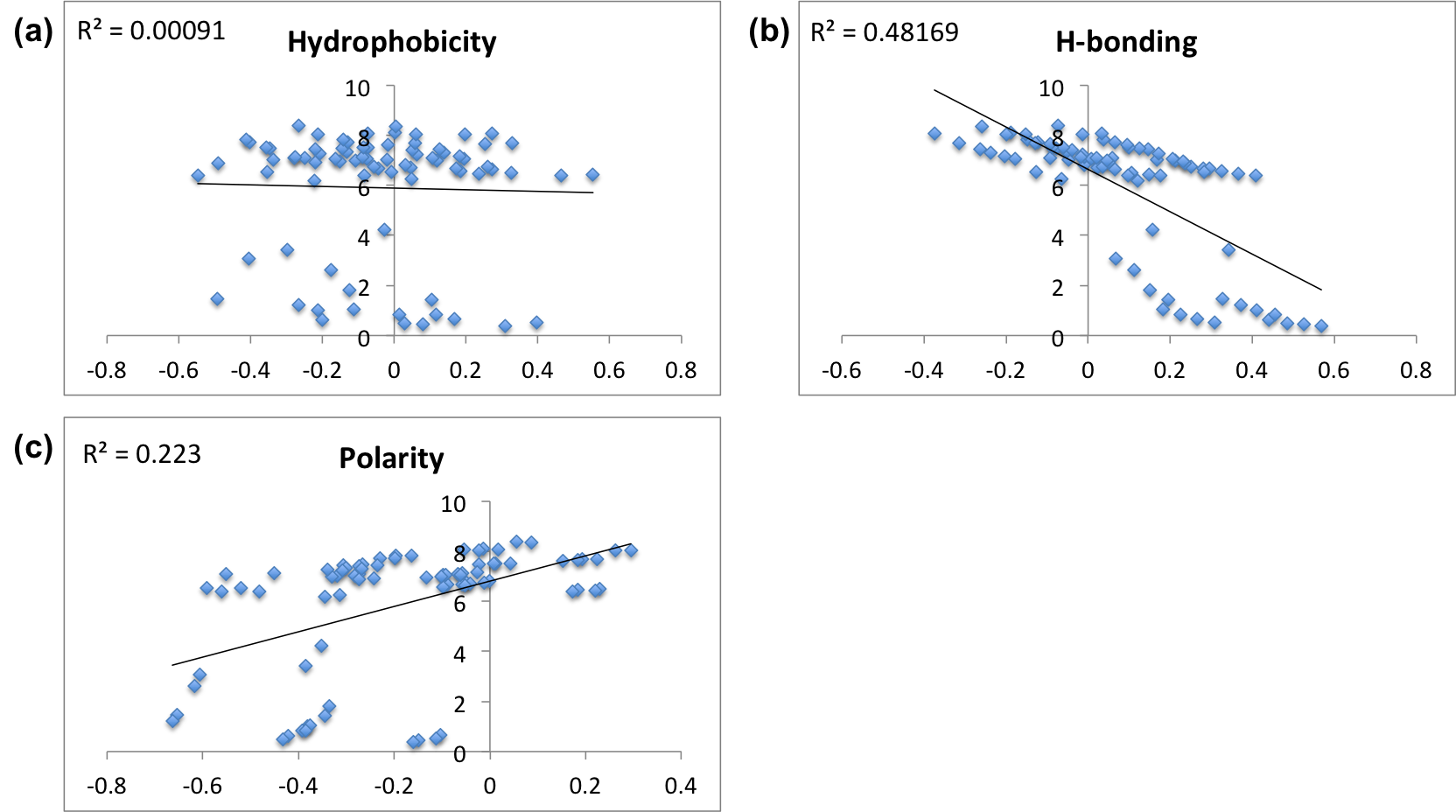


Figure S2. Linear regression based relationship between experimentally (UPLC) determined retention times (Rt) and Cruciani properties calculated with R.


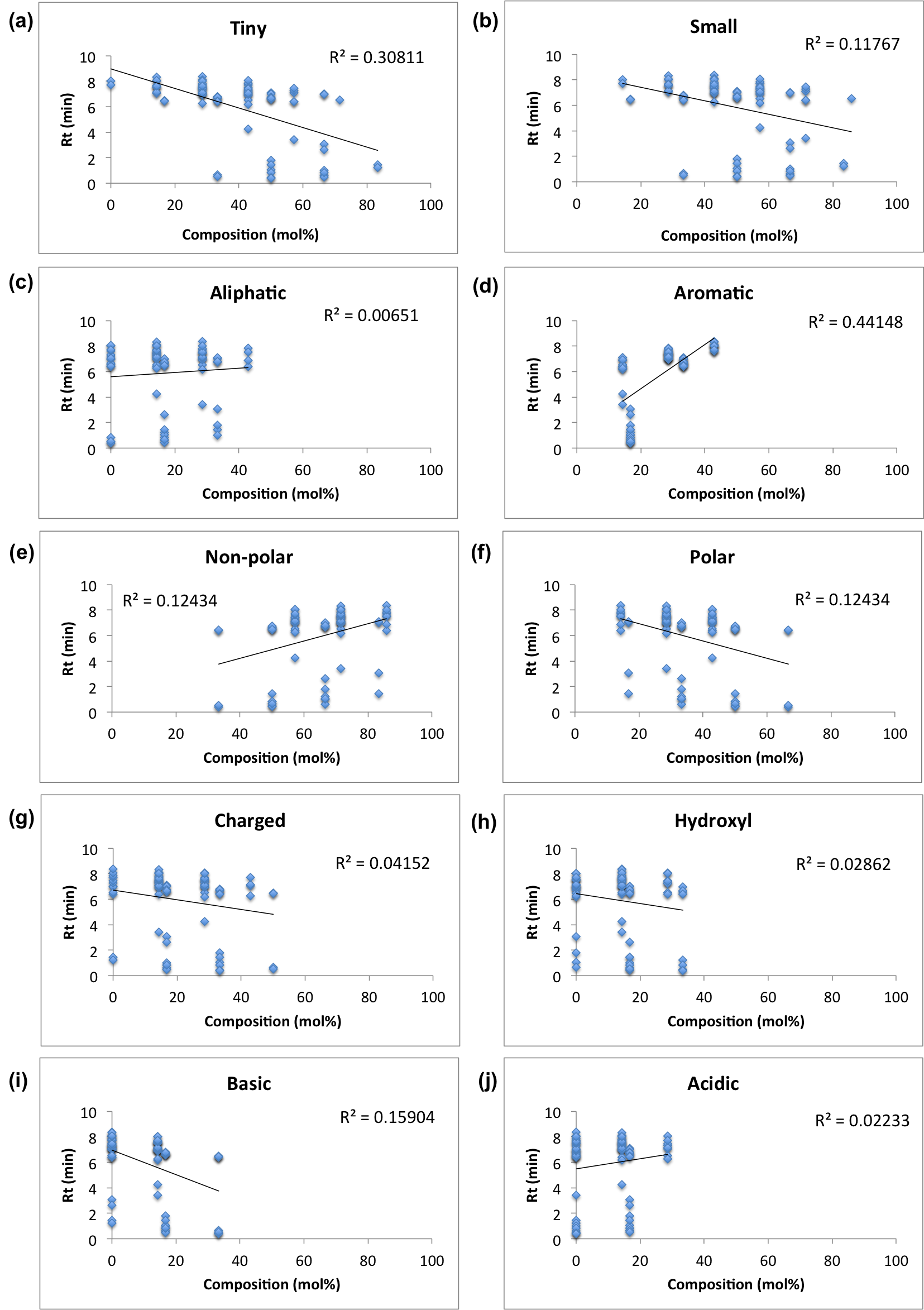


Figure S3. Linear regression based relationship between experimentally (UPLC) determined retention times (Rt) and library composition calculated with R.


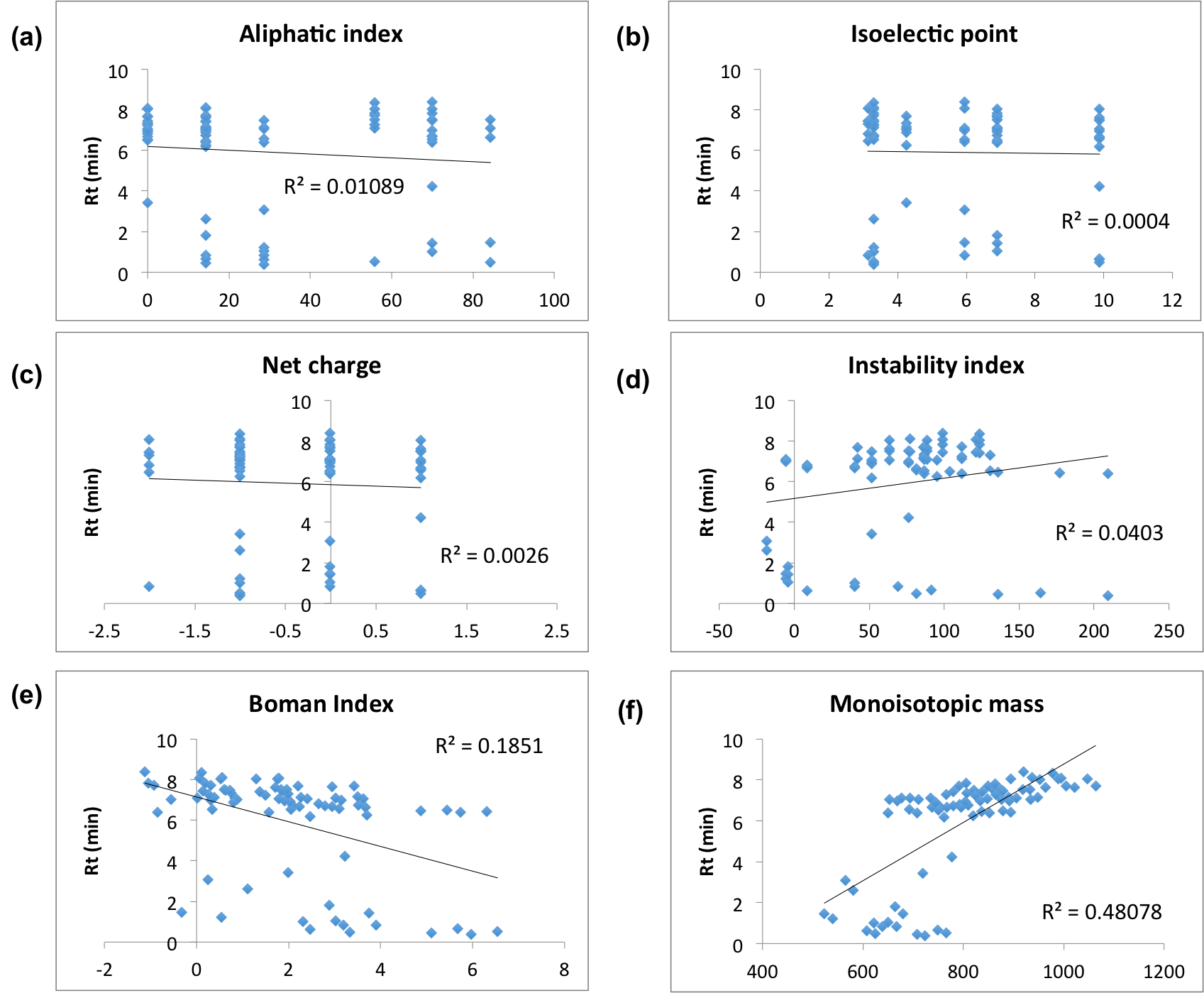


Figure S4. Linear regression based relationship between experimentally (UPLC) determined retention times (Rt) and library properties: **(a)** aliphatix index, **(b**) isoelectric point, **(c)** net charge, **(d)** instability index, **(e)** Boman index calculated with R and **(f**) monoisotopic mass.


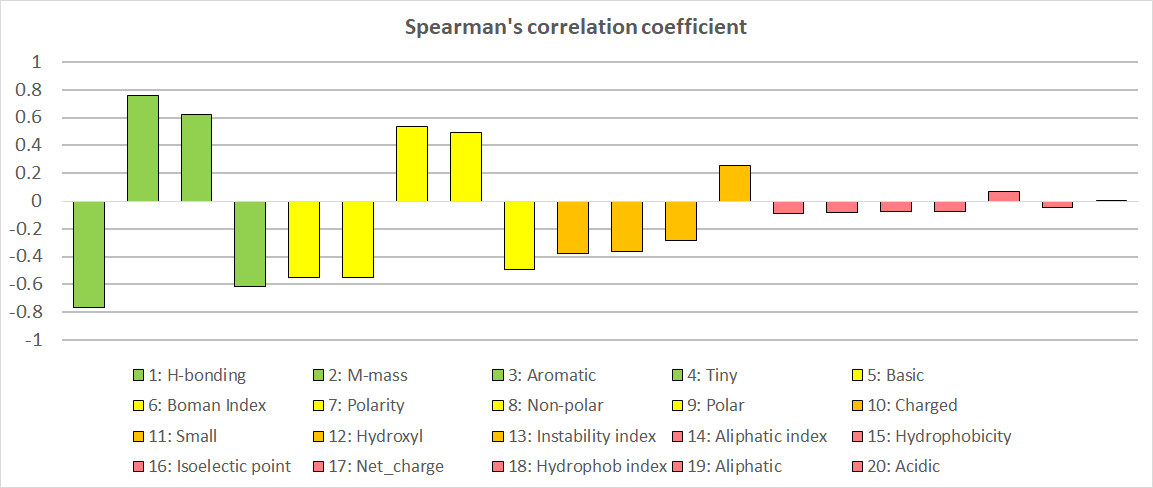


Figure S5. Nonparametric statistical measure of Spearman’s correlation coefficient (ρ), constrained in the range [-1, 1] interpreted according to its absolute value as very strong (1.0 - 0.8), strong (0.8 - 0.6), moderate (0.6 - 0.4), weak (0.4 - 0.2) and very weak (0.2 - 0.0).


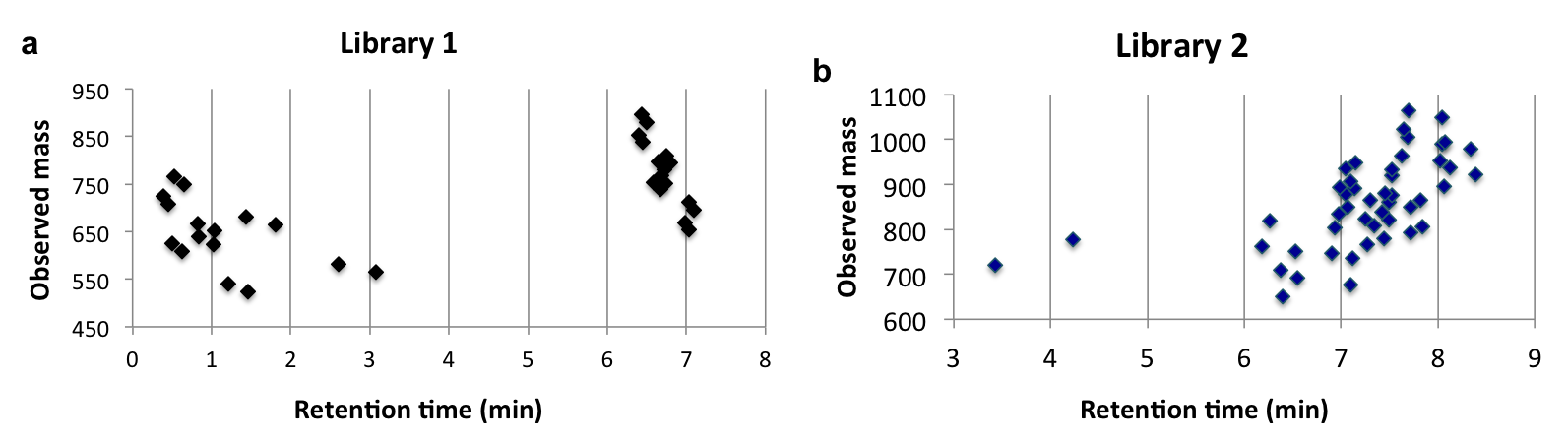


Figure S6. Retention time regions, being (a) 0 to 4 minutes and 6 to 8 minutes for L1 and (b) 2.5 to 5.5 min and 6 to 9 min for L2, representing the time frames where all the peptides were detected using UPLC-MS.

Table S1. List of all the amino *acid permutations in library 1.*

| Entry | Permutation | Monoisotopic mass | Expected [M+H] | Rt (min) | UPLC-MS Observed mass | MS- observed mass |
| --- | --- | --- | --- | --- | --- | --- |
| 1 | GsGaay | 523.2391 | 524.2469 | 1.46 | 524.12 | 524.2464 |
| 2 | GsGasy | 539.2340 | 540.2418 | 1.21 | 540.00 | 540.2413 |
| 3 | GeGaay | 565.2496 | 566.2575 | 3.08 | 566.07 | 566.2569 |
| 4 | GeGasy | 581.2446 | 582.2524 | 2.61 | 582.01 | 582.2519 |
| 5 | GsGray | 608.3032 | 609.3110 | 0.62 | 609.14 | 609.3103 |
| 6 | Gsraay | 622.3188 | 623.3267 | 1.02 | 623.14 | 623.3260 |
| 7 | GsGrsy | 624.2981 | 625.3059 | 0.50 | 625.20 | 625.3054 |
| 8 | Gsrasy | 638.3137 | 639.3216 | 0.84 | 639.08 | 639.3209 |
| 9 | GeGray | 650.3137 | 651.3216 | 1.04 | 651.08 | 651.3210 |
| 10 | wsGaay | 652.2970 | 653.3048 | 7.03 | 653.14 | 653.3024 |
| 11 | Geraay | 664.3294 | 665.3372 | 1.81 | 665.15 | 665.3366 |
| 12 | GeGrsy | 666.3086 | 667.3165 | 0.82 | 667.02 | 667.3158 |
| 13 | wsGasy | 668.2919 | 669.2997 | 6.98 | 669.21 | 669.2990 |
| 14 | Gerasy | 680.3243 | 681.3321 | 1.44 | 681.21 | 681.3316 |
| 15 | weGaay | 694.3075 | 695.3153 | 7.10 | 695.09 | 695.3147 |
| 16 | Gsrray | 707.3829 | 708.3907 | 0.45 | 708.22 | 708.3902 |
| 17 | weGasy | 710.3024 | 711.3103 | 7.03 | 711.22 | 711.3098 |
| 18 | Gsrrsy | 723.3778 | 724.3856 | 0.39 | 724.41 | 724.3849 |
| 19 | wsGray | 737.3610 | 738.3689 | 6.67 | 738.10 | 738.3683 |
| 20 | Gerray | 749.3935 | 750.4013 | 0.65 | 750.23 | 750.4008 |
| 21 | wsraay | 751.3767 | 752.3845 | 6.73 | 752.23 | 752.3837 |
| 22 | wsGrsy | 753.3560 | 754.3638 | 6.58 | 754.23 | 754.3628 |
| 23 | Gerrsy | 765.3884 | 766.3962 | 0.52 | 766.23 | 766.3953 |
| 24 | wsrasy | 767.3716 | 768.3794 | 6.68 | 768.29 | 768.3787 |
| 25 | weGray | 779.3716 | 780.3794 | 6.72 | 780.17 | 780.3787 |
| 26 | weraay | 793.3872 | 794.3951 | 6.80 | 794.17 | 794.3943 |
| 27 | weGrsy | 795.3665 | 796.3743 | 6.65 | 796.17 | 796.3732 |
| 28 | werasy | 809.3822 | 810.3900 | 6.75 | 810.18 | 810.3894 |
| 29 | wsrray | 836.4408 | 837.4486 | 6.45 | 837.24 | 837.4477 |
| 30 | wsrrsy | 852.4357 | 853.4435 | 6.40 | 853.06 | 853.4427 |
| 31 | werray | 878.4513 | 879.4592 | 6.50 | 879.13 | 879.4584 |
| 32 | werrsy | 894.4462 | 895.4541 | 6.43 | 895.19 | 895.4534 |


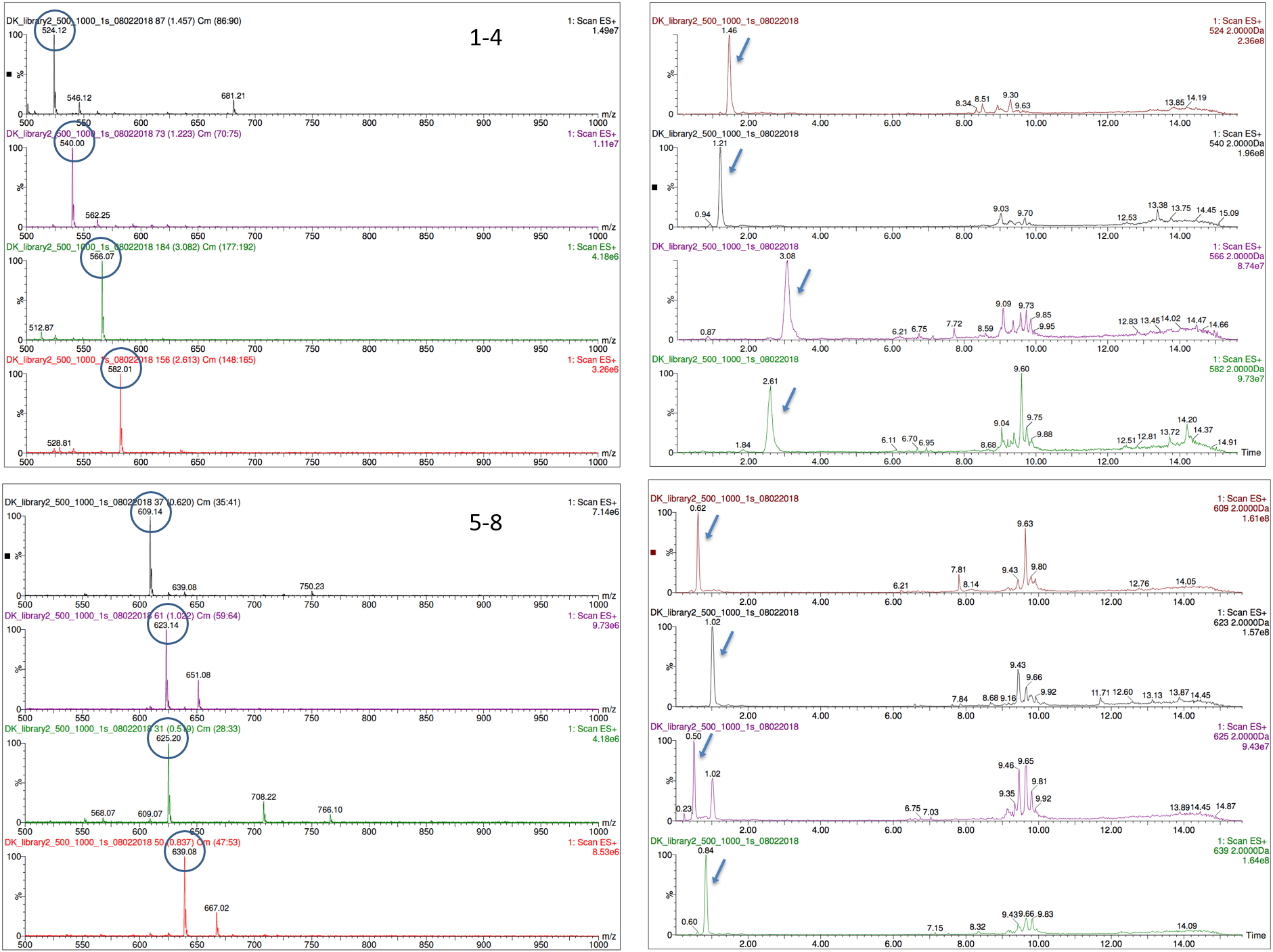


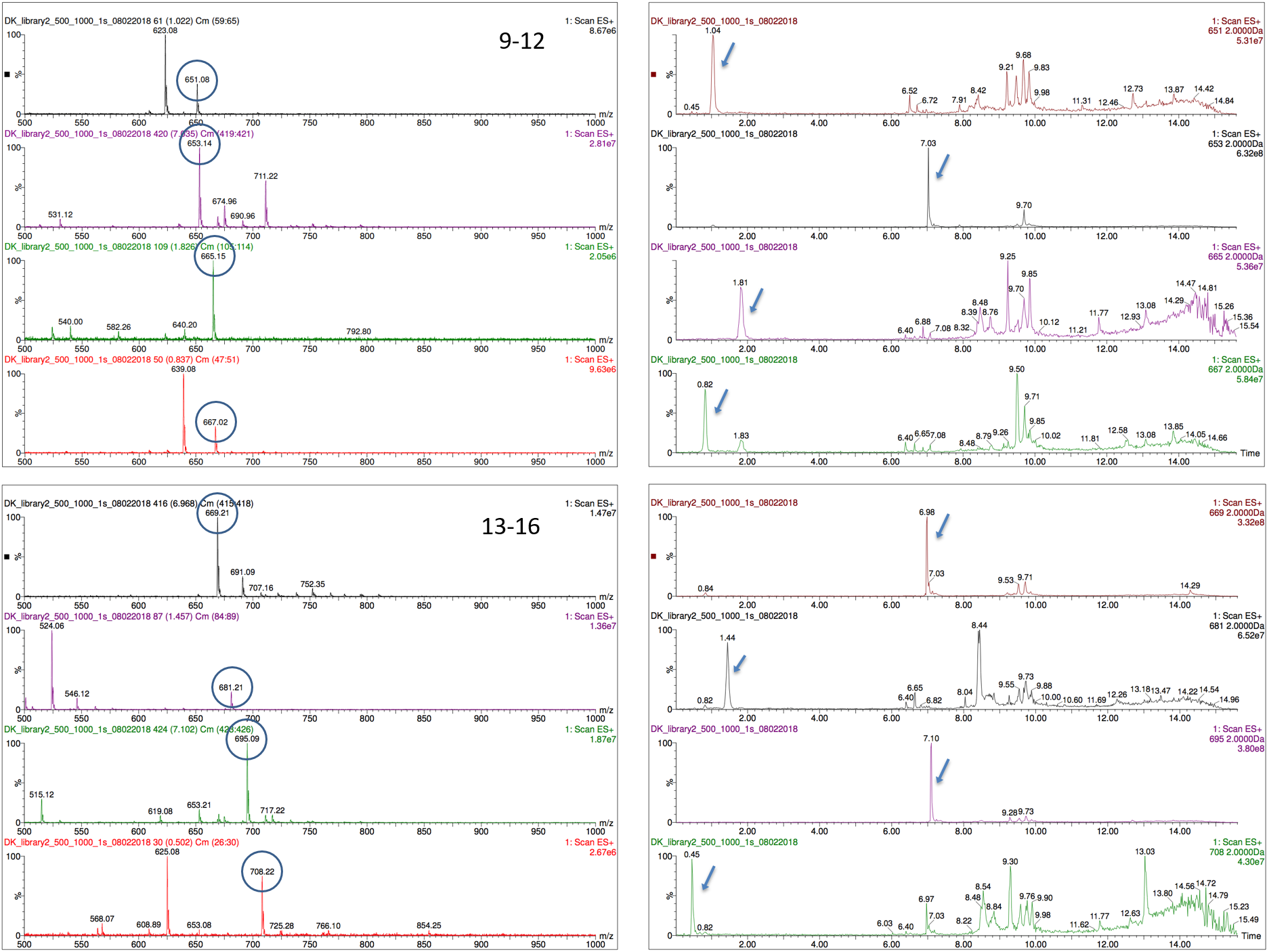


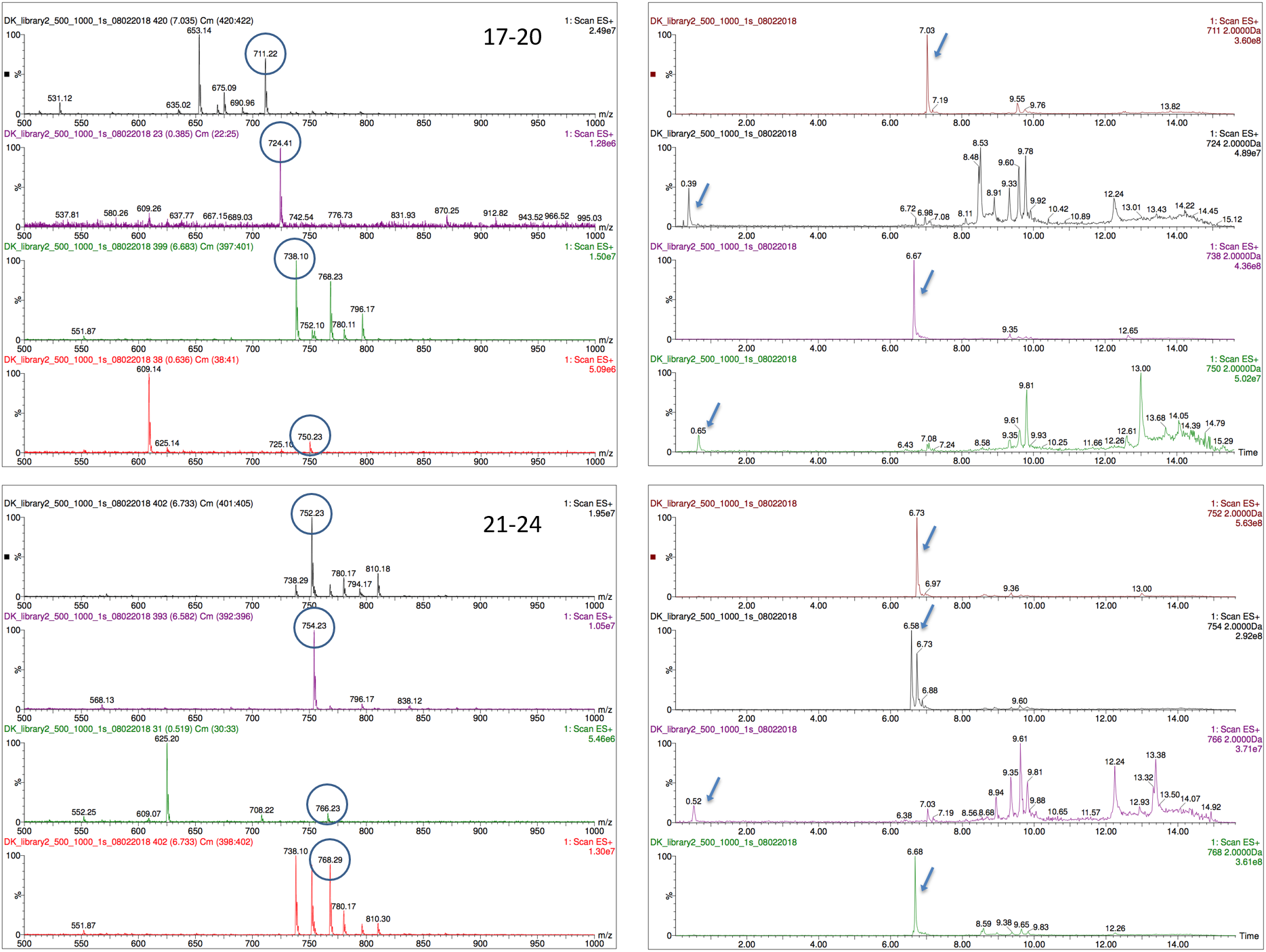

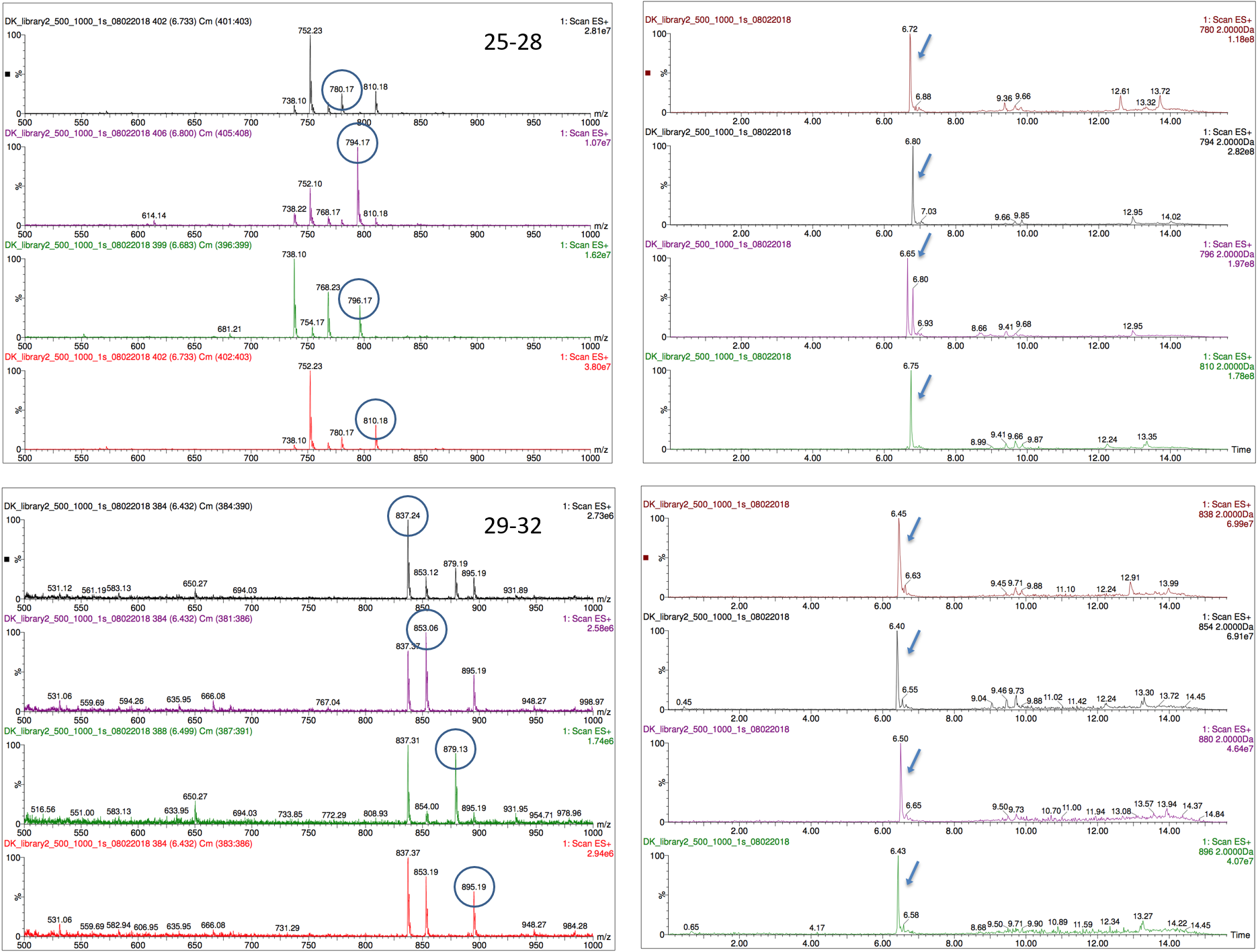


Figure S7. PDA and TIC chromatograms for all the 32 peptides detected using UPLC-MS.

Table S2. List of all the amino acid permutations in library 2.

| Entry | Permutations | Monoisotopic mass | Expected [M+H] | Rt (min) | UPLC-MS Observed mass |
| --- | --- | --- | --- | --- | --- |
| 1 | aspasGy | 650.3024 | 651.3103 | 6.40 | 651.09 |
| 2 | aspaiGy | 676.3545 | 677.3623 | 7.10 | 677.18 |
| 3 | aspaeGy | 692.313 | 693.3208 | 6.55 | 693.06 |
| 4 | aspesGy | 708.3079 | 709.3157 | 6.38 | 709.06 |
| 5 | arpasGy | 719.3716 | 720.3794 | 3.43 | 720.22 |
| 6 | aspeiGy | 734.3599 | 735.3677 | 7.12 | 735.15 |
| 7 | arpaiGy | 745.4237 | 746.4315 | 6.90 | 746.24 |
| 8 | aspeeGy | 750.3184 | 751.3262 | 6.53 | 751.09 |
| 9 | arpaeGy | 761.3822 | 762.3900 | 6.18 | 762.18 |
| 10 | wspasGy | 765.3446 | 766.3525 | 7.27 | 766.21 |
| 11 | arpesGy | 777.377 | 778.3849 | 4.23 | 778.25 |
| 12 | aspaswy | 779.3603 | 780.3681 | 7.44 | 780.07 |
| 13 | wspaiGy | 791.3967 | 792.4045 | 7.72 | 792.24 |
| 14 | arpeiGy | 803.4291 | 804.4369 | 6.93 | 804.21 |
| 15 | aspaiwy | 805.4123 | 806.4202 | 7.84 | 806.22 |
| 16 | wspaeGy | 807.3552 | 808.363 | 7.34 | 808.11 |
| 17 | arpeeGy | 819.3876 | 820.3954 | 6.26 | 820.15 |
| 18 | aspaewy | 821.3708 | 822.3787 | 7.49 | 822.23 |
| 19 | wspesGy | 823.3501 | 824.3579 | 7.25 | 824.18 |
| 20 | wrpasGy | 834.4138 | 835.4217 | 6.97 | 835.27 |
| 21 | aspeswy | 837.3657 | 838.3736 | 7.42 | 838.17 |
| 22 | arpaswy | 848.4295 | 849.4373 | 7.07 | 849.20 |
| 23 | wspeiGy | 849.4022 | 850.4100 | 7.72 | 850.21 |
| 24 | wrpaiGy | 860.4659 | 861.4737 | 7.49 | 861.30 |
| 25 | aspeiwy | 863.4178 | 864.4256 | 7.82 | 864.13 |
| 26 | wspeeGy | 865.3606 | 866.3685 | 7.30 | 866.21 |
| 27 | arpaiwy | 874.4815 | 875.4894 | 7.52 | 875.22 |
| 28 | wrpaeGy | 876.4244 | 877.4322 | 7.05 | 877.24 |
| 29 | aspeewy | 879.3763 | 880.3841 | 7.45 | 880.14 |
| 30 | arpaewy | 890.4400 | 891.4479 | 7.14 | 891.16 |
| 31 | wrpesGy | 892.4193 | 893.4271 | 6.98 | 893.24 |
| 32 | wspaswy | 894.4025 | 895.4103 | 8.06 | 895.20 |
| 33 | arpeswy | 906.4349 | 907.4427 | 7.10 | 907.23 |
| 34 | wrpeiGy | 918.4713 | 919.4791 | 7.52 | 919.20 |
| 35 | wspaiwy | 920.4546 | 921.4624 | 8.39 | 921.22 |
| 36 | arpeiwy | 932.4870 | 933.4948 | 7.52 | 933.25 |
| 37 | wrpeeGy | 934.4298 | 935.4376 | 7.05 | 935.27 |
| 38 | wspaewy | 936.4131 | 937.4209 | 8.12 | 937.16 |
| 39 | arpeewy | 948.4455 | 949.4533 | 7.15 | 949.20 |
| 40 | wspeswy | 952.4080 | 953.4158 | 8.02 | 953.10 |
| 41 | wrpaswy | 963.4717 | 964.4795 | 7.62 | 964.26 |
| 42 | wspeiwy | 978.46 | 979.4679 | 8.34 | 979.19 |
| 43 | wrpaiwy | 989.5237 | 990.5316 | 8.04 | 990.34 |
| 44 | wspeewy | 994.4185 | 995.4263 | 8.07 | 995.26 |
| 45 | wrpaewy | 1005.4822 | 1006.4901 | 7.69 | 1006.22 |
| 46 | wrpeswy | 1021.4771 | 1022.485 | 7.64 | 1022.29 |
| 47 | wrpeiwy | 1047.5292 | 1048.537 | 8.04 | 1048.19 |
| 48 | wrpeewy | 1063.4877 | 1064.4955 | 7.70 | 1064.19 |


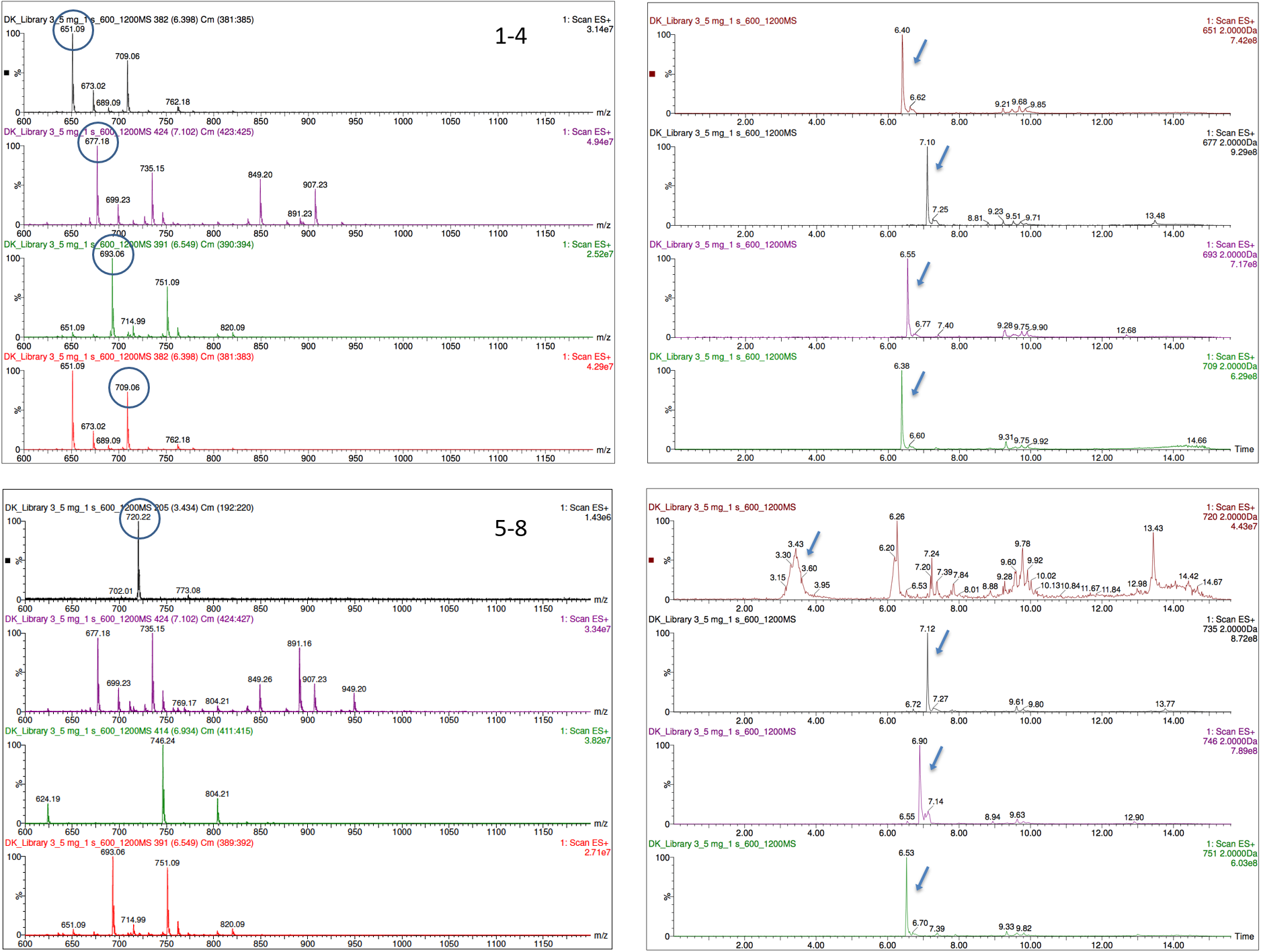


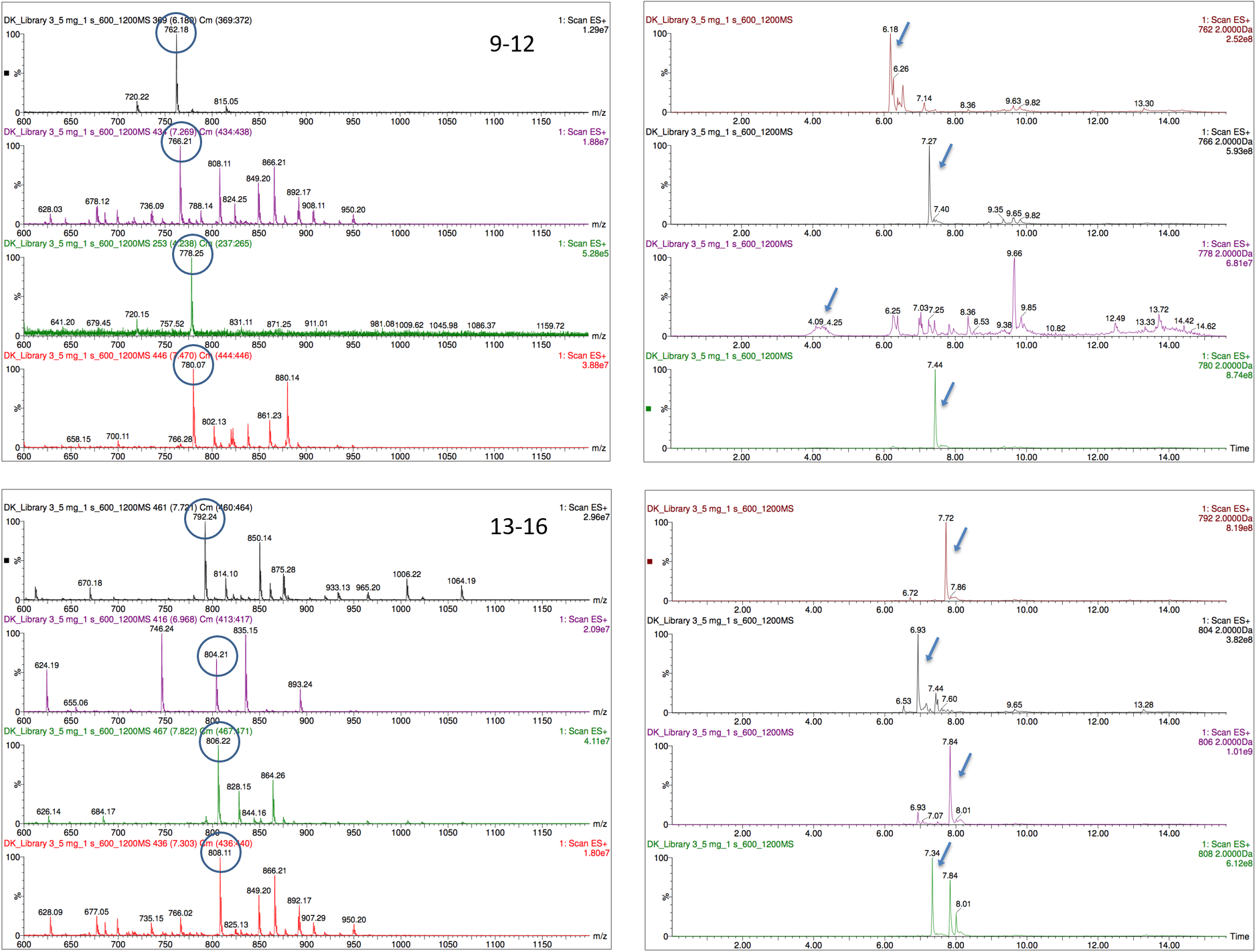

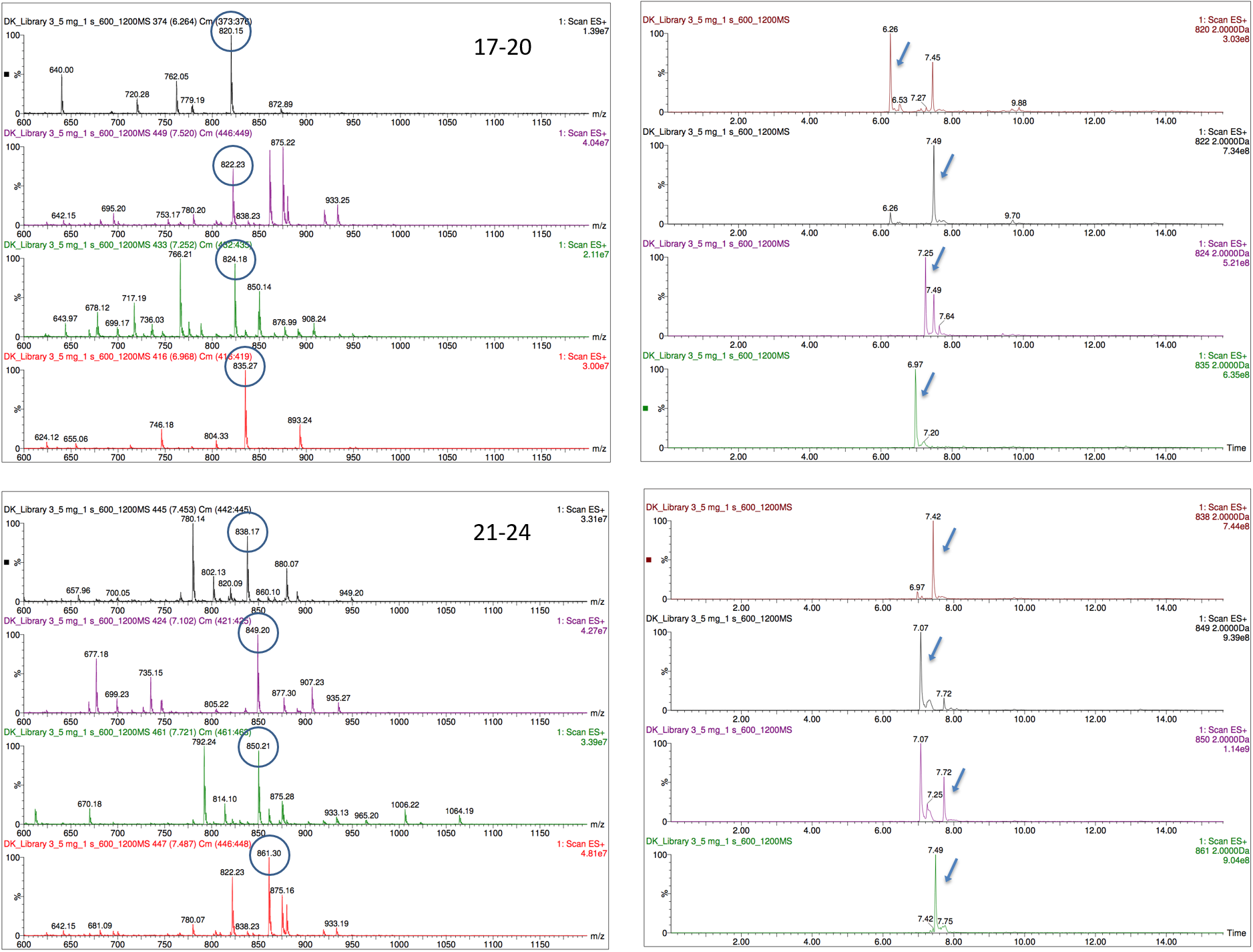

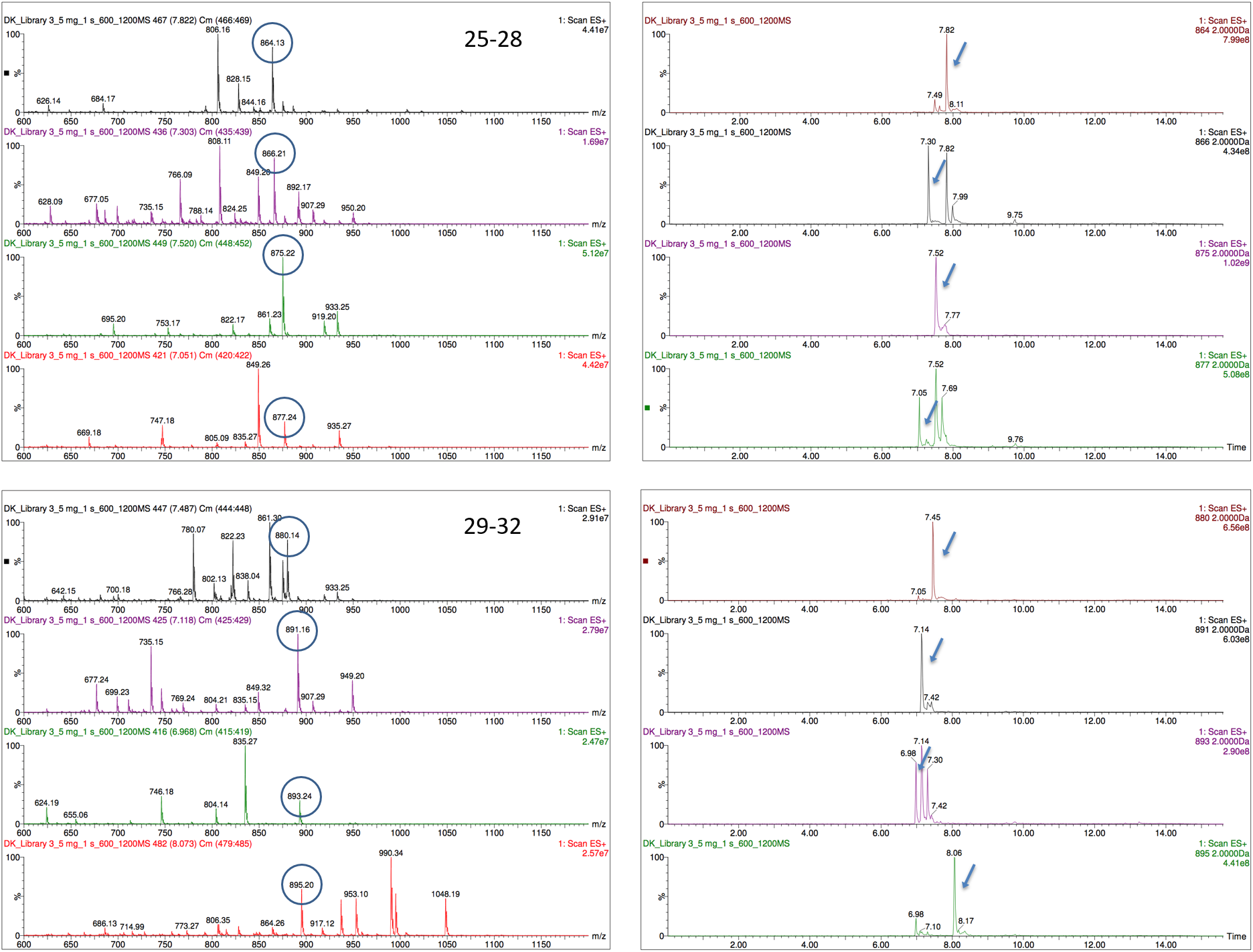

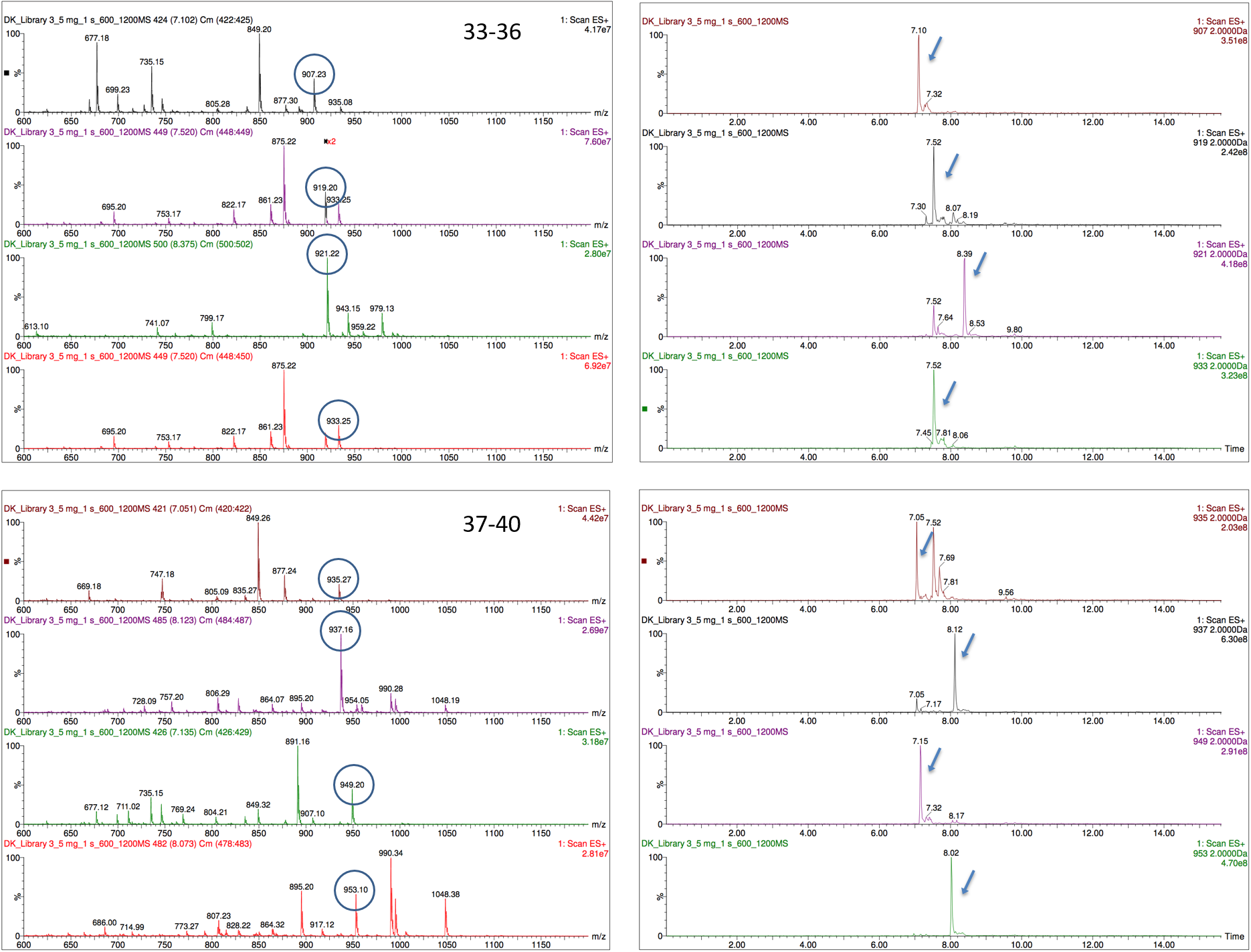

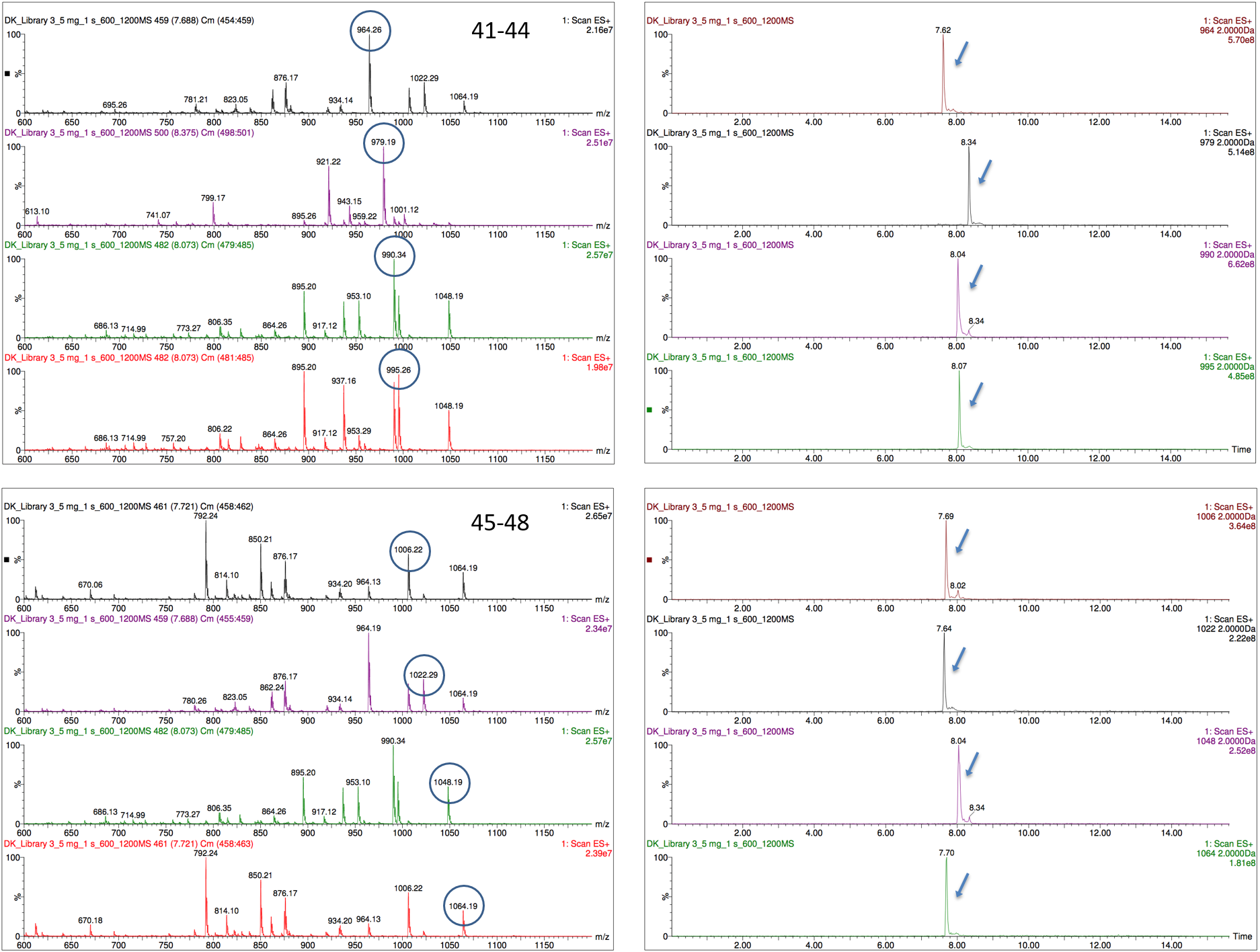


Figure S8. PDA and TIC chromatograms for all the 48 peptides detected using UPLC-MS.


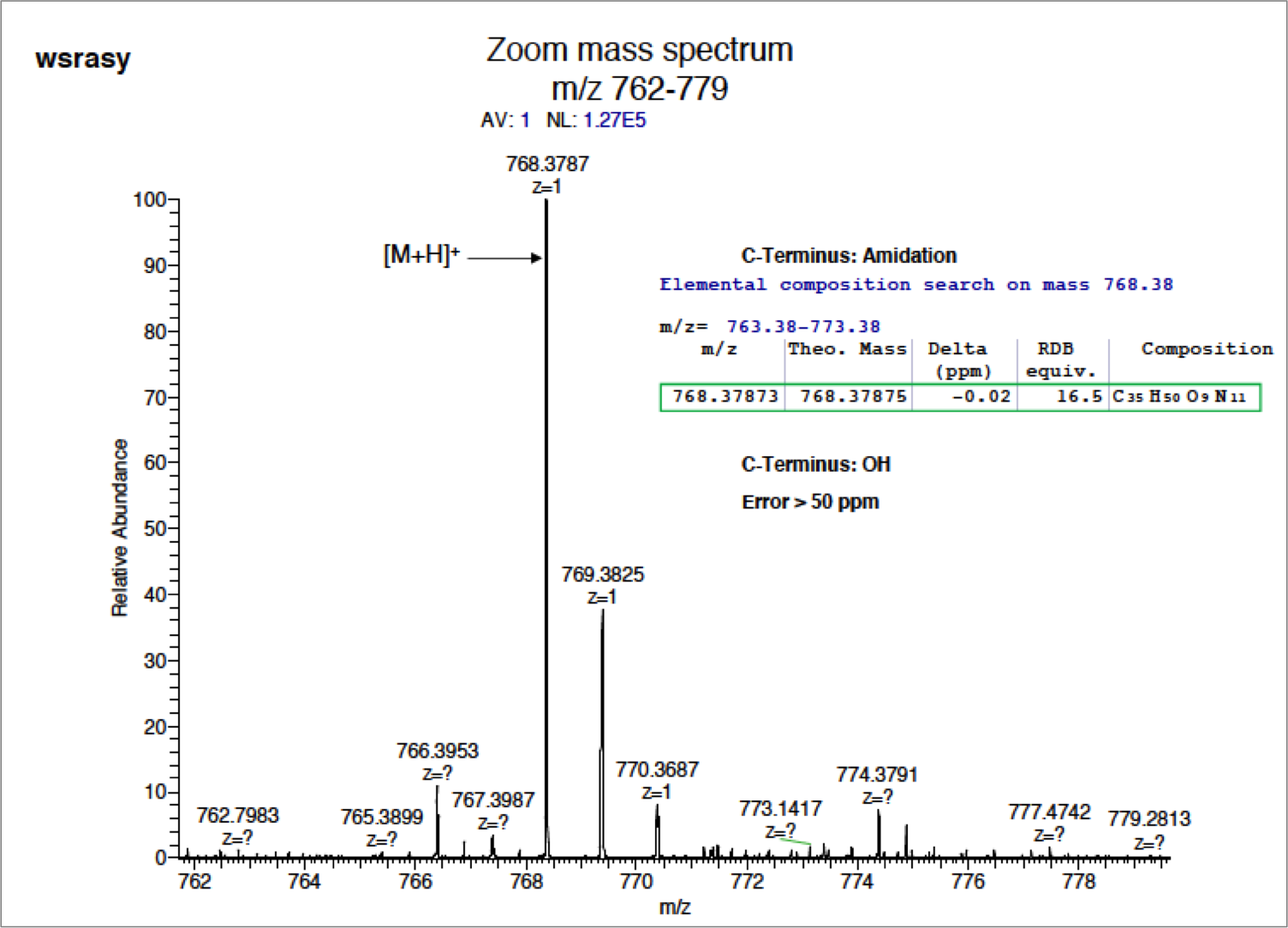


Figure S9. High resolution MS spectrum of *wsrasy*.


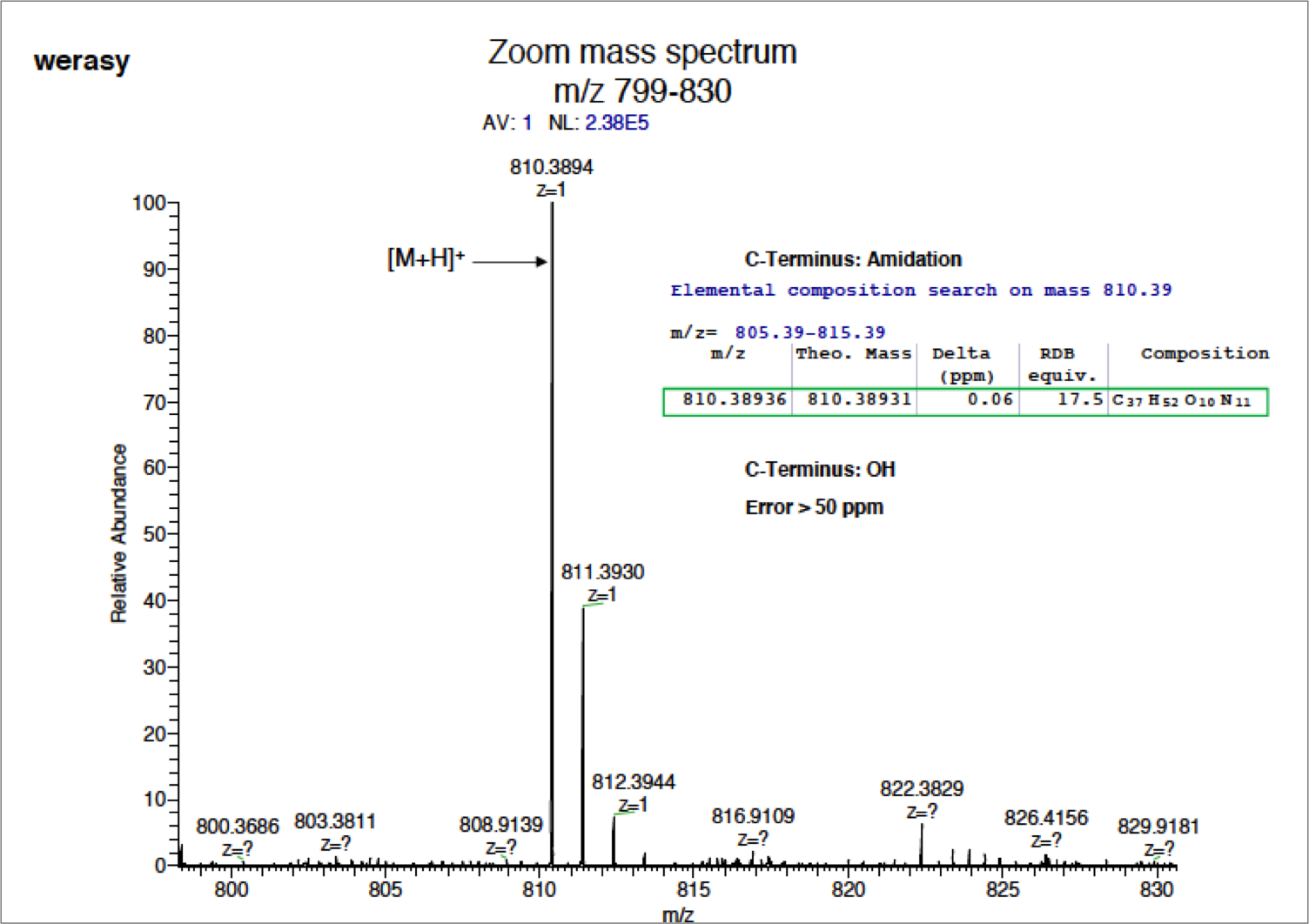


Figure S10. High resolution MS spectrum of *werasy*.


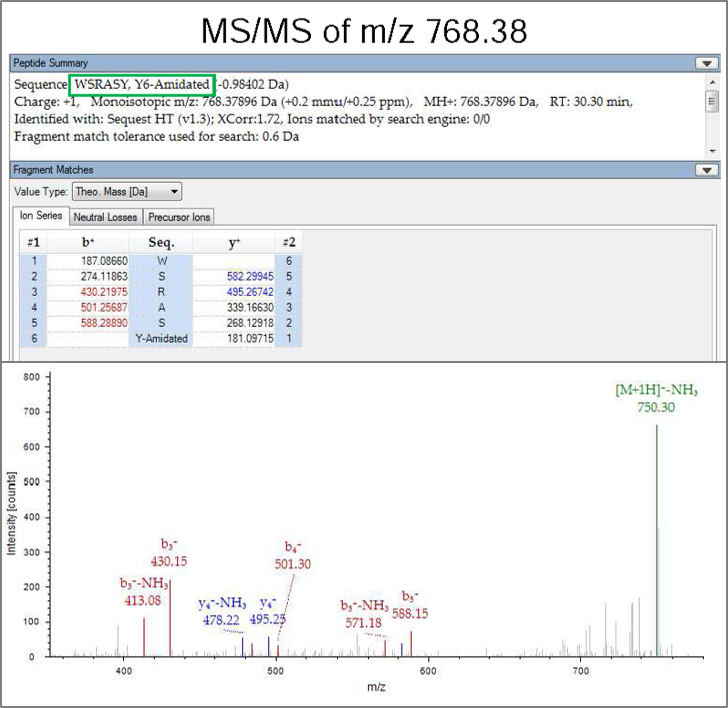


Figure S11. The identified MS/MS peaks, yielding b- (red) and y- (blue) type ions for *wsrasy*.


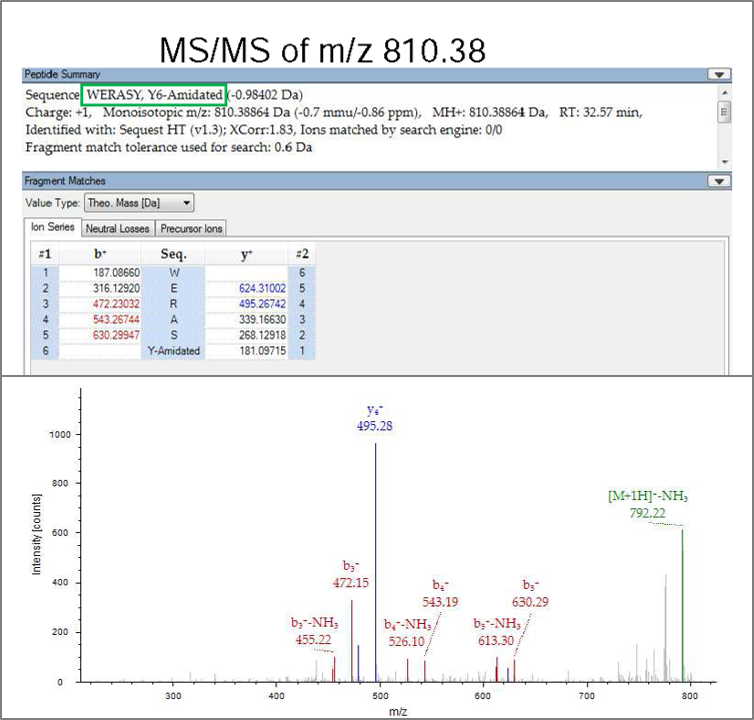


Figure S12. The identified MS/MS peaks, yielding b- (red) and y- (blue) type ions for *werasy*.
